# Comprehensive characterization of the neurogenic and neuroprotective action of a novel TrkB agonist using mouse and human stem cell models of Alzheimer’s disease

**DOI:** 10.1186/s13287-024-03818-w

**Published:** 2024-07-06

**Authors:** Despoina Charou, Thanasis Rogdakis, Alessia Latorrata, Maria Valcarcel, Vasileios Papadogiannis, Christina Athanasiou, Alexandros Tsengenes, Maria Anna Papadopoulou, Dimitrios Lypitkas, Matthieu D. Lavigne, Theodora Katsila, Rebecca C. Wade, M. Zameel Cader, Theodora Calogeropoulou, Achille Gravanis, Ioannis Charalampopoulos

**Affiliations:** 1 Department of Pharmacology, Medical School, University of Crete, 71003 Heraklion, Greece; 2 Foundation for Research and Technology-Hellas (IMBB-FORTH), Institute of Molecular Biology and Biotechnology, 70013 Heraklion, Greece; 3 Institute of Chemical Biology, National Hellenic Research Foundation, 11635 Athens, Greece; 4 Innovative Technologies in Biological Systems SL (INNOPROT), 48160 Derio, Bizkaia Spain; 5 Hellenic Centre for Marine Research (HCMR), Institute of Marine Biology Biotechnology and Aquaculture (IMBBC), Heraklion, Crete Greece; 6 Translational Molecular Neuroscience Group, Dorothy Crowfoot Hodgkin Building, Kavli Institute for Nanoscience, Nuffield Department of Clinical Neurosciences, University of Oxford, Oxford, UK; 7 Molecular and Cellular Modeling Group, Heidelberg Institute for Theoretical Studies (HITS), 69118 Heidelberg, Germany; 8 Faculty of Biosciences, Heidelberg University, 69120 Heidelberg, Germany; 9 Heidelberg Biosciences International Graduate School, Heidelberg University, 69120 Heidelberg, Germany; 10 Center for Molecular Biology (ZMBH), DKFZ-ZMBH Alliance, Heidelberg University, 69120 Heidelberg, Germany; 11 Interdisciplinary Center for Scientific Computing (IWR), Heidelberg University, 69120 Heidelberg, Germany

**Keywords:** In-vitro drug testing, Stem-cell based screening, BDNF, TrkB, Neurogenesis, iPSC, Alzheimer’s disease

## Abstract

**Background:**

Neural stem cell (NSC) proliferation and differentiation in the mammalian brain decreases to minimal levels postnatally. Nevertheless, neurogenic niches persist in the adult cortex and hippocampus in rodents, primates and humans, with adult NSC differentiation sharing key regulatory mechanisms with development. Adult neurogenesis impairments have been linked to Alzheimer’s disease (AD) pathology. Addressing these impairments by using neurotrophic factors is a promising new avenue for therapeutic intervention based on neurogenesis. However, this possibility has been hindered by technical difficulties of using in-vivo models to conduct screens, including working with scarce NSCs in the adult brain and differences between human and mouse models or ethical limitations.

**Methods:**

Here, we use a combination of mouse and human stem cell models for comprehensive in-vitro characterization of a novel neurogenic compound, focusing on the brain-derived neurotrophic factor (BDNF) pathway. The ability of ENT-A011, a steroidal dehydroepiandrosterone derivative, to activate the tyrosine receptor kinase B (TrkB) receptor was tested through western blotting in NIH-3T3 cells and its neurogenic and neuroprotective action were assessed through proliferation, cell death and Amyloid-β (Aβ) toxicity assays in mouse primary adult hippocampal NSCs, mouse embryonic cortical NSCs and neural progenitor cells (NPCs) differentiated from three human induced pluripotent stem cell lines from healthy and AD donors. RNA-seq profiling was used to assess if the compound acts through the same gene network as BDNF in human NPCs.

**Results:**

ENT-A011 was able to increase proliferation of mouse primary adult hippocampal NSCs and embryonic cortical NSCs, in the absence of EGF/FGF, while reducing Aβ-induced cell death, acting selectively through TrkB activation. The compound was able to increase astrocytic gene markers involved in NSC maintenance, protect hippocampal neurons from Αβ toxicity and prevent synapse loss after Aβ treatment. ENT-A011 successfully induces proliferation and prevents cell death after Aβ toxicity in human NPCs, acting through a core gene network shared with BDNF as shown through RNA-seq.

**Conclusions:**

Our work characterizes a novel BDNF mimetic with preferable pharmacological properties and neurogenic and neuroprotective actions in Alzheimer’s disease via stem cell-based screening, demonstrating the promise of stem cell systems for short-listing competitive candidates for further testing.

**Supplementary Information:**

The online version contains supplementary material available at 10.1186/s13287-024-03818-w.

## Introduction

From early embryonic development until early postnatal stages, neural stem cells (NSCs) proliferate, migrate, differentiate and mature to new neurons in a multi-step process known as neurogenesis [1–3]. Neurogenesis drops sharply postnatally, yet many studies have demonstrated neurogenesis in aged brains of rodents, non-human primates and humans [4–11]. More specifically, neurogenesis has been detected in the adult mammalian brain in specific areas known as neurogenic niches, namely the subventricular zone (SVZ) of the cortex and the dentate gyrus of the hippocampus [4, 12, 13]. In pathology, dysfunctional adult neurogenesis has been linked to AD, a neurodegenerative disease that is the most common cause of dementia, characterized by β-amyloid (Aβ) deposition and neurofibrillary tangle formation. The most affected areas in the AD brain are the cortex and the hippocampus and impaired adult hippocampal neurogenesis (AHN) has been shown to occur in early stages of the development of the disease [14–17]. Moreover, neural stem cells and progenitor cells from the AD brain have showed reduced proliferation, viability, differentiation, increased senescence when exposed to a harmful microenvironment and decreased interactions with the neurogenic niche [18–23]. Targeting the mechanisms underlying NSC fate and behavior in AHN has been suggested as a strategy for developing novel therapeutics for neurodegeneration, with various existing drugs highlighted that may be acting through AHN related mechanisms [24].

The molecular and cellular processing underlying adult neurogenesis may differ to those involved in embryonic and postnatal neurogenesis, but there are shared mechanisms at play, such as the role of many trophic factors [25]. Experimental cooption of neurogenic molecular signals in vitro has allowed the development of functional central nervous system (CNS) neurons from stem cells derived from adult hippocampus or astrocytes from postnatal hippocampus. Furthermore, the maturation and synaptogenesis mechanisms used by the neural progeny of adult neural stem cells, including factors contributed by neonatal astrocytes, also share similarities with those during development [26].

Brain-derived neurotrophic factor (BDNF) is a member of the neurotrophin family of trophic factors that binds with high affinity and activates the Tropomyosin receptor kinase B (TrkB), a widely expressed and distributed receptor in the CNS. Many studies have shown the critical role of BDNF and TrkB signaling in adult neurogenesis [27–29], while a reduction of BDNF has been reported in the cortex and the hippocampus of AD [30–33]. BDNF is instrumental in promoting the proliferation, survival, differentiation and functional integration of newborn neurons into hippocampal circuits and the recovery of synaptic degeneration, through the activation of TrkB [22, 29, 34, 35]. Additionally, BDNF has important roles in the function of the adult neurogenic niche, able to increase and maintain its hippocampal levels through a positive feedback loop. Elevated astrocytic BDNF can increase the efficacy of astrocytes in supporting AHN and countering AD deficits, while exogenous BDNF supplementation combined with independent enhancement of AHN can attenuate cognitive decline in AD [26, 36–39]. It is of note that several factors, such as exercise or antidepressant drugs induce adult neurogenesis by increasing the levels of endogenous BDNF [36–40].

Although BDNF has been demonstrated to hold potential in altering AD pathology and counter cognitive decline through its roles in the neurogenic niche and the function of NSCs, its inability to cross the blood–brain barrier (BBB), its poor pharmacokinetic properties and its vulnerability to proteolytic cleavage remain important limitations in using BDNF as a reliable therapeutic agent. This led to the development of small molecules that can activate TrkB signaling and mimic the beneficial properties of BDNF, such as 7,8-dihydroxy flavone (7,8-DHF) and its derivative CF3CN, some of the best studied BDNF mimetics that can prevent neurotoxicity in vitro and in vivo, enhance learning, prevent memory impairment and promote axonal regeneration [41–46]. Other noticeable examples include GSB-106 [47], LM22A-4 [48] that can promote neurite outgrowth and suppress neuronal death in in-vitro neurodegenerative disease models and a derivative of LM22B-10 that is able to increase survival of iPSC-derived cholinergic neurons after exposure to Aβ [49, 50].In addition, derivatives of DHEA, an endogenous neurosteroid produced by neurons and glia in the Central Nervous System (CNS), that bind to and activate neurotrophin receptors have been explored recently [51], including TrkA specific activator ENT-A013, TrkA and p75NTR activator BNN27, and BNN20 which activates TrkA, TrkB and p75NTR [52, 53] and is able to increase BDNF levels through TrkB signaling and exert dopaminergic neuroprotection [54–56].

In this study, we use a combination of rodent and human stem cell systems to comprehensively characterize the neurogenic and neuroprotective potential of ENT-A011, a novel BDNF mimetic targeting the TrkB receptor. The compound upregulates genes crucial for neurogenic niche maintenance, enhancing mouse adult and embryonic NSC proliferation, survival, and mitochondrial activity. ENT-A011 promotes adult NSC differentiation towards neurons and astrocytes and exhibits neuroprotective effects in mouse hippocampal and cortical neurons, including enhanced survival, synaptic protection, and neurite outgrowth. Importantly, the neurogenic impact of the compound extends to human iPSC-derived NSCs, increasing proliferation and countering Aβ-induced cell death, while RNA sequencing reveals ENT-A011 acts on a core gene network shared with BDNF in human NSCs. Serving as a case-study, this work underscores the potential of stem cell models for in-vitro drug testing of new therapeutics for neurodegenerative diseases. The cell systems and assays used act as a proxy of AD pathogenesis in neurogenic niches of the hippocampus and cortex, estimating drug efficacy for short-listing promising candidates for follow-up trials. Finally, the focus of the study on in vitro screening is in line with recent efforts and policies towards reducing animal testing. As stem cell and other related systems are maturing, it is crucial to promote such platforms as viable alternatives for initial screening and selection of high-fidelity candidates, aspiring to promote the 3R principles for animal research.

## Materials and methods

### Cell lines

NIH-3T3 cells were stable transfected with human TrkB or TrkC plasmid, provided by Dr C. F. Ibáñez. Cells were cultured in DMEM medium (11965084, Gibco) containing 10% Fetal Bovine Serum (10270106, Gibco), 100 units/mL Penicillin and 0.1 mg/mL Streptomycin (15140122, Gibco) at 5% CO_2_ and 37 °C. Naïve, non-transfected, NIH-3T3 cells were also cultured under the same conditions.

PC12 and HEK293T cells were sourced from LGC Promochem GmbH (Teddington, UK). PC12 cells were grown in DMEM medium (11965084, Gibco, Grand Island, NY, USA) containing 5% Horse Serum (16050122, Gibco, Grand Island, NY, USA), 10% Fetal Bovine Serum (10270106, Gibco, Grand Island, NY, USA), 100 units/mL Penicillin and 0.1 mg/mL Streptomycin (15140122, Gibco, Grand Island, NY, USA) at 5% CO_2_ and 37 °C. HEK293T were grown in DMEM medium (11965084, Gibco, Grand Island, NY, USA) supplemented with 10% Fetal Bovine Serum and they were transfected transiently with the human p75NTR plasmid using lipofectamine.

HEK293 stably expressing kinase NOMAD/tGFP Biosensor (Innoprot), were cultured in DMEM medium (11965084, Gibco) containing 10% Fetal Bovine Serum (10270106, Gibco), 100 units/mL Penicillin and 0.1 mg/mL Streptomycin (15140122, Gibco) and 1X MEM Non-Essential Amino Acids Solution (11140050, Gibco) at 5% CO_2_ and 37 °C. Cells were transiently transfected with cDNA plasmid encoding TrkB using calcium phosphate transfection method.

### Animals

Mouse primary cells were isolated from C57BL/6 J mice (Charles River Laboratories) and rat primary neurons from Sprague–Dawley rat fetuses. Mice had free access to food and water and were housed under a 12-h light–dark cycle. For cell isolation, mice were deeply anesthetized with ketamine/xylazine injections and euthanatized via decapitation. All procedures in mice were performed under the approval of Veterinary Directorate of Prefecture of Heraklion (Crete) and carried out in compliance with Greek Government guidelines and the guidelines of FORTH ethics committee and were performed in accordance with approved protocols from the Federation of European Laboratory Animal Science Associations (FELASA) and Use of Laboratory animals. License number: EL91-BIOexp-02, Approval Code: 360667, Approval Date: 29/11/2021 (active for 3 years). Experiments with rat cells were carried out in Innoprot, following in-house protocols and procedures.

### Primary mouse astrocytes

Mixed glial cultures were isolated from the cortex of C57B/6 pups at post-natal day 2 (P2). Cells were plated in medium containing high-glucose DMEM, 200 U/mL penicillin, 200 µg/mL streptomycin and 10% fetal bovine serum (FBS). When cells reach 100% confluency (7–8 days), the anti-mitotic agent Ara-C was added in the media at a final concentration of 10 μM, for 3–4 days to target the highly proliferative microglial cells. Ara-C was removed and primary astrocytes (97% purity) were cultured at 5% CO_2_ and 37 °C.

### Primary mouse neural stem cell cultures

Primary adult hippocampal NSCs were isolated from mice at postnatal day 7 and cultured with DMEM/F-12 (Dulbecco’s Modified Eagle Medium/Nutrient Mixture F-12) medium (ThermoFischer Scientific, 11320033) containing 2% B27 wihtout Vitamin A (ThermoFischer Scientific, 12587010), 50 mg/mL Primocin (Invitrogen, ant-pm-1 500 mg), 20 ng/mL FGF (233-FB-025, R&D,), 20 ng/ml EGF (236-EG-200, R&D) and 50 mg/mL Heparin. For all the experiments cells were plated on PDL/laminin and the assays were performed after 24 h.

Primary Embryonic Cortical NSCs were isolated from E13.5 mice and cultured with DMEM/F-12 (Dulbecco’s Modified Eagle Medium/Nutrient Mixture F-12) medium (11320033, ThermoFischer Scientific) containing 2% B27 without Vitamin A (12587010, ThermoFischer Scientific), 50 mg/mL Primocin (ant-pm-1 500 mg, Invivogen), 20 ng/mL FGF (233-FB-025, R&D) and 20 ng/mL EGF (236-EG-200, R&D). For all the experiments cells were plated on PDL/laminin and the assays were performed after 24 h.

### Primary mouse hippocampal neurons

E17.5 mouse embryos were used to isolate primary hippocampal neurons, which were then grown in Neurobasal medium, containing 2% B27, 1% PenStrep, 1X GlutaMax, and 10 mM HEPES. Cells were kept at 37 °C and 5% CO_2_ in a humified incubator. Cells were cultured for 16–18 days before being exposed for 48 h to ENT-A011 (1 μM) and 5 μM oligomeric A-beta for the TUNEL assay. Following the manufacturer’s instructions, cells were subsequently fixed with 4% paraformaldehyde (PFA) and labelled with TUNEL (Roche, 11684795910). Cells were then immunostained (1:2000, Biolegend, 801201) against Tuj1 and imaged using confocal microscopy. Similarly, cells were cultured for 16–18 days before being treated for 4 h with ENT-A011 (1 μM) and 5 μM oligomeric A-beta for the synaptic plasticity assessment. Following that, cells were fixed with 4% PFA and immunostained against Tuj1 and Synaptophysin (1:1000, Invitrogen, PA1-1043). Images acquired using confocal microscopy and total area of synaptophysin positive puncta was measured and normalized to Tuj1 total area.

### Primary mouse cortical neurons

Primary cultures of cortical neurons were prepared from the cerebral cortices of Sprague–Dawley rat foetuses at embryonic day 18. Brains were removed and freed from the meninges, and the tissues were then dissected under a binocular microscope. Neurons were dispersed with trypsin 0.2% and DNase I 0.04% for 10 min at 37 °C, and plated on poly-l-lysine coated 96-well plates at a density number of 30.000 cells per well with neurobasal medium supplemented with B27 for 8 days at 37 °C in a humidified 5% CO_2_ atmosphere. At day 8, cells were pre-treated with BDNF (500 ng/mL) and ENT-A011 (1 μM) one hour before being subjected to a glutamate excitotoxicity condition (100 μM 15 min). After glutamate exposure, medium was replaced and cells were incubated for an additional 24 h for neurite outgrowth measurement.

### Human induced-pluripotent stem cells (hiPSCs)

hiPSC lines were kindly provided by Dr Z. Cader (MTA) and they were maintained in mTeSR (85850, STEMCELL Technologies, Vancouver, British Columbia, Canada) media on matrigel substrate (354277, Corning, New York, USA) and mechanically passaged using 0.5 mM EDTA (15575020, ThermoFischer Scientific, Rockford, USA). Differentiation was initiated with 10 μM SB431542 (616464, Sigma, Burlington, MA, USA) and 1 μM dorsomorphin (ab120843, Abcam, Cambridge, UK) following published protocols [57]. Further patterning was performed in N2B27 media with retinoic acid. Cells were plated at day 18 on laminin (L2020, Sigma, Burlington, MA, USA) coated 96 well plates for the Celltox and the MTT assays, 24well plates for the proliferation assay and 12well plates for the RNA sequencing.

### Immunoprecipitation and immunoblotting

Cells were plated at 70–80% confluency. Next day, they were deprived from serum for 16 h and subsequently treated with 500 ng/mL BDNF or 1 μM of compound ENT-A011 for 20 min. Cells were then lysed in Pierce™ IP Lysis Buffer (87788, ThermoFischer Scientific, Rockford, USA) containing proteases (539138, Calbiochem, Darmstadt, Germany) and phosphatases inhibitors (524629, Calbiochem, Darmstadt, Germany). Lysates were then immunoprecipitated overnight at 4 °C with TrkA antibody (1:100, 06–574, Sigma-Aldrich, St. Louis, MO, USA) or p75^NTR^ antibody (1:100, ab6172, Abcam, Cambridge, UK) followed by 4 h incubation with protein G-plus agarose beads (sc-2002, Santa Cruz Biotechnology, California, USA). Beads were then collected, washed 3X with lysis buffer, resuspended in SDS loading buffer and subjected to Western Blot against phosphorylated Tyrosine (1:1000, BAM1676, R&D systems, Mineapolis, USA) or Traf6 antibody (1:2000, ab33915, Abcam, Cambridge, UK). Whole cell lysates were subjected to Western Blot against TrkA (1:1000, 06-574, Sigma-Aldrich, St. Louis, MO, USA), phosphorylated TrkB (1:1000, ABN1381, Sigma-Aldrich, St. Louis, MO, USA), phosphorylated TrkC (1:1000, STJ90960, St John’s Laboratory, London, UK), TrkB (1:1000, 07-225-I, Sigma-Aldrich, St. Louis, MO, USA), TrkC (1:1000, C44H5, Cell Signaling Technology, Danvers, MA, USA), p75^NTR^ (1:1000, 839701, Biolegend, San Diego, USA), Traf6 (1:2000, ab33915, Abcam, Cambridge, UK) phosphorylated Akt (1:1000, 9721S, Cell Signaling Technology, Danvers, MA, USA) and total Akt (1:1000, 4691S Cell Signaling Technology, Danvers, MA, USA).

### Quantitative RT-PCR (QPCR)

For qPCRs in NSCs total RNA was extracted from cells using TRIzol Reagent (15596026, Thermo Fisher, Waltham, MA, USA), and cDNA was synthesized using the High-Capacity cDNA Reverse Transcription kit (4368814, Thermo Fisher, Waltham, MA, USA) according to the supplier protocols. For qPCR experiments run with SYBR green dye, for 20 s at 95 °C, followed by 40 cycles of 95 °C for 3 s and 60 °C for 30 s on a StepOne Real-Time PCR System (Thermo Fisher Scientific, Waltham, MA, USA. Β-Actin was used as a housekeeping gene to normalize the genes expression levels. Data were collected and analyzed using the StepOne Software v2.3 (Thermo Fischer Scientific, Waltham, MA, USA). Mouse primer sequences used are listed in Supplementary Table 1.

For qPCRs in astrocytes, total RNA was extracted with the Nucleospin RNA isolation kit (Macherey–Nagel, Dueren, Germany), according to the manufacturer’s instructions. cDNA synthesis was performed with the iScript cDNA synthesis kit (Bio-Rad, Hercules, CA, USA). qPCR was carried out using the SsoFast Eva Green Supermix (Bio-Rad Hercules, CA, USA), a CFX384 real-time System C1000 Thermal Cycler (Bio-Rad), and the Bio-Rad CFX Manager 3.1 software. The relative amount of mRNA was calculated with the ΔΔCt method, with 18 s used as a housekeeping gene.

### CellTox assay

CellTox assay (G8742, Promega, Leiden, Belgium) was used to assess survival of NIH-3T3 TrkB or PC12 cells under serum deprivation conditions, survival of primary hippocampal or embryonic cortical NSCs as well as human NPCs treated with 10 μM oligomeric Aβ1-42. Cells were plated in 96-well plates, NIH-3T3 TrkB cells were starved from serum for 24 h and subsequently treated with BDNF (500 ng/mL) or ENT-A011 (1 μM) in the presence or absence of TrkB inhibitor ANA-12 at 100 μM (SML0209, Sigma-Aldrich, Burlington, MA, USA) for 24 h. Cells were treated with 10 μM oligomeric Aβ1-42 and BDNF (500 ng/mL) or ENT-A011 (1 μM) for 24 h and the same conditions with ANA-12 (100 μM). CellTox assay reagents and Hoescht (1:10,000, H3570, Invitrogen, Massachusetts, USA) were added with the treatments and at the end of the 24 h treatments, cells were imaged with a Zeiss AXIO Vert A1 fluorescent microscope. CellTox positive cells were normalised to total number of cells for each image.

### MTT assay

Following Celltox assay for the primary mouse hippocampal or embryonic cortical NSCs and human NPCs, media was removed and the cells were washed with PBS before adding the MTT solution (M2128, Sigma-Aldrich, Burlington, MA, USA), final concentration 0.5 mg/mL for 4 h at 37 °C, 5% CO_2_. Supernatant was removed and DMSO-isopropanol solution (1:1 ratio) was added to the wells followed by incubation at room temperature for 15 min and at 4 °C for another 15 min. Absorbance was measured at 545 nm with a reference at 630 nm.

### Nomad biosensor assay

Transiently NOMAD/tGFP TrkB -HEK293 cells were plated in 96-well plate and were treated with ENT-A011 at increasing concentrations (100 nM, 300 nM, 1 μM, 3 μM, 10 μM and 300 μM) for a dose response curve. Fluorescence was measured in a plate reader at 485/528 (ex/em) for NOMAD/GFP and at 340/485 (ex/em) for Hoechst 33342. Data were normalized against negative control and nuclei count.

### LPS assay

Primary astrocytes were treated with the ENT-A011 (1 μM) and treatments were repeated after 24 h, LPS was added after 2 h at 100 ng/mL. Cells were lysed with Trizol after 4 h.

### Proliferation assay

Deprivation from EGF/FGF for 3 h was followed by treatment with EGF/FGF (20 ng/mL), BDNF (500 ng/mL) or ENT-A011 (1 μM) for 24 h for mouse primary NSCs. Cells were also treated with 10 μM oligomeric Aβ1-42 and BDNF (500 ng/mL) or ENT-A011 (1 μM) for 24 h. Human NPCs plated at day 25, at the following day cells were treated with BDNF or ENT-A011 (1 μM) and treatments were repeated after 24 h. BRDU was added to the cells 6 h prior the end of the treatment. Cells were fixed with 4% PFA and immunostained for BRDU (1:200, ThermoFischer Scientific B35128) and Nestin (1:1000, R&D NB100-1604) and imaged with a Zeiss AXIO Vert A1 fluorescent microscope.

### Cell differentiation assay

Primary mouse hippocampal NSCswere plated at confluency of 100,000 cells/mL. Following 3 days of plating, fresh medium was added to the cells without EGF/FGF, with or without BDNF (500 ng/mL) or ENT-A011 (1 μM). Medium changes were performed every other day and the cells were lysed with Trizol and samples were collected or fixed with 4% PFA after 10 days of culture.

### Isolation of RNA and 3′ RNA sequencing.

Human NPCs were plated at day 30 and treatments with BDNF (500 ng/mL) or ENT-A011 (1 μM) were performed every two days for a total of 10 days. At day 40, total RNA from biological triplicates was extracted using Trizol reagent (Thermo Scientific) as per the manufacturer’s protocol. Thus, a total of 3 replicates were performed for each treatment. The quantity and quality of extracted RNA samples were analyzed using RNA 6000 Nano kit on a bioanalyzer (Agilent). RNA samples with RNA integrity number (RIN) > 7 were used for library construction using the 3′ mRNA-Seq Library Prep Kit FWD for Illumina (QuantSeq-LEXOGEN) as per the manufacturer’s instructions. Amplification was controlled by qPCR for obtaining optimal unbiased libraries across samples by assessing the number of cycles (14) required for amplification of the library. DNA High Sensitivity Kit for bioanalyzer was used to assess the quantity and quality of libraries, according to the manufacturer’s instructions (Agilent). Libraries were multiplexed and sequenced on an Illumina Nextseq 500 at the genomics facility of IMBB FORTH according to the manufacturer’s instructions.

### Differential expression analysis (DEA) and gene ontology (GO) enrichment analysis

The quality of the FASTQ files was assessed with the FastQC software [58]. Reads were aligned to the human (hg38) genome with the Hisat2 aligner [59] (hisat2 -p 4 -q -x $reference_genome -U $read_file -S $outFile -no-spliced-alignment). Htseq-count [60] was utilized to summarize reads at the gene level (htseq-count $align_file $genes_gtf > $outFile). Differential expression analysis (DEA) was conducted by running EdgeR [61, 62] via SARTools 1.5.0 [63]. For each comparison, ENT-A011 and BDNF treated APOE4 human Cortical NPCs compared to untreated control cells, differentially expressed genes (DEGs, up and down-regulated) were defined by applying the following threshold *p*-adj < 0.1 and were considered statistically significant. Heatmaps were created in R with the Pheatmap R package. Differential expression analysis results are listed in Additional File 1. GO analysis was run on the webtool Metascape (http://metascape.org) [64]. Results are listed in Additional File 2 (upregulated) and Additional File 3 (downregulated) and representative GO terms enriched in upregulated targets were selected and summarized in Fig. 7.

### Preparation of Aβ oligomers

A-beta (1–42) peptide was obtained from AnaSpec (AS-20276, AnaSpec, California, USA) and prepared in accordance with the directions of the manufacturer. The peptide was diluted in DMEM at the appropriate concentration and left for 24 h at 37 °C. Following a 5' at 15,000 g centrifugation, oligomeric A-beta was derived from the supernatant and cells were treated.

### Neurite outgrowth

Rat cortical neurons were pre-treated for 1 h with BDNF (200 ng/mL) or the compound ENT-A011 (1 μM) 24 h before being subjected to a glutamate excitotoxicity condition where cells were incubated with 100 μM glutamate during 15 min in medium without B-27 component. After glutamate exposure, medium was replaced with neurobasal medium with B27 factor for additional 24 h.

Beta-III tubulin staining was determined by immunocytochemistry. Once cells were stained and image with fluorescent dyes, cells were washed with PBS and fixed with 4% PFA for 15 min. After the fixation step the samples were washed three times with PBS and permeabilized with PBS + 0.3% Triton for 10 min. The samples were then blocked with PBS and Bovine Serum Albumin (BSA) for 30 min and finally anti-tubulin III antibody were added at 1/1000 in PBS and 0.5% BSA for 60 min at room temperature. After three washing steps, the secondary antibodies Alexa 633 were added at 1/100 for 60 min to react against the primary antibody. The samples were then washed three times and measured in the Pathway 855 automated fluorescent microscope. Neurite average branch per neuron was measured in order to investigate the neurite outgrowth.

### Statistical analysis

Student’s t-test was utilized to compare two groups (treatment vs. control), to determine statistical significance. A significance threshold of *p* < 0.05 was applied to denote statistical significance. When examining fold change values, statistical analysis was conducted on log2-transformed data to ensure normal distribution.

### Biological and technical replicates

For human iPSC-derived neurons, different neuronal differentiations were carried out from independent cell lines (3 biological replicates), while additional technical replicates (minimum 3) were also performed for each condition in each line. For primary cell cultures, cells from independent passages were used (minimum of 3 biological replicates), with additional technical replicates (minimum 3) performed for each condition in each passage. The biological replicates are reported in each figure legend.

### ARRIVE checklist

All authors declare that the work has been reported in line with the ARRIVE guidelines 2.0.

## Results

### Design, synthesis and chemical studies of ENT-A011

The development of ENT-A011, as a novel DHEA derivative and BDNF mimetic with potential neurotrophic properties, was based on extensive molecular modeling studies using crystal structure data to predict the interactions of the compound with the TrkB receptor. Synthesis of ENT-A011, along with its analog ENT-A012, involved a multi-step process starting from DHEA, yielding the final compounds in high purity with confirmed stereochemistry. Complementary chemistry investigations provide additional insight on the pharmacological profile of ENT-A011, demonstrating weak-to-moderate inhibition of key human CYP450 enzymes and very slow depletion in human liver microsomes, indicative of metabolic stability. Extensive details regarding the methodology and in-depth chemistry studies can be found in the accompanying supplementary data.

### ENT-A011 activates TrkB and downstream signaling

**Fig. 1:**
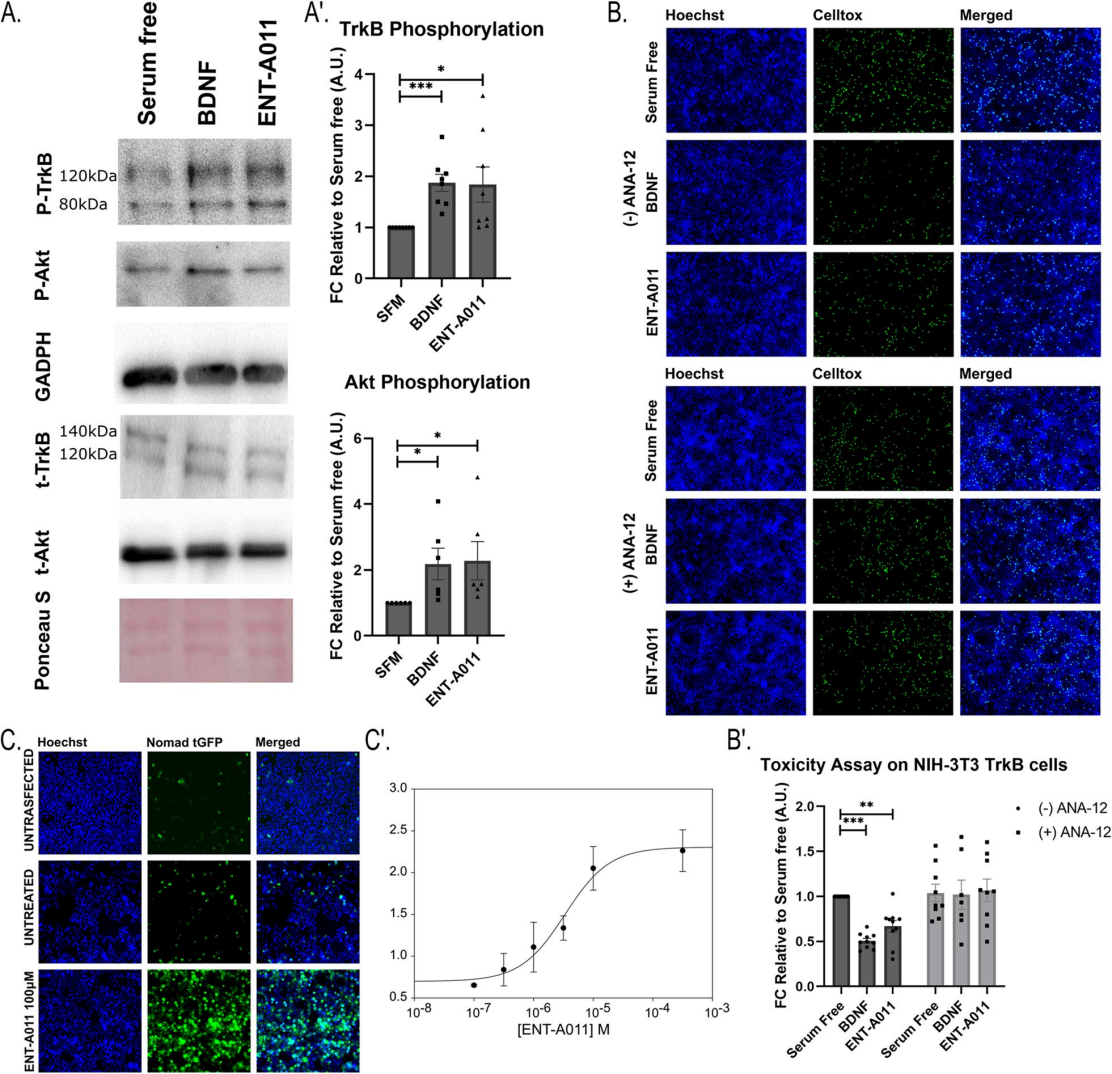
Screening of ENT-A011. ENT-A011 induces TrkB and its downstream target Akt phosphorylation after 20 min treatment in NIH-3T3 TrkB stable expressed cells. Representative blots (**A**) and quantification A’ of 8 (TrkB) and 6 (Akt) independent experiments are shown. Error bars represent S.E.M., Student’s t-test against Control; *p < 0.05; ***p < 0.001. **B**, B′ ENT-A011 protects NIH-3T3 TrkB cells from cell death caused by serum deprivation. Representative images (**B**) and quantification of Toxicity Assay (B′) on NIH-3T3 TrkB cells after treatment with BDNF or compound ENT-A011 for 24 h with or without TrkB inhibitor, ANA-12. (without ANA-12: BDNF N = 10/ENT-A011 N = 10, with ANA-12: BDNF N = 7/ENT-A011 N = 9), error bars represent S.E.M., Student’s t-test against Control; *p < 0.05, **p < 0.01, ***p < 0.001. **C**, C′ Nomad biosensor activation via TrkB phosphorylation in HEK 293 TrkB transiently transfected cells after ENT-A011 treatment. Receptor activation leads to increase in fluorescence levels. Representative images (**C**) and dose response curve for different compound concentrations (C′) are shown. Scalebars = 100 μm

To investigate the potential of new DHEA derivatives to mimic BDNF, we tested their capacity to activate the TrkB receptor and downstream signaling and their ability to prevent cell death in NIH-3T3-TrkB cells (Fig. 1A, B, Figure S5). First, using Western Blot analysis after a 20-min treatment with BDNF or compound (1 μM), we measured phosphorylated and total TrkB receptor isoforms and phosphorylated and total Akt. ENT-A011 exhibited a 1.84-fold change (FC) in TrkB phosphorylation, similar to the 1.87 FC observed with BDNF, and a 2.28 FC in Akt phosphorylation, compared to the 2.18 FC with BDNF (Fig. 1A).

Furthermore, ENT-A011 was also able to prevent cell death in the same cell system. NIH-3T3-TrkB cells, subjected to 24 h of serum deprivation, underwent further treatment with BDNF or ENT-A011 (1 μM) for another 24 h. ENT-A011 reduced cell death by 33% compared to the untreated control (FC in cell death levels equal to 0.67), without toxicity up to 100 μM (Figure S9). This effect was TrkB receptor-mediated, as confirmed by the selective TrkB inhibitor ANA-12, which negated the rescuing effect of both ENT-A011 and BDNF (Fig. 1B, B′). Notably, no protective effect was observed in naïve NIH-3T3 cells (lacking TrkB receptor) with BDNF or ENT-A011 treatment (Figure S10), affirming the receptor's role as a mediator of cell protection.

For further testing of ENT-A011, we employed a NOMAD biosensor cell line transfected with the TrkB receptor plasmid. Fluorescence increased proportionately with compound concentrations, with an IC_50_ calculated at 3.37 μM and no observed toxicity effects (Fig. 1C, C′).

### ENT-A011 increases TrkB and Akt phosphorylation, inducing Bdnf, Wnt, Creb and TrkB gene expression in primary mouse astrocytes

**Fig. 2:**
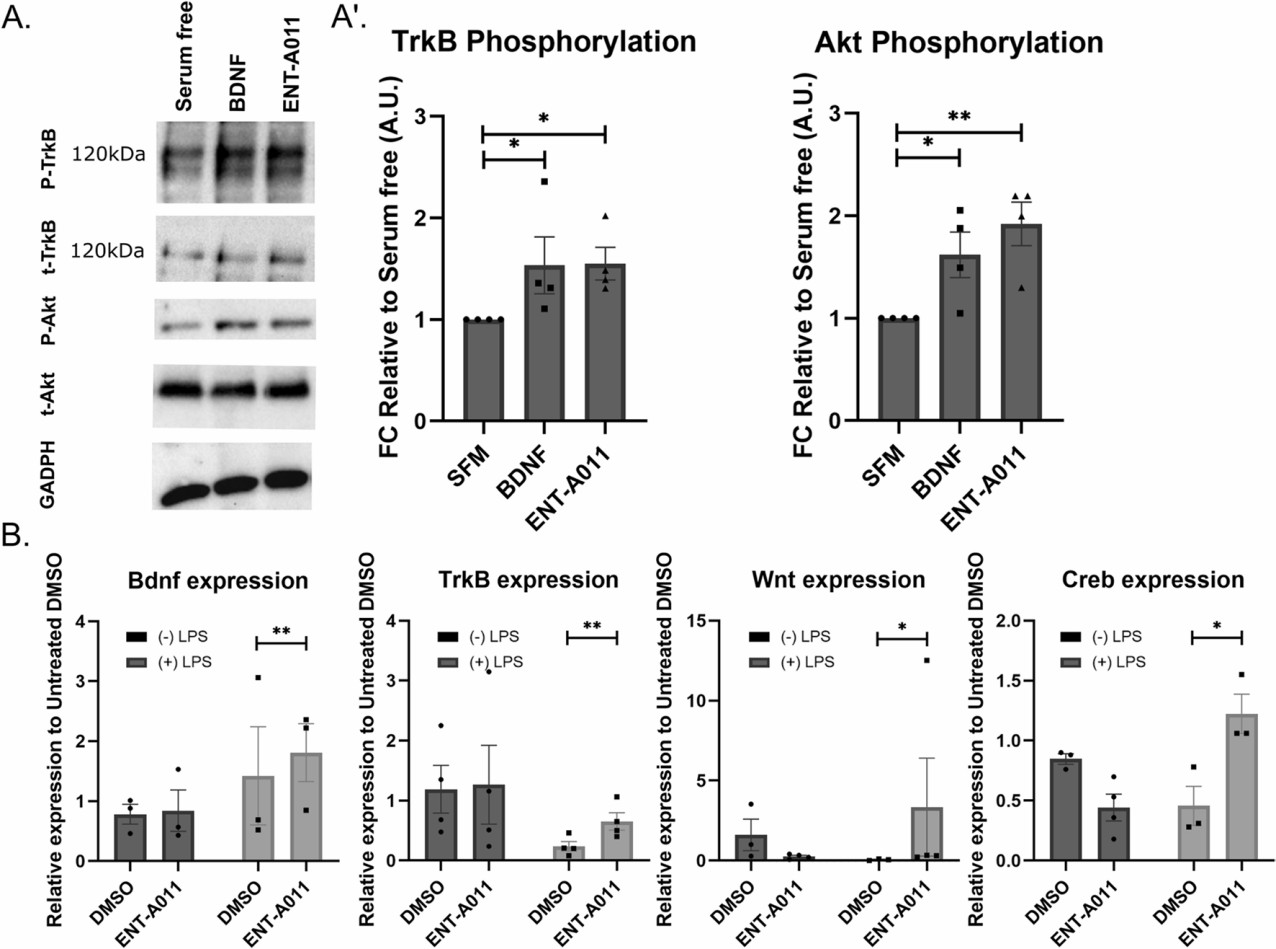
Effect of ENT-A011 on mouse astrocytes. The compound induces TrkB and its downstream target Akt phosphorylation after 20 min treatment in astrocytes. Representative blots (**A**) and quantification (A′) of 4 independent experiments are shown. Error bars represent S.E.M., Student’s t-test against Control; *p < 0.05; ***p < 0.001. **B** ENT-A011 increases Bdnf, Wnt, Creb and TrkB expression (detected by RT-qPCR) after LPS treatment in astrocytes. N = 3–4, error bars represent S.E.M., Student’s t-test against Control; *p < 0.05, **p < 0.01

Following results in the TrkB transfected cells, we then used primary mouse astrocytes (Figure S11), as an endogenously TrkB expressing population, to assess the effects of the compound in a primary cell line that is dependent upon TrkB signaling. ENT-A011 (1 μM) successfully increased levels of phosphorylation of the TrkB receptors as well as the downstream target Akt compared to the untreated control (Fig. 2A, A′), with comparable effect to BDNF. We also used RT-qPCR to assess the effect of TrkB activation after ENT-A011 treatment on the expression of key genes involved in the TrkB signaling pathway in astrocytes. In agreement with its ability to activate TrkB receptor, the compound led to an increase in the expression of *Bdnf*, *Wnt*, *Creb* and *TrkB* following astrocyte activation by Lipopolysaccharide (LPS) (Fig. 2B), thus untangling the effects of the pro-inflammatory LPS *vs* the ENT-A011-dependent effects.

### ENT-A011 promotes proliferation, survival and differentiation of adult primary mouse hippocampal neural stem cells

**Fig. 3:**
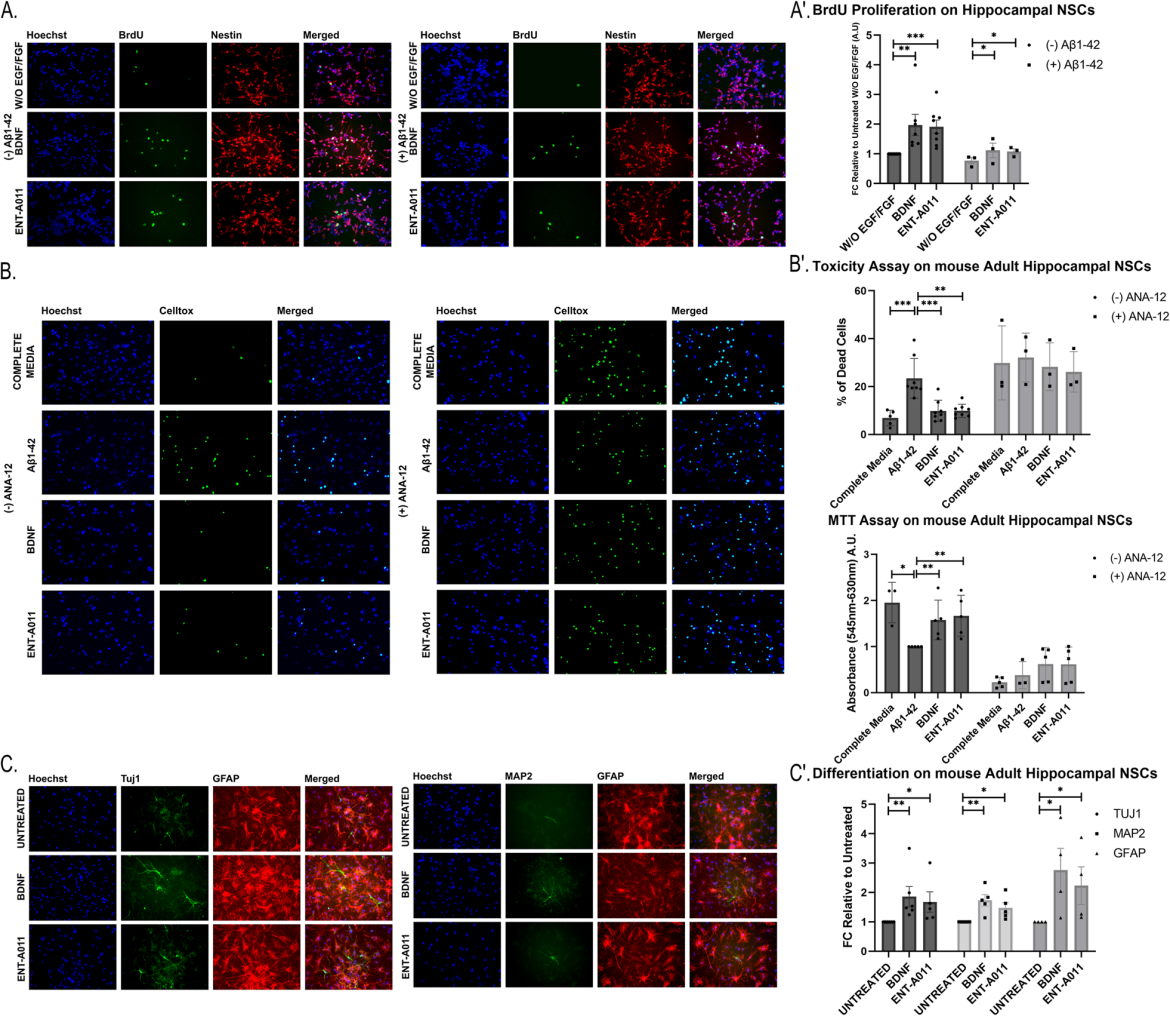
ENT-A011 induces neurogenesis on mouse adult hippocampal NSCs. **A**, A′ ENT-A011 increases proliferation in adult hippocampal NSCs. Representative fluorescence microscopy images of Immunostaining for BRDU and Nestin in adult hippocampal NSCs treated with BDNF or ENT-A011 for 24 h with or without Aβ42 (**A**). Quantification of BRDU fluorescence change with or without Aβ42 (A′). Quantification represents BRDU positive cells normalized against Nestin positive cells. (without Aβ42: N = 7–8, with Aβ42: N = 3), error bars represent S.E.M., Student’s t-test against Control; *p < 0.05, **p < 0.01, ***p < 0.001. B, B′. The compound rescues adult hippocampal NSCs from Aβ-induced cell death. Representative fluorescence microscopy images of Celltox cytotoxicity assay on hippocampal NSCs in the presence of Aβ42, treated with BDNF or ENT-A011, with or without the TrkB inhibitor ANA-12 for 24 h (**B**). Quantification of fluorescence change and MTT levels after BDNF or compound treatment, with or without ANA-12 N = 3–5. (B′). Quantification represents Celltox positive cells normalized against Hoechst positive cells (without ANA-12: N = 5–8, with ANA-12: N = 3)., error bars represent S.E.M., Student’s t-test against Control; *p < 0.05, **p < 0.01, ***p < 0.001. **C**, C′ ENT-A011 promotes differentiation of adult hippocampal NSCs. Representative fluorescence microscopy images of Immunostaining for Tuj1 and GFAP in adult hippocampal neural stem cells treated with BDNF or ENT-A011 for 10 days (**C**). Fold change in expression of *TUJ1* (N = 6 for untreated and BDNF, N = 5 for ENT-A011), *MAP2* (N = 5) and *GFAP* (N = 4) assessed by RT-qPCR in BDNF or ENT-A011 treated adult hippocampal NSCs relative to untreated control cells. (C′) Scalebars = 20 μm

The TrkB-mediated effects of ENT-A011 were confirmed in adult mouse hippocampal NSCs through proliferation, survival, and differentiation assays. ENT-A011 (1 μM) treatment significantly increased NSC proliferation rates, akin to BDNF (Fig. 3A, A’), with a fold change (FC) of 1.92 for ENT-A011 and 1.97 for BDNF compared to the untreated control.

We then assessed the effect of the compound on NSC survival after Aβ42 induced cytotoxicity. This system aims to mimic the conditions of the accumulation of oligomeric Aβ, as a major cause of neuronal cell death and reduced NSC proliferation in AD, especially in the hippocampus. Both BDNF and ENT-A011 (1 μM) countered the toxic effects of Aβ42, restoring proliferation levels (FC 1.08 for ENT-A011 and 1.12 for BDNF compared to the untreated control without Aβ42) and preventing apoptotic cell death (Fig. 3A, A′, B, B′). Mitochondrial activity, measured by the MTT assay, increased with ENT-A011 (FC 1.68) similar to BDNF (FC 1.58) compared to the Aβ42 control. The neuroprotective action of ENT-A011, like BDNF, was abolished in the presence of the TrkB inhibitor ANA-12 (Fig. 3B, B′).

In NSC differentiation, both BDNF and ENT-A011 (1 μM) promoted neuro- and gliogenesis, evidenced by immunostaining for TUJ1, MAP2, and GFAP markers after a 10-day culture without proliferating factors (EGF and FGF) (Fig. 3C, S12). RT-PCR (QPCR) analysis showed increased mRNA levels for neurogenic and gliogenic markers (Tuj1, Map2, Gfap) with ENT-A011 (FC 1.67, 1.48, 2.23, respectively) and BDNF (FC 1.86, 1.73, 2.76, respectively) treatment (Fig. 3C′).

### ENT-A011 promotes the proliferation and survival of embryonic primary mouse cortical neural stem cells

**Fig. 4:**
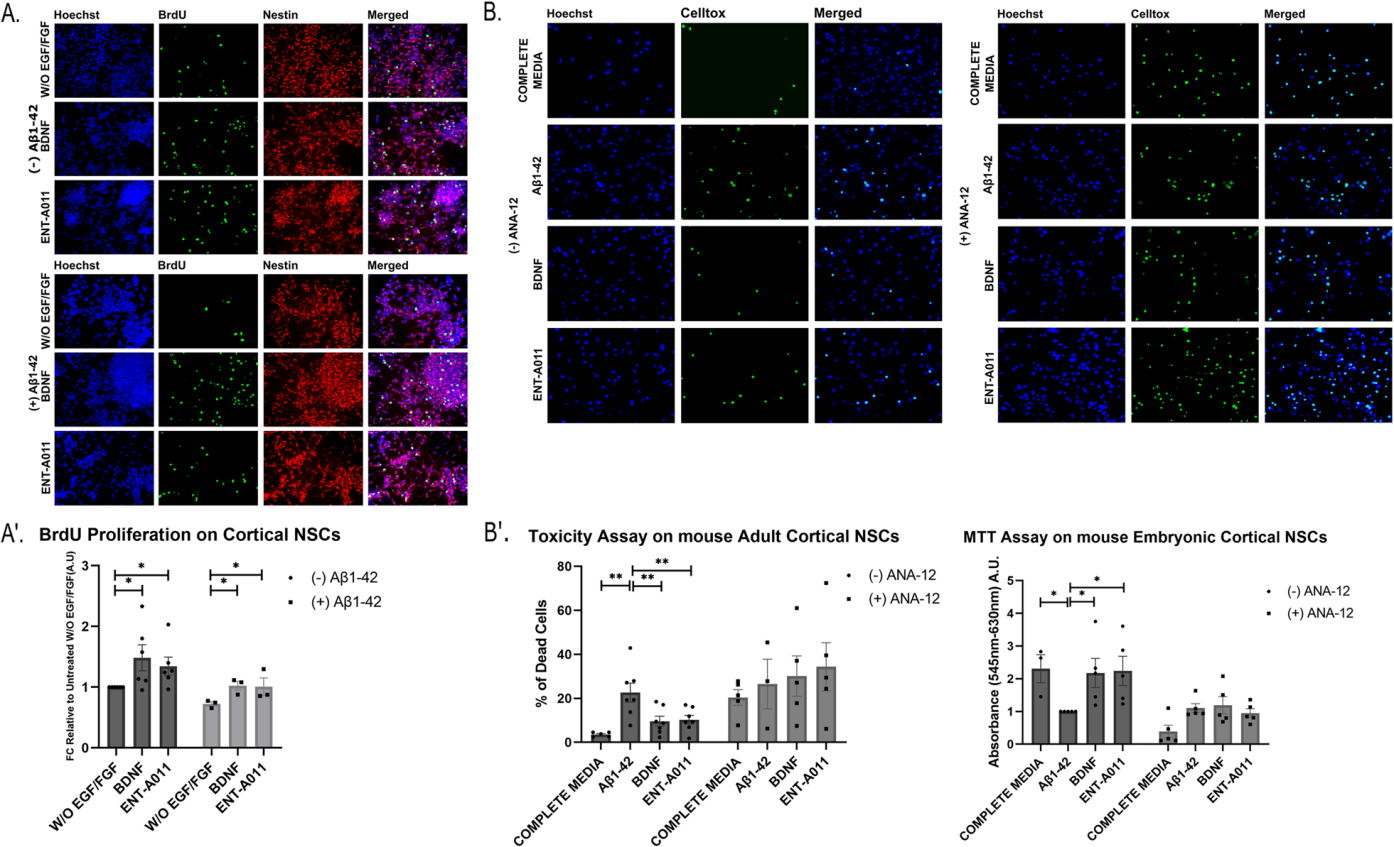
ENT-A011 has positive effect on mouse cortical NSCs. **A**, A′ ENT-A011 increases proliferation on cortical NSCs, Representative fluorescence microscopy images of Immunostaining for BRDU and Nestin in adult hippocampal NSCs treated with BDNF or ENT-A011 for 24 h with or without Aβ42 (**A**). Quantification of BRDU fluorescence change with or without Aβ42 (A′). Quantification represents BRDU positive cells normalized against Nestin positive cells. (without Aβ42: N = 6, with Aβ42: N = 3), error bars represent S.E.M., Student’s t-test against Control; *p < 0.05, **p < 0.01, ***p < 0.001. **B**, B′ The compound rescues cortical NSCs from Aβ-induced cell death. Representative fluorescence microscopy images of Celltox cytotoxicity assay on hippocampal NSCs in the presence of Aβ42, treated with BDNF or ENT-A011, with or without the TrkB inhibitor ANA-12 for 24 h (B′ right). Quantification of fluorescence change and MTT levels after BDNF or compound treatment, with or without ANA-12 (without ANA-12: N = 3–5, with ANA-12: N = 5) (B′ left). Quantification represents Celltox positive cells normalized against Hoechst positive cells. (without ANA-12: N = 5–7, with ANA-12: N = 3–5), error bars represent S.E.M., Student’s t-test against Control; *p < 0.05, **p < 0.01, ***p < 0.001. Scalebars = 20 μm

Based on the results in adult hippocampal NSCs, we tested the effect of the compound on the proliferation of embryonic mouse NSCs from the cortex. As shown in Fig. 4A, A′, ENT-A011 (1 μM) drove a significant increase in proliferation of mouse embryonic cortical NSCs (FC 1.34 compared to the untreated control), and counteracted the Aβ42-induced reduction of proliferation (increase from FC 0.72 to 1.01 after ENT-A011 treatment in presence of Aβ42 compared to the untreated control). Additionally, the compound reduced cell death levels similarly to BDNF (10.33% of total cells are apoptotic for ENT-A011 and 9.63% for BDNF compared to 22.6% in the untreated control) and promoted mitochondrial activity (FC in MTT absorbance of 2.24 for ENT-A011 and 2.18 for BDNF). Again, compound action of the compound in both assays was abolished in the presence of ANA-12, supporting it is mediated through TrkB signaling (Fig. 4B, B′).

### ENT-A011 protects mouse hippocampal neurons from Aβ42 cytotoxicity and promotes neurite outgrowth of mouse cortical neurons

In addition to its anti-amyloid activity in mouse hippocampal NSCs, ENT-A011 demonstrated efficacy in countering Aβ42-induced hippocampal neuron death and synapse loss. Oligomeric Aβ, a major contributor to neuronal trophic support reduction, can lead to neurotrophic signaling deregulation. Given that selective TrkB activation promotes neuronal survival, we explored the potential of ENT-A011 to mitigate Aβ's negative effects on cell death and synapse degeneration in neurons isolated from E17.5 mouse embryo hippocampi, a region severely impacted in AD.

**Fig. 5:**
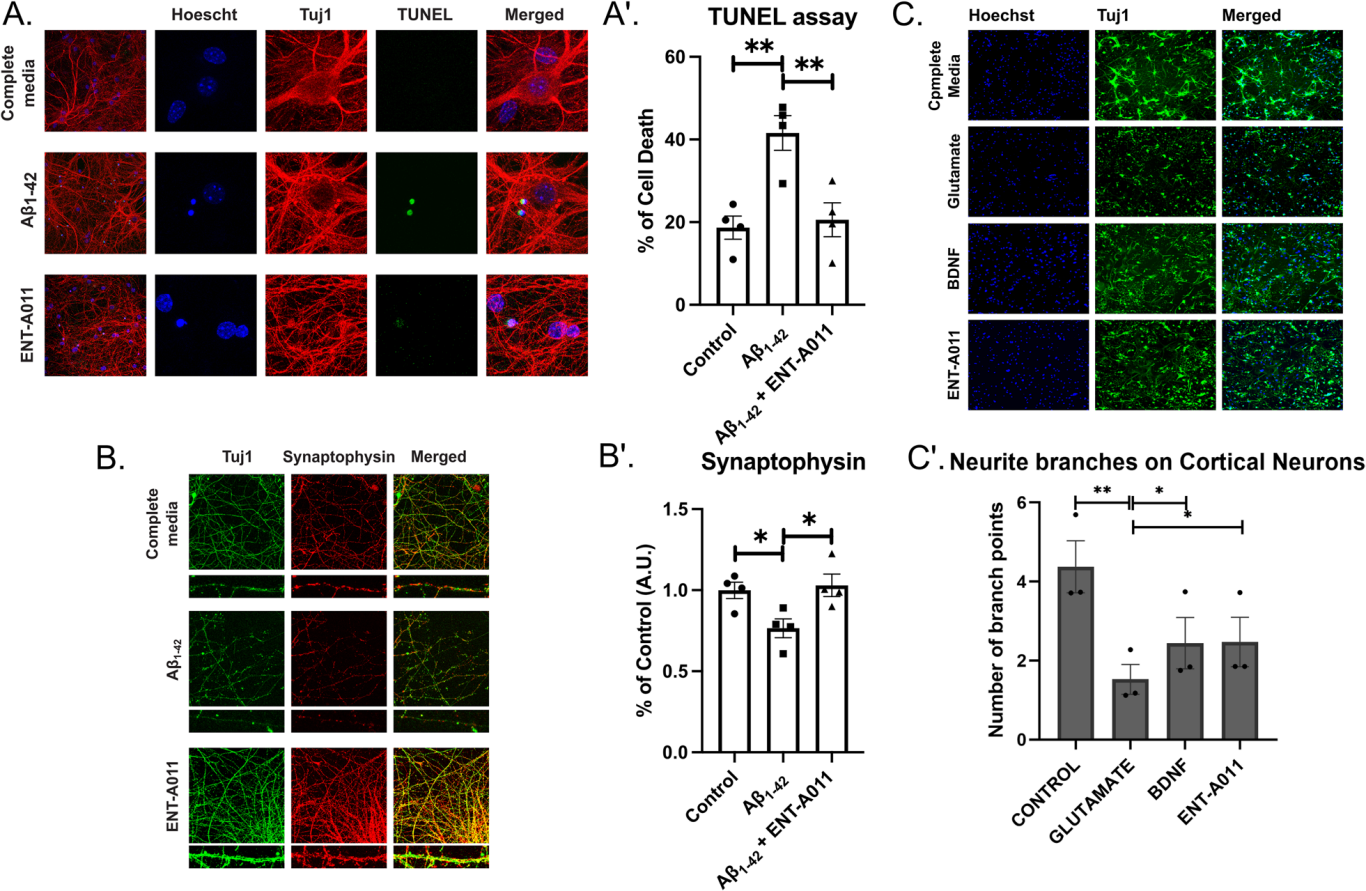
ENT-A011 protects mouse hippocampal neurons and rat cortical neurons against Aβ42 and glutamate toxicity respectively. **A**, A′, ENT-A011 reduces Aβ42 induced hippocampal neuron cell death. Primary hippocampal neurons were treated with Aβ42 for 48 h in presence of ENT-A011 and cell death was quantified using TUNEL assay. Representative images from 4 independent experiments. Data are shown as SEM. One-way ANOVA, Tukey’s Test correction: **p < 0.01; ***p < 0.001. **B**, B′ ENT-A011 protects against Aβ42-induced hippocampal synapse loss. Primary hippocampal neurons were treated with Aβ42 for 4 h in presence of ENT-A011, followed by staining against synaptophysin to assess synapse number. Total Synaptophysin area was normalized to total Tuj1 area in each image. Representative images from 4 independent experiments. Data are shown as SEM. One-way ANOVA, Tukey’s Test correction: ** *p* < 0.01. ENT-A011 reverts the decrease of neurite branches caused by glutamate on rat Cortical neurons. Representative fluorescence microscopy images of Immunostaining for Tuj1 in cortical neurons treated with BDNF or ENT-A011 for 1 h before glutamate treatment (C). Quantification of neurite branches (C′). N = 3, error bars represent S.E.M., Student’s t-test against Control; *p < 0.05, **p < 0.01, ***p < 0.001. White Scalebars = 20 μm, Yellow Scalebars = 5 μm

After a 48-h treatment with Aβ and ENT-A011 (1 μM), ENT-A011-treated cells exhibited significantly lower apoptosis (20.5% ± 4.1%) compared to Aβ-treated cells (41.5% ± 4.2%), akin to control cells (18.7% ± 2.8%) (Fig. 5A, A′). Testing ENT-A011 (1 μM) against Aβ-induced synapse degeneration in primary hippocampal neurons for 4 h revealed that the ENT-A011-treated group maintained a comparable number of synapses to the control group (FC 1.05 ± 0.06 of control), while the Aβ-treated group experienced a significant reduction in synapses (FC 0.78 ± 0.03 of control) (Fig. 5B, B′). These findings suggest that ENT-A011 protects primary mouse hippocampal neurons from oligomeric Aβ-induced cell death and synapse degeneration.

In the context of Aβ42 toxicity, we also investigated the impact of ENT-A011 on glutamate-induced excitotoxicity, a known event in AD neuropathology. Using primary rat cortical neurons, both BDNF and ENT-A011 (1 μM) promoted neurite outgrowth under glutamate excitotoxic conditions (Fig. 5C, C′), as evidenced by an increase in neurite branches per neuron (average of 2.44 branch points for BDNF and 2.47 for ENT-A011 compared to 1.53 for the control treated only with glutamate). Collectively, these results across hippocampal and cortical cell populations, encompassing both mature and stem cell origins, suggest that ENT-A011 holds significant therapeutic potential against key pathological conditions in AD.

### ENT-A011 increases the proliferation and survival from Aβ42-induced cell death of human neural progenitor cells, derived from healthy and AD-induced pluripotent stem cell lines

**Fig. 6:**
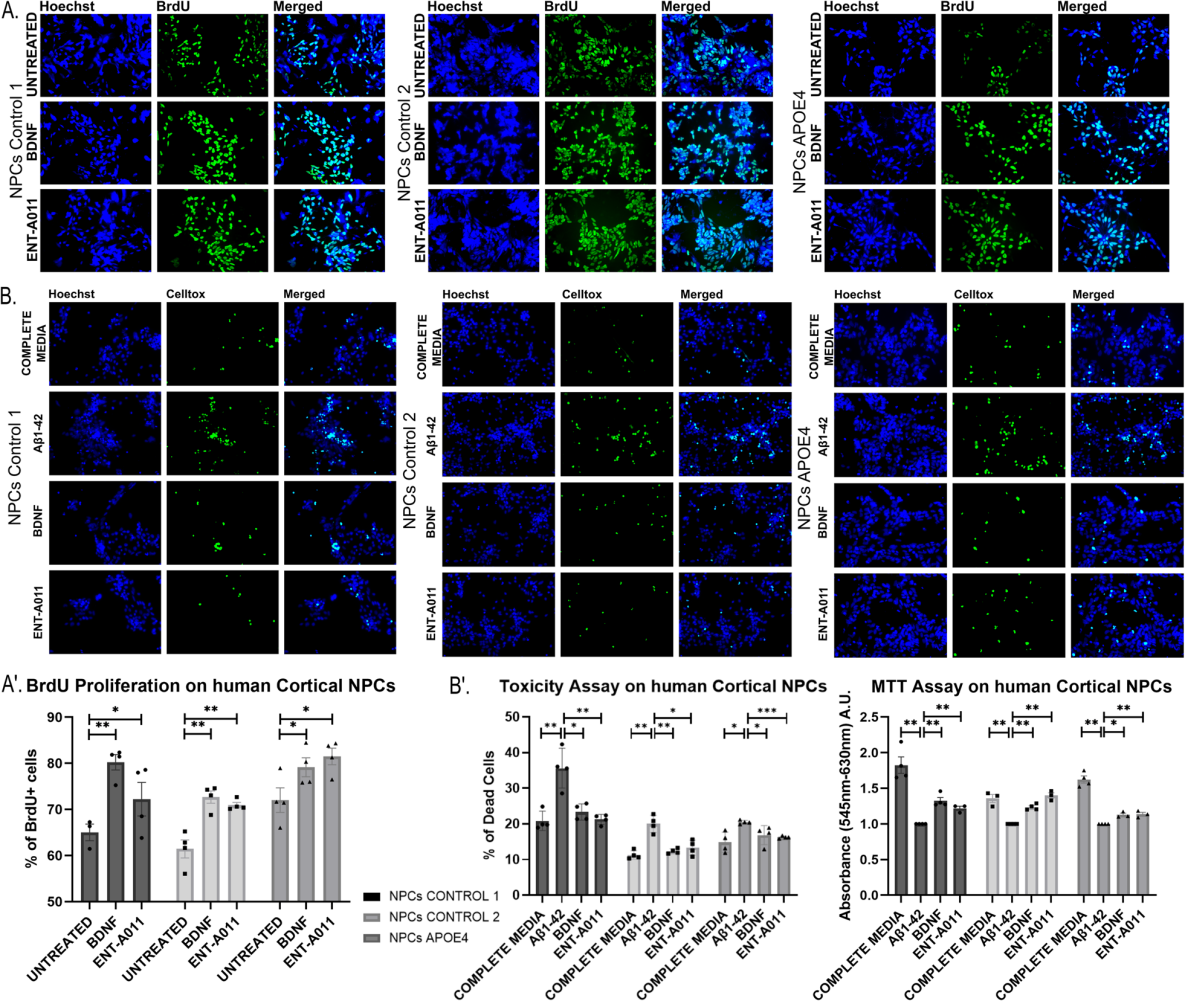
ENT-A011 effect on human cortical Neural Progenitor Cells (NPCs). Change in BRDU + cells (calculated as percentage of total Hoechst + cells) after BDNF or ENT-A011 treatment for 48 h in NPC derived from 3 human induced pluripotent stem cell (iPSC) lines. Representative fluorescence microscopy images of Immunostaining for BRDU and Hoechst (**A**) and quantification of BRDU fluorescence change (A′). **B**, B′ NPC derived from 3 human iPSC lines were treated with Aβ_1-42_ oligomers and BDNF or ENT-A011 for 48 h and compound effect on reducing toxicity was assessed via the Celltox cytotoxicity assay. Representative fluorescence microscopy images of Celltox cytotoxicity assay in the presence of Aβ42, treated with BDNF or ENT-A011 for 24 h (**B**). Quantification of fluorescence change and MTT levels after BDNF or compound treatment, with or without ANA-12 (B′). Quantification represents Celltox positive cells normalized against Hoechst positive cells. Three different lines were differentiated, N = 3–4, error bars represent S.E.M., Student’s t-test against Control; *p < 0.05, **p < 0.01, ***p < 0.001. Scalebars = 20 μm

The series of functional assays across diverse TrkB-expressing cell lines and neural stem cell populations from mice strongly supported the notion that ENT-A011 exhibits TrkB receptor-mediated activity comparable to BDNF, both in healthy and AD-simulating conditions. However, considering potential differences between mouse and human cell systems, we designed a human cell-based experimental setup to enhance the translational relevance of ENT-A011 for AD research. We used three different human iPSC lines—two from healthy individuals and one from an APOE4-AD individual—and differentiated these lines into cortical neuronal progenitor cells. Treatment of human NPCs at differentiation days 25–27 with ENT-A011 (1 μM) for 48 h led to increased proliferation (Fig. 6A, A′), demonstrated by BRDU incorporation (72.23% of total cells for ENT-A011 compared to 65.02% in untreated control for the first healthy line, 70.95% for ENT-A011 compared to 61.44% in the control for the second healthy line, and 81.47% for ENT-A011 compared to 72.01% in the control for the APOE4 line). Moreover, ENT-A011 treatment effectively counteracted Aβ42-induced toxicity in human NPCs, reducing Celltox assay cell death levels and increasing MTT assay mitochondrial activity, mirroring the effects of BDNF (Fig. 6B, B′).

**Fig. 7:**
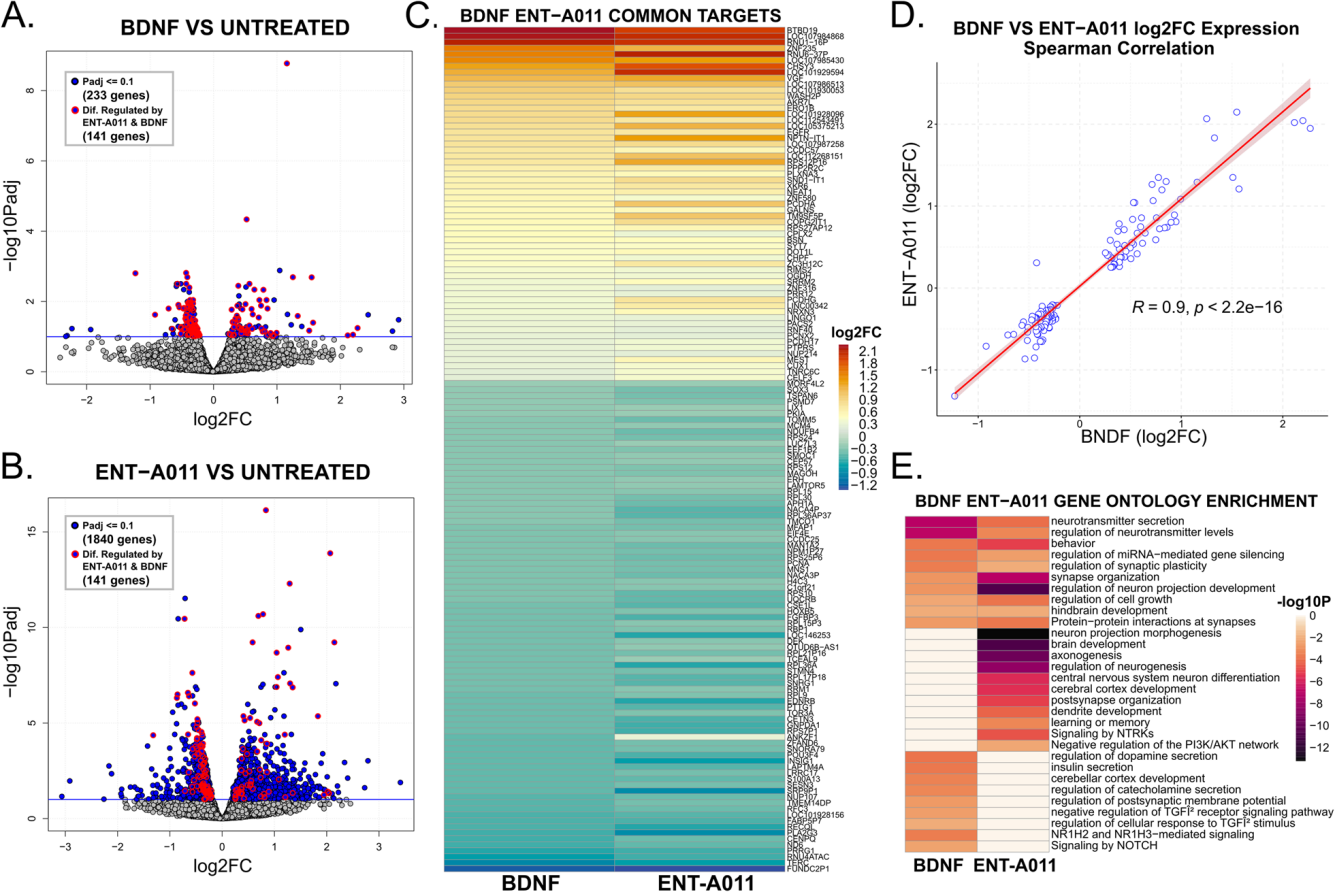
Differential expression analysis of ENT-A011 and BDNF treated APOE4 human Cortical Neural Progenitor Cells (NSCs) compared to untreated control cells. **a** Volcano plot of differentially regulated genes after BDNF treatment compared to untreated control cells. Negative log10 adjusted *p* values (y axis) are plotted against log2 fold change in expression for each gene. Genes with Padj lower or equal to 0.1 (blue horizontal line) are highlighted in bold blue colour and genes that are also differentially regulated by ENT-A011 (*p* adj ≤ 0.1) are highlighted with red circles. **b** Volcano plot of differentially regulated genes after ENT-A011 treatment compared to untreated control cells. Negative log10 adjusted *p* values (y axis) are plotted against log2 fold change in expression for each gene. Genes with Padj lower or equal to 0.1 (blue horizontal line) are highlighted in bold blue colour and genes that are also differentially regulated by ENT-A011 (*p* adj ≤ 0.1) are highlighted with red circles. **c** Heatmap of log2 Fold Change in expression for genes differentially regulated by both BDNF and ENT-A011, ranked by decreasing log2FC values in BDNF treated cells. **d** Scatter plots of log2 fold change in gene expression after BDNF (x axis) or ENT-A011 (y axis) treatment. Spearman correlation coefficient (0.9) and *p* value (2.2e^−16^) are displayed next to line of best fit. **e** Heatmap of negative log10 *p* values for gene ontology enrichment analysis of upregulated genes (*p* adj ≤ 0.1) after BDNF (left column) or ENT-A011 treatment. Unique terms to either treatment group are plotted in pale orange in the other group

To obtain a quantitative and global assessment of the gene networks underlying ENT-A011 action and if they are comparable to BDNF, we conducted 3’ quant mRNA sequencing on human APOE4 iPSC-derived NPCs (day 27) treated with BDNF, ENT-A011 (1 μM), or untreated controls. Differential expression analysis identified a core network of 141 genes regulated similarly by both ENT-A011 and BDNF, comprising nearly 60% of all genes differentially regulated by BDNF (233 genes, Fig. 7a). Interestingly, ENT-A011 treatment induced differential expression in 7.9-fold more target genes (1840) than BDNF (Fig. 7b). Examining the commonly regulated genes revealed a highly reproducible pattern in expression changes after ENT-A011 and BDNF treatment (Fig. 7C), with a significant positive correlation between their effects on the expression of shared target genes (Spearman Correlation coefficient 0.9, *p* value 2.2e−16, Fig. 7D). Genes upregulated by both treatments were associated with shared enriched ontology terms related to neurotransmitter secretion, synapse organization, and regulation of cell growth (Fig. 7e). Additionally, genes exclusively upregulated by ENT-A011 were linked to processes related to neuronal development and function, such as cerebral cortex development, regulation of neurogenesis, and signaling by neurotrophic receptors (NTRKs) (Fig. 7e). Shared downregulated targets were associated with terms related to cell cycle and mitosis control, translational and transcriptional regulation, and ribosomal biogenesis (259/349 BDNF-enriched terms were also enriched in ENT-A011 downregulated genes).

## Discussion

BDNF is the most widely distributed neurotrophic growth factor in the central nervous system (CNS), with crucial roles for CNS growth and development. BDNF has been shown to promote neurogenesis, both in the embryonic and adult brain, and is essential for neural stem cell proliferation, differentiation and survival [22, 27, 65, 66]. In recent years, compelling evidence has suggested neurogenesis impairment in Alzheimer’s disease to be linked to decreased levels of the BDNF in the hippocampus and the cortex [32]. Consequently, various studies have investigated the therapeutic potency of BDNF in neurodegenerative diseases and especially in AD [22, 35]. However, its inability to penetrate the blood–brain barrier, minimal diffusion in tissues and sensitivity to proteolysis hinder the potential of BDNF as a drug candidate for AD. Aiming to overcome these shortcomings and based on our previous studies on steroidal neurotrophin mimetics, we designed and synthesized compound ENT-A011 which shared the beneficial functions of BDNF in TrkB dependent cell lines and neural cell populations, but not its limitations. Importantly, our experiments show that this novel compound has neurogenic and neuroprotective properties, not only in mouse neuronal cell populations, but most importantly in human NPCs derived from healthy and AD donors.

ENT-A011 induces phosphorylation of TrkB, but not TrkA or TrkC receptors while it does not activate the pan-neurotrophin p75^NTR^ receptor pathway (Figure S8). Molecular modeling suggested two possible binding sites for ENT-A011 at the interface of the extracellular TrkB domain with the neurotrophin. ENT-A011 also activates TrkB receptor downstream signaling targets, increasing Akt phosphorylation, a kinase pathway associated with cell survival. Indeed, the compound reduces cell death levels after serum deprivation in survival assays in NIH-3T3 TrkB cells, stably transfected with TrkB, an effect abolished after treatment with TrkB inhibitor ANA-12 or in naïve NIH-3T3 cells, providing further evidence that the compound specifically protects cells through TrkB receptor activation. In this first round of screening in TrkB-expressing cell lines, we also show dose-dependent action of the compound by using a nomad biosensor cell system produced by Innoprot. Having confirmed that ENT-A011 can activate the TrkB receptor and the downstream pathway and its functional role in survival, we then investigated the actions of the compound on mouse primary astrocytes. These cells naturally express TrkB expressing population, playing an important role in neurogenesis. The interaction of astrocytes with NSCs supports proliferation, differentiation and synapse formation of the latter, acting as a major source of BDNF [26, 38, 67–70]. ENT-A011 showed the ability to induce phosphorylation of the full length TrkB receptor isoform in primary mouse astrocytes, and it was also able to induce *Creb, Wnt* and *Bdnf* expression after LPS-induced astrocyte activation [71]. LPS leads to reduction in cell proliferation and the production of new neurons [72, 73]. It is of note that the levels of hippocampal BDNF and phospho-TrkB were shown to decrease after astrocyte activation in LPS treated mice [74].

Activation of *Creb* in astrocytes is involved in proliferation, survival, maturation and development of neural stem and progenitor cells and correlates with the expression and activation of BDNF [75, 76]. In addition, the *Wnt* family is expressed by astrocytes and Wnt receptors are present in NSCs. Wnt signaling has been shown to hold roles in enhancing proliferation, the induction of neuronal differentiation, fate-commitment, development and maturation of NSCs, progenitor cells and newborn neurons [67, 77, 78]. It is also worth noticing that astrocytes express both the TrkB full length receptor and the TrkB truncated form of the receptor depending upon their maturation stage, with both types of the receptor been activated by BDNF, either by phosphorylation of the tyrosine residue for the full-length isoform or through calcium release for the truncated form [79–81]. Therefore, our results confirm the ability of ENT-A011 to activate the TrkB pathway in a naturally expressing TrkB cell population and provide evidence on its effect on the expression of genes that are associated with astrocyte neurogenic and its neuroprotective action.

Alzheimer’s disease is characterized by the accumulation of toxic oligomeric amyloid-β and neuronal loss in hippocampus and cortex. Oligomeric Amyloid-β, especially Aβ42, reduces neuronal proliferation and differentiation, an event associated with reduced hippocampal neurogenesis. Additionally, Aβ42 induces apoptosis at a concentration of 10 μM in NPCs [82, 83]. Aβ affects neurogenesis in mouse and human models of Alzheimer’s disease [84–86]. An increasing number of studies suggest that induction of adult neurogenesis before the progression of AD may be a potential therapeutic or prevention strategy earlier in life [36, 87]. We thus examined the effect of ENT-A011 on the proliferation and survival of mouse hippocampal and cortical NSCs, in the absence or the presence of Aβ42, compared to the activity of BDNF. ENT-A011 was able to significantly reduce levels of cell death in survival assays and countered the decline of cell proliferation after Aβ42 treatment. Moreover, ENT-A011 induced mouse hippocampal neural stem cell differentiation towards neurons and astrocytes. These findings considered together, strongly support the ability of the compound to promote neurogenesis mimicking BDNF. It is well established that Amyloid-β also impairs NSC viability by disrupting mitochondrial function [88]. Interestingly, ENT-A011 treatment reversed the reduction in metabolic activity caused by Aβ42 treatment, in a manner comparable to BDNF. Finally, it is interesting to note that the in vitro assays used here highlight the potential of ENT-A011 in promoting neurogenesis in the heterogeneous NSC pools isolated from mouse adult or embryonic brains, but we hypothesize similar effects would be expected across different NSC populations, such as quiescent NSCs. NSC quiescence has been shown as a key mechanism involved in maintaining the adult neurogenic niche long-term and quiescent NSCs have been suggested as a putative drug target [23, 24, 89]. Therefore, future in vivo work could investigate this as a target of ENT-A011.

Access to human induced pluripotent stem cells in latest years has offered the opportunity to study neurogenesis in a human system. Such new translational platforms for human disease, open a unique channel for studying the effects of BDNF on human NSCs and neurons and provide a system to screen potential neurogenic drugs for neurodegenerative diseases and especially AD, while recent work has even highlighted the potential of iPSC-derived NPCs and neurons for studying the connection of adult neurogenesis and Alzheimer disease [17]. Previous work has shown that BDNF induces the proliferation of human NPCs from both control and AD iPSC lines [90]. In the present study, we show that ENT-A011, as a synthetic BDNF mimetic, has the ability to promote proliferation and mitochondrial activity, as well as decrease Aβ42 induced cell death in NPCs derived from human iPSCs from healthy controls and an APOE4 cell line. Interestingly, BDNF was found decreased in human APOE4 carriers [91], and hippocampal progenitor cell proliferation was decreased in mice with the human APOE4 [92, 93], while NPCs from APOE4 human iPSC-derived neurons showed impaired capacity for proliferation [94]. We thus compared the effects of BDNF and ENT-A011 treatment on human NPCs derived from iPSCs from the APOE4 line, comparing their actions on target gene network activation, using RNA sequencing. Interestingly, the RNA-seq data showed low levels of BDNF in our APOE4 cells lines, in agreement with previous reports, but comparably high levels of TrkB (Supplementary Table 2), providing support on the merit of BDNF treatment as a therapeutic avenue. The connection between APOE4 and decreased BDNF has also been shown at the protein level before, both in the serum [95] and hippocampus [96] of human APOE4 subjects, while APOE4 treatment (but not APOE2 and APOE3) has been shown to lead to decreased BDNF secretion in human astrocytes [96]. This action of APOE4 has been shown to be enacted through the nuclear translocation of histone deacetylases (HDACs) in human neurons, with HDAC6 specifically interacting with BDNF promoter IV [97]. Differential expression analysis showed that ENT-A011 activates a downstream gene network largely overlapping with the downstream network activated by BDNF. Indeed, nearly 60% gene targets were upregulated and downregulated by the compound in a similar fashion as BDNF. Upregulated targets after ENT-A011 treatment are associated with regulation of cell growth, axonogenesis, regulation of neurogenesis and signaling by NTRKs, providing valuable support of the action of ENT-A011 in human neuronal progenitors. At the same time, both BDNF and the compound led to downregulation of a large number of targets involved in key cellular processes related to control of mitosis and the regulation of transcription and translation. The downregulation of cell cycle regulators is in line with the promotion of differentiation in treated cells. Of particular interest are genes like cyclin and related proteins that are associated with G1 to S phase transition, as G1 phase lengthening has been previously suggested as a key event in the initiation of neurogenesis [98–100]. Specifically, increase in Cyclin Dependent Kinase 4 (*Cdk4*), which is downregulated by ENT-A011, or Cyclin Dependent Kinase 2 (*Cdk2*), downregulated by BDNF, leads to inhibition of neurogenesis by preventing G1 lengthening and comparably loss of *Cdk2* and *Cdk4* has been shown to promote neural stem cell differentiation [101, 102]. Finally, the suppression of ribosomal synthesis pathways is also in accordance to an increase in differentiation, as the decrease of ribosome biogenesis is another hallmark that has been linked to differentiation and early neurogenesis [103–105]. Overall, the upregulated and downregulated processes highlight that ENT-A011, at 10 days of treatment, is activating neural differentiation networks in human NPCs with effect even more profound than BDNF, at the concentrations used here. Coupled with its ability to counter neurotoxicity and promote NPC proliferation at shorter treatments (24–48 h), these data underline the promising potential of the compound for further investigation as a drug candidate. Finally, while the specificity of the compound for TrkB among neurotrophin receptors was demonstrated, it should be noted that the 7.9-fold difference in differentially regulated targets compared to BDNF may indicate that other downstream signaling pathways are also induced.

Genes co-upregulated by BDNF and ENT-A011 include various targets of interest associated with neural development and function, as well as neuronal disorders and AD pathology. Examples include complexin 2 (*CPX-2*) that modulates activity-induced BDNF release in hippocampal neurons [106] and cut like homeobox 1 (*CUX1*) regulates spine development, dendritic branching and synaptogenesis in cortical neurons [107]. A co-upregulated target of particular interest is also presynaptic organizer bassoon (*BSN*), as not only do BSN mutant mice have increased BDNF expression, but this leads to hippocampal enlargement because of increased proliferation and reduced apoptosis of new neurons in the dentate gyrus [108]. Additionally, specific upregulated targets of ENT-A011 provide further genes of interest, including t-box brain transcription factor 1 (*TBR1*), a regulator of neural stem cell differentiation that is expressed during cortical development (a marker of cortical deep layer neurons according to the protocol used for differentiation of iPSCs towards cortical neurons) and in the adult hippocampus [109, 110]. Moreover, DMRT Like Family A2 (*DMRTA2*) regulates NPC maintenance during cortical development and is necessary for early cortical neurogenesis in mice [111, 112], while protein Tyrosine Phosphatase Receptor Type D (*PTPRD*) is a regulator of developmental neurogenesis through TrkB [113]. A striking target of ENT-A011 is also amyloid Beta Precursor Like Protein 1 (*APLP1*), a member of the amyloid precursor protein family involved in brain development that is directly cleaved by γ-secretase, but whose derivative ALP-1 peptides are not toxic to neuronal cells, unlike APP derived Aβ [114, 115]. Finally, ENT-A011 upregulated targets also include BDNF/TrkB signaling members, such as docking protein 5 (*DOK5*) that act as a substrate of *TrkB* [116], NCK Adaptor Protein 2 (*NCK2),* whose interaction with TrkB has been shown to be promoted by BDNF in cortical neurons [117] and BCL11 Transcription Factor B (*BCL11B*) that acts as an upstream regulator of multiple other BDNF signaling genes [118]. Finally, several enriched processes after either treatment indicate an effect of BDNF and ENT-A011 on neuronal function (Supplementary Data).

## Conclusions

In this study we performed a broad characterization of the ability of a new candidate therapeutic, with desirable pharmacological properties, to promote neurogenesis and neuroprotection using a complement of adult and embryonic stem cell models. By combining relevant mouse cell lines with human iPSC derived NPCs, we strived to create a robust in-vitro testing platform with translational value for neurogenic drug development, in line with promoting the 3R principles of replacing, reducing and refining animal use in research. This work presents a novel synthetic TrkB agonist, ENT-A011, that exhibits high neurogenic and neuroprotective action on par with the endogenous ligand BDNF, while having advantageous drug-like properties for putative therapeutic applications that includes its small size, a lipophilic nature and appropriate metabolic profile.

### Supplementary Information

Additional file 1. 

Additional file 2. 

Additional file 3. 

Additional file 4. 

Additional file 5. 

## Data Availability

The iPSC RNA-seq dataset supporting the conclusions of this article is available in the European Nucleotide Archive (ENA) repository under study accession PRJEB62550.

## Abbreviations

NSC: Neural stem cell
AD: Alzheimer’s disease
BDNF: Brain-derived neurotrophic factor
TrkB: Tyrosine receptor kinase B
DHEA: Dehydroepiandrosterone
Aβ: Amyloid-β
NPCs: Neural progenitor cells
SVZ: Subventricular zone
AHN: Adult hippocampal neurogenesis
CNS: Central nervous system
BBB: Blood–brain barrier
iPSC: Induced pluripotent stem cell
RNA-seq: RNA sequencing
HRMS: High-resolution mass spectra
TLC: Thin-layer chromatography
HPLC: High-performance liquid chromatography
SP: Standard precision
IFD: Induced fit docking
FELASA: Federation of European Laboratory Animal Science Associations
FBS: Fetal bovine serum
PFA: Paraformaldehyde
BSA: Bovine serum albumin
TBSCl: Tert-butyldimethylsilyl chloride
DMP: Dess–Martin periodinane
DCM: Dichloromethane
FCC: Flash column chromatography
FC: Fold change
LPS: Lipopolysaccharide
DEA: Differential expression analysis
CPX-2: Complexin 2
CUX1: Cut like homeobox 1
BSN: Presynaptic organizer bassoon
TBR1: T-box brain transcription factor 1
DMRTA2: DMRT like family A2
PTPRD: Tyrosine phosphatase receptor type D
APLP1: Amyloid beta precursor like protein 1
DOK5: Docking protein 5
NCK2: NCK adaptor protein 2
BCL11B: BCL11 transcription factor B
