# Supplementary material for: Comprehensive characterization of the neurogenic and neuroprotective action of a novel TrkB agonist using mouse and human stem cell models of Alzheimer’s disease
Source: Stem Cell Res Ther. 2024 Jul 6;15:200. doi: 10.1186/s13287-024-03818-w (PMC11227723; doi:10.1186/s13287-024-03818-w)

Figure 1A_WB1

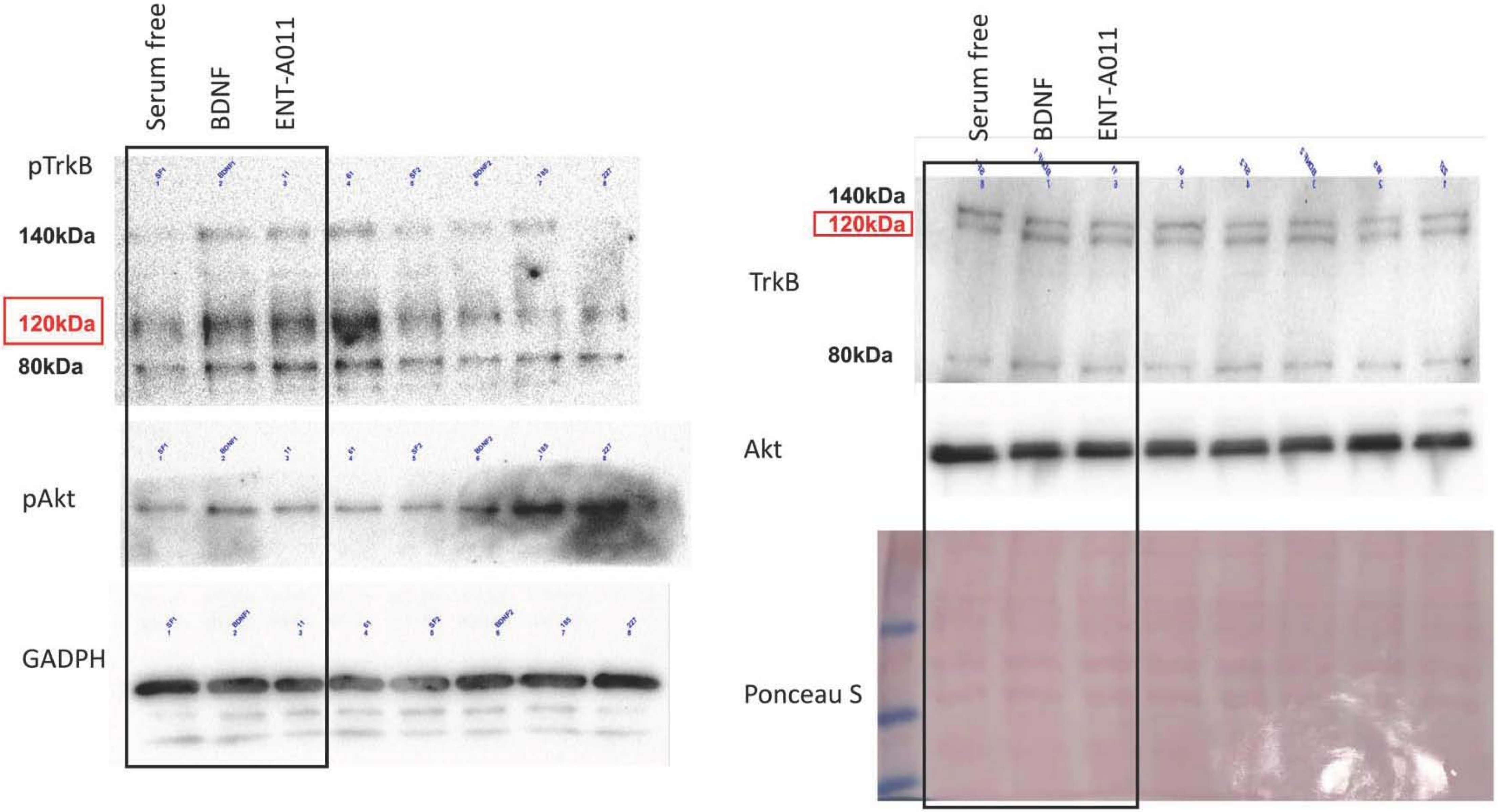

Figure 1A_WB2

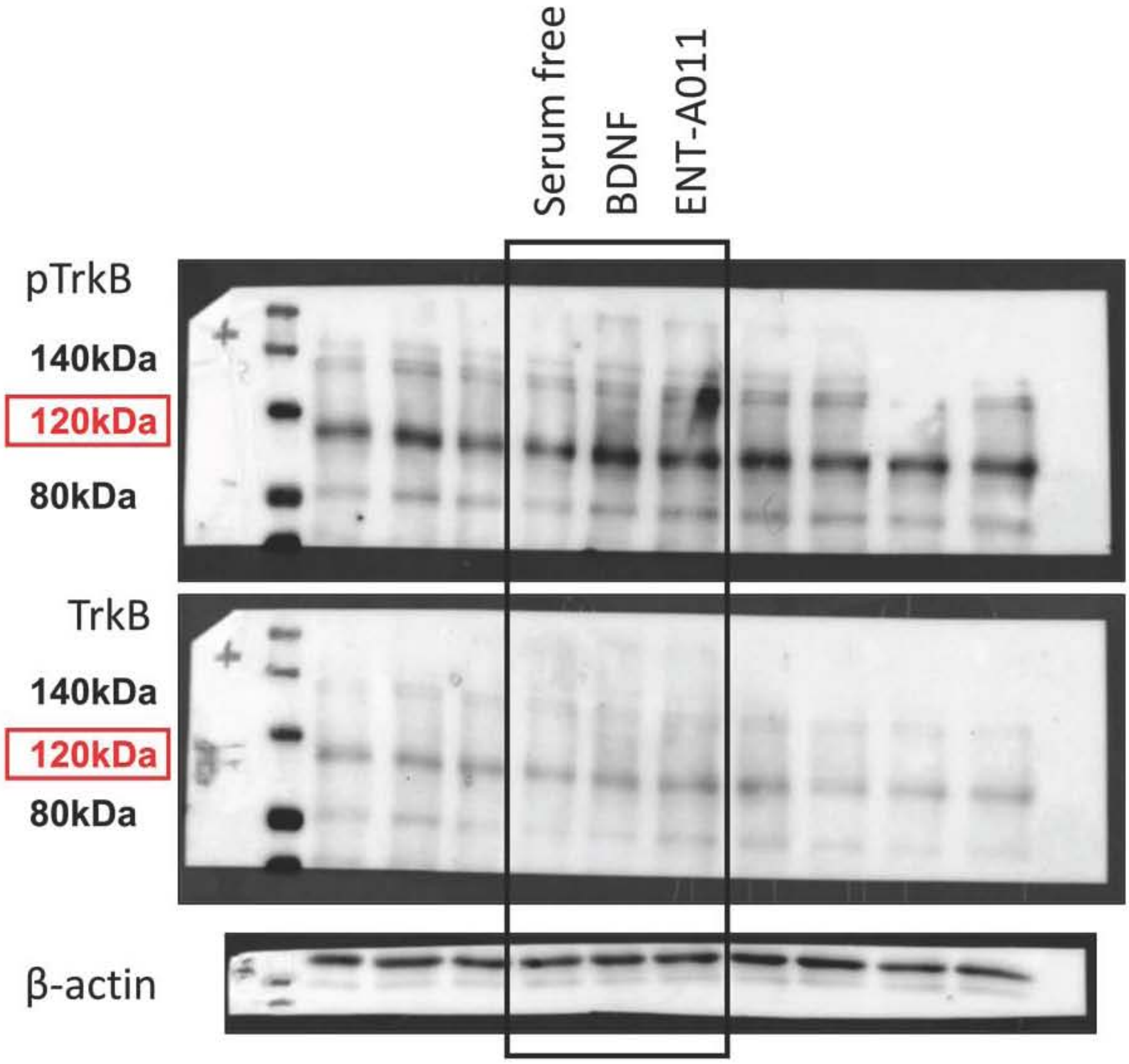

Figure 1A_WB3

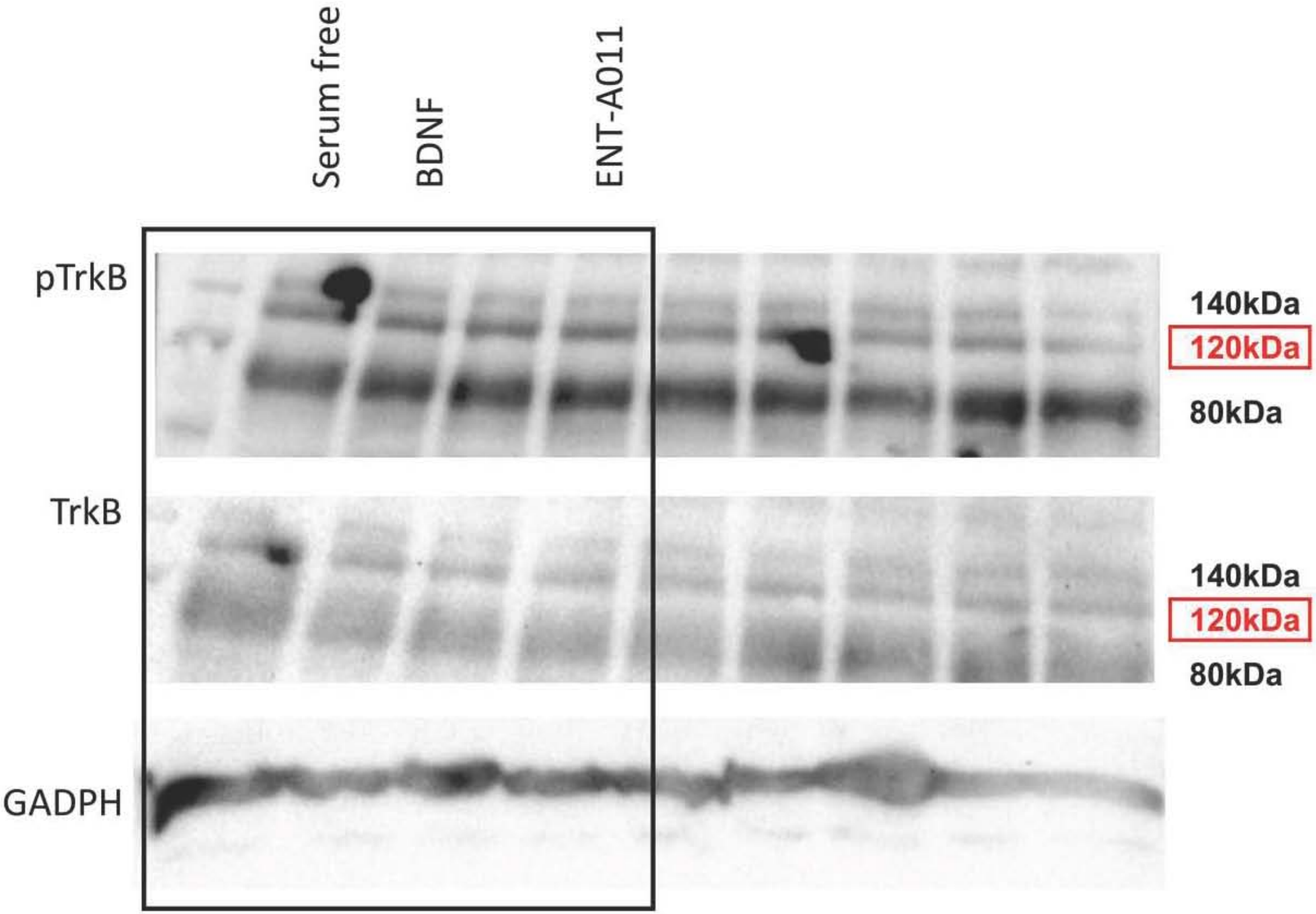

Figure 1A_WB4

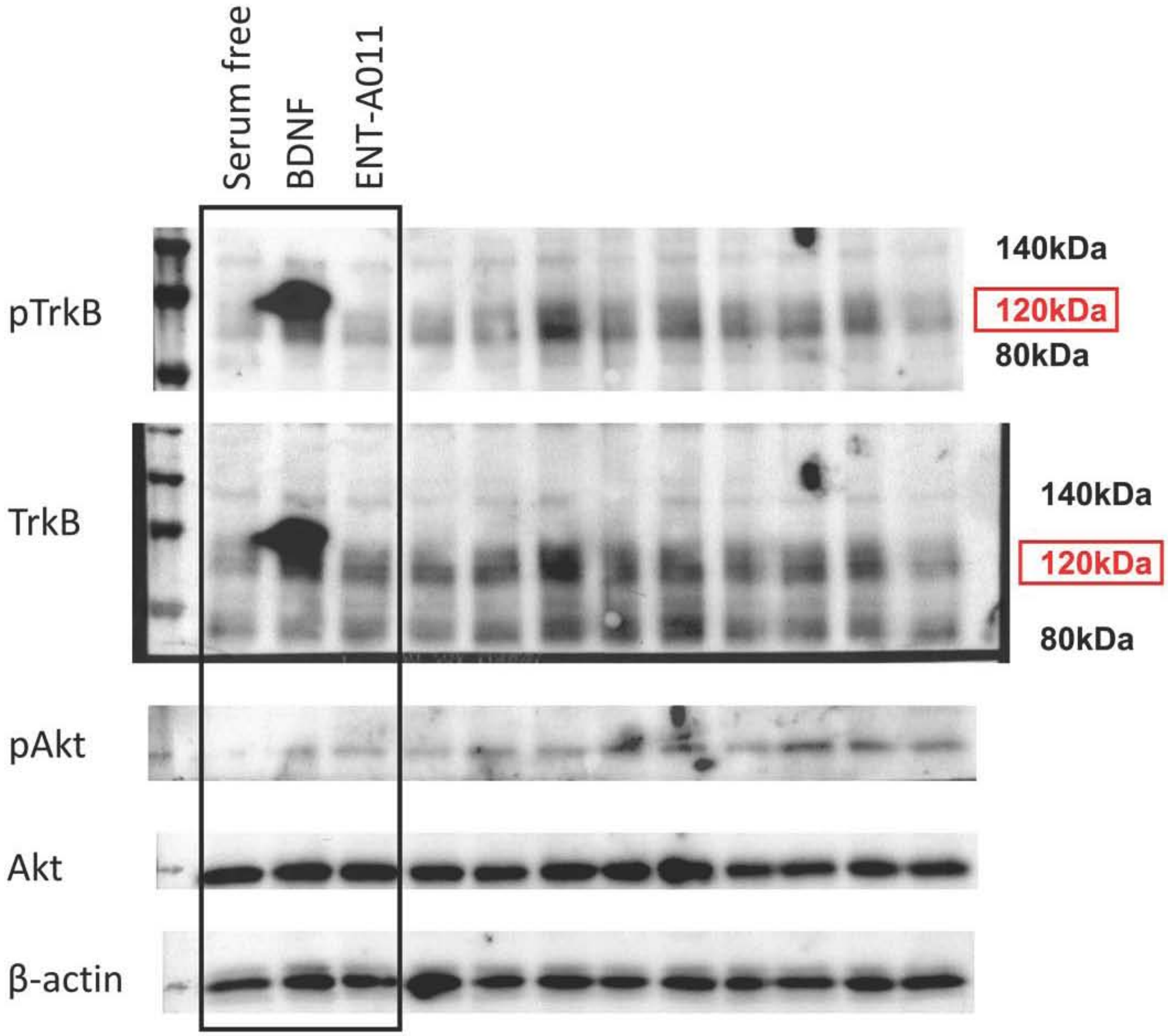

Figure 1A_WB5

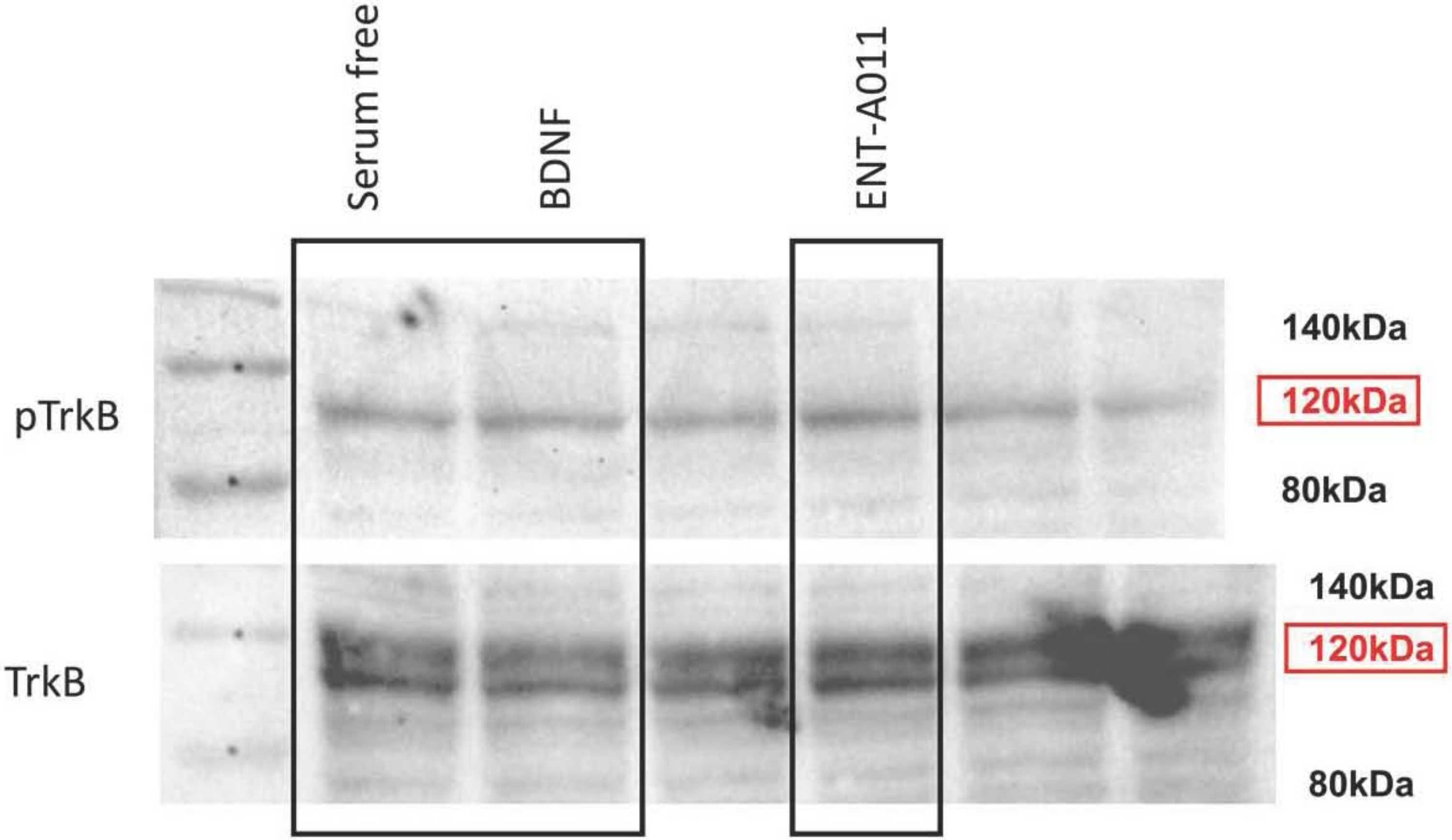

Figure 1A_WB6

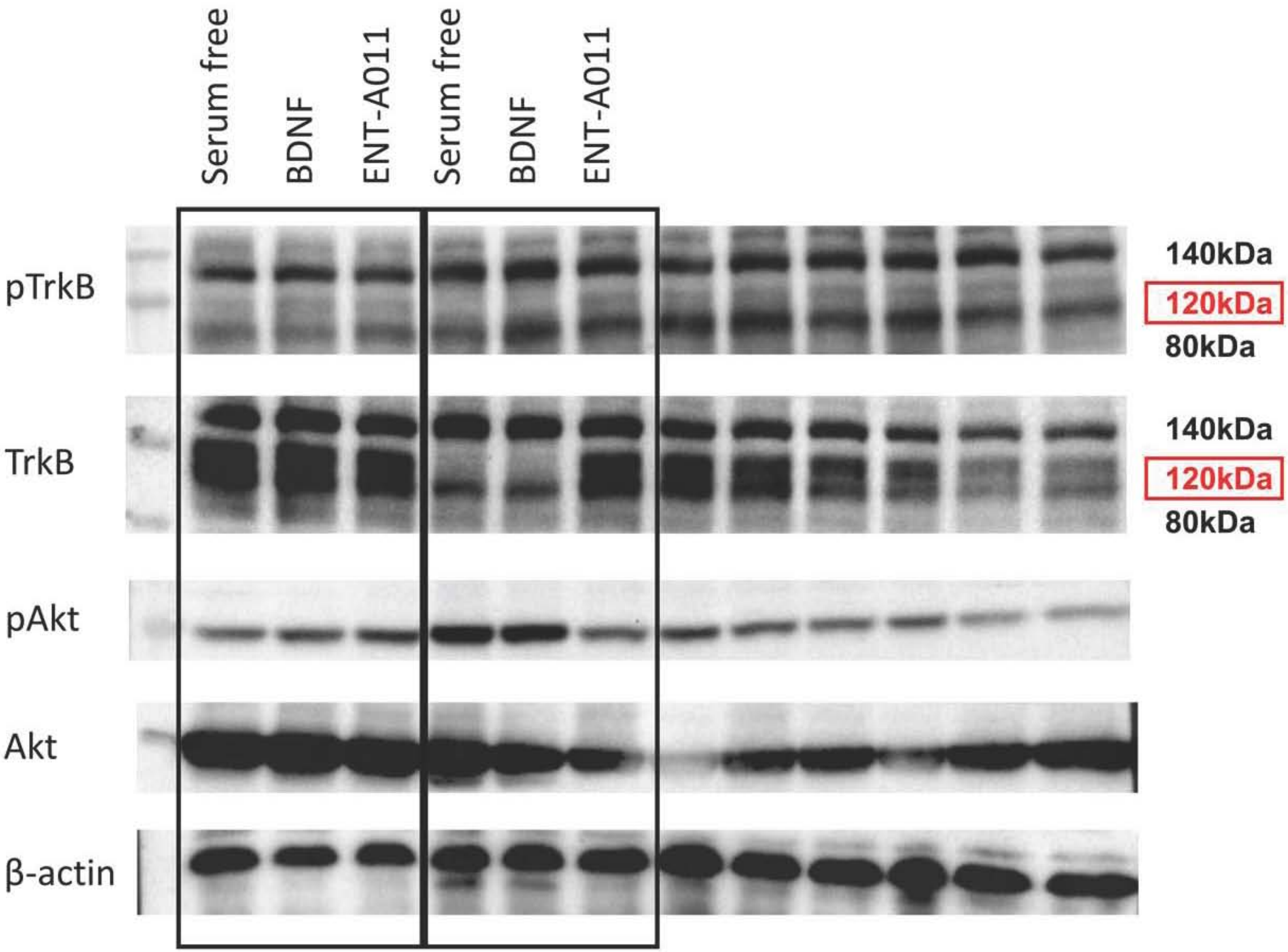

Figure 1A_WB7

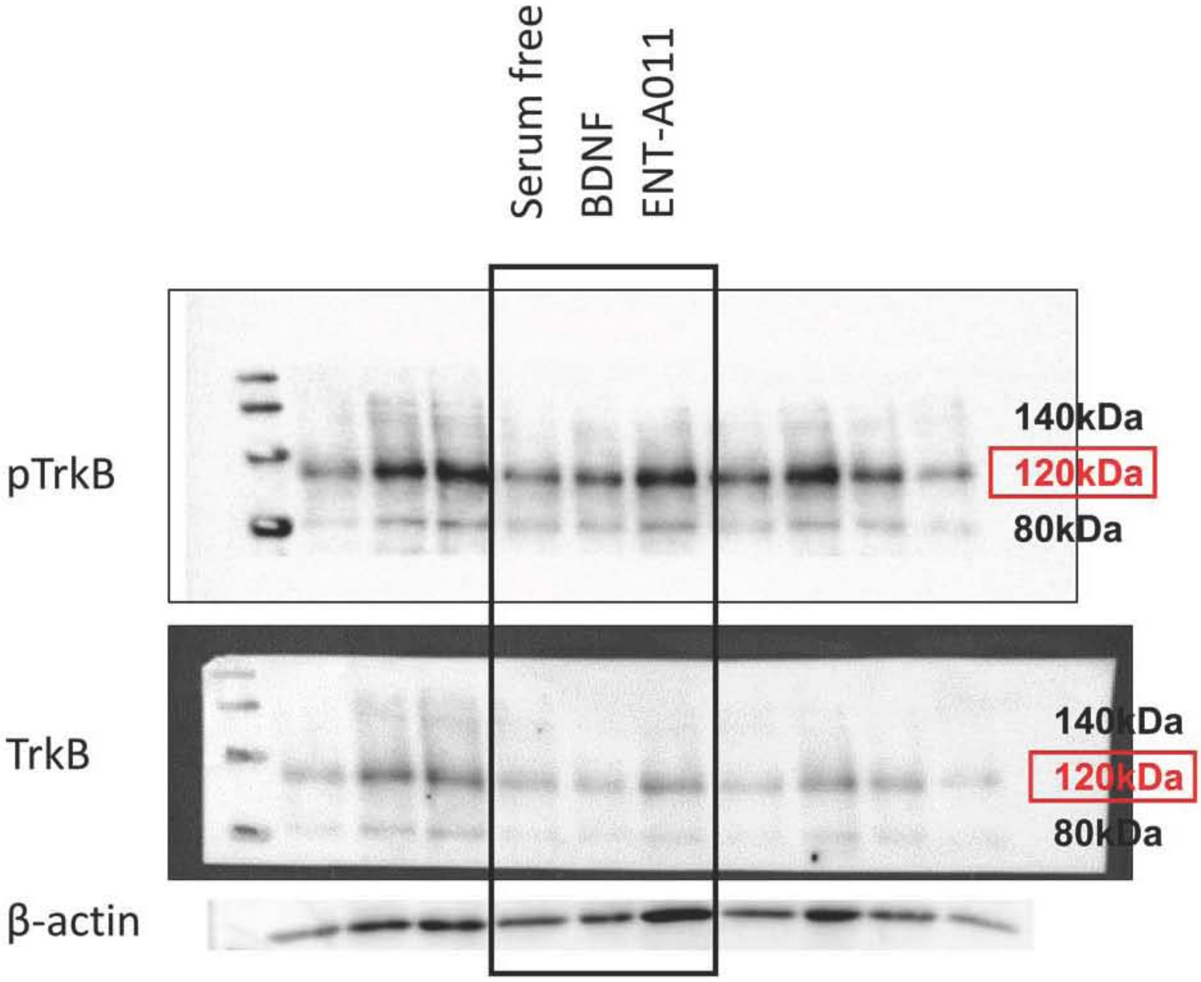

Figure 1A_WB8

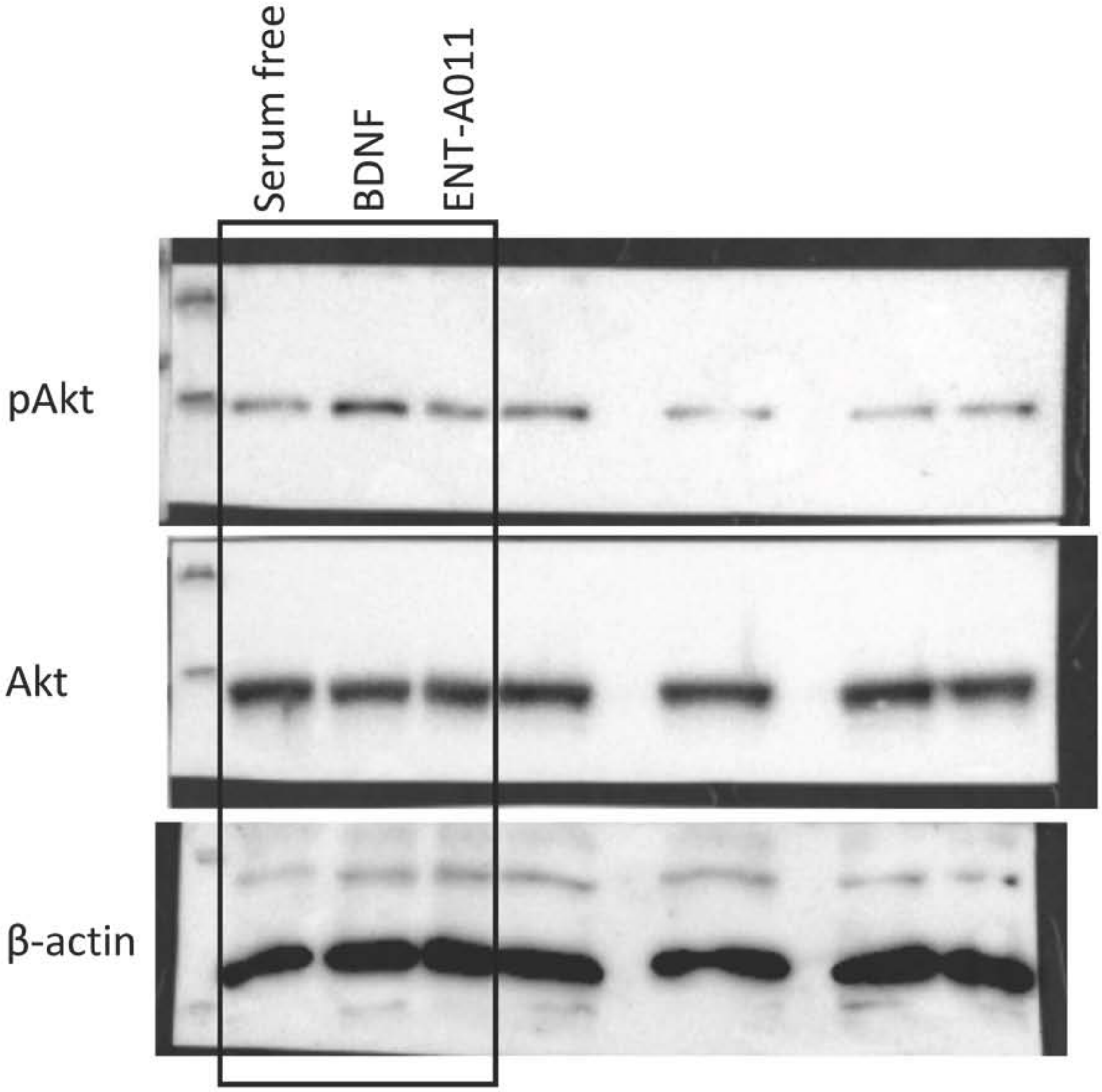

Figure 1A_WB9

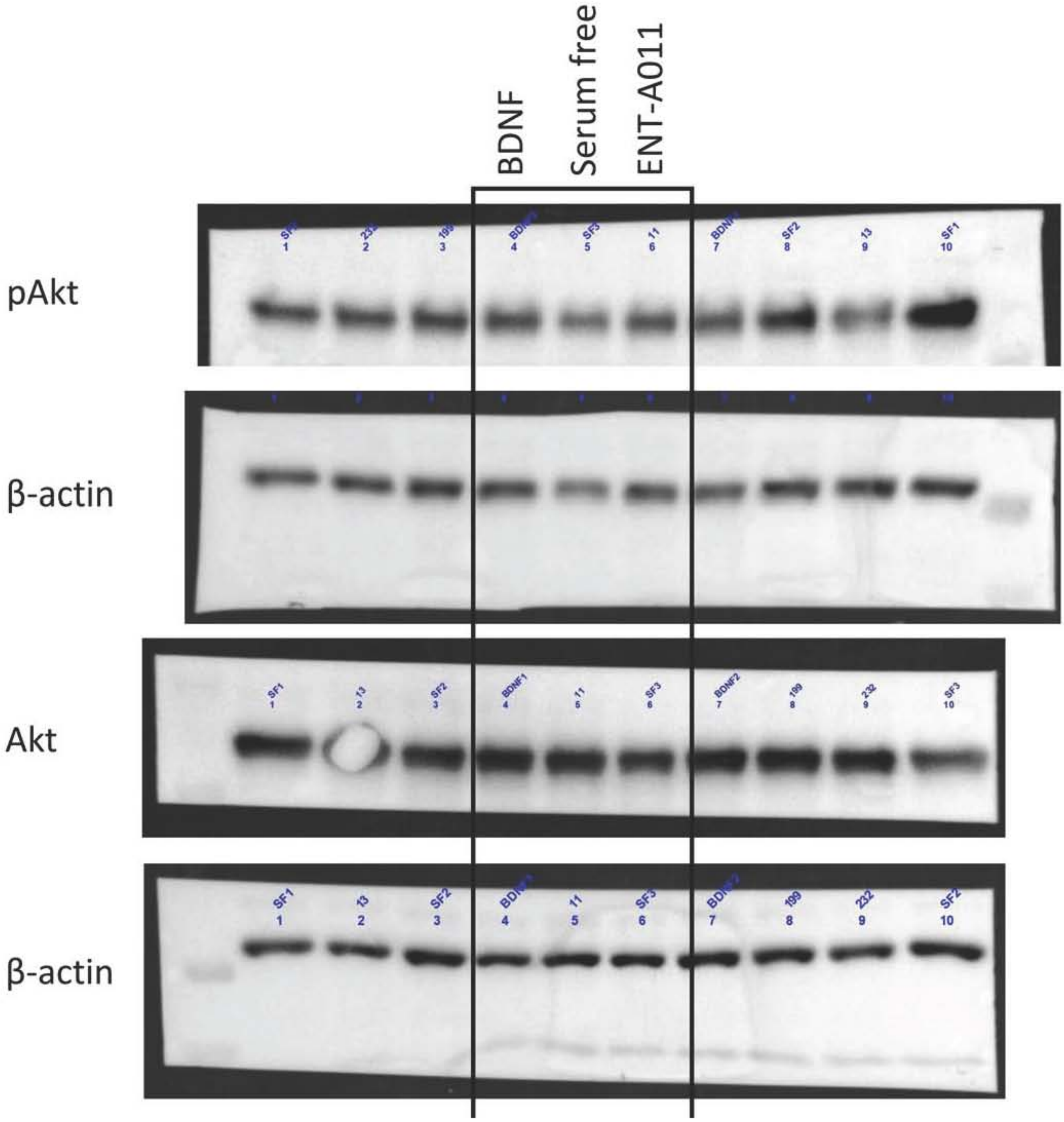

Figure 2A_WB1

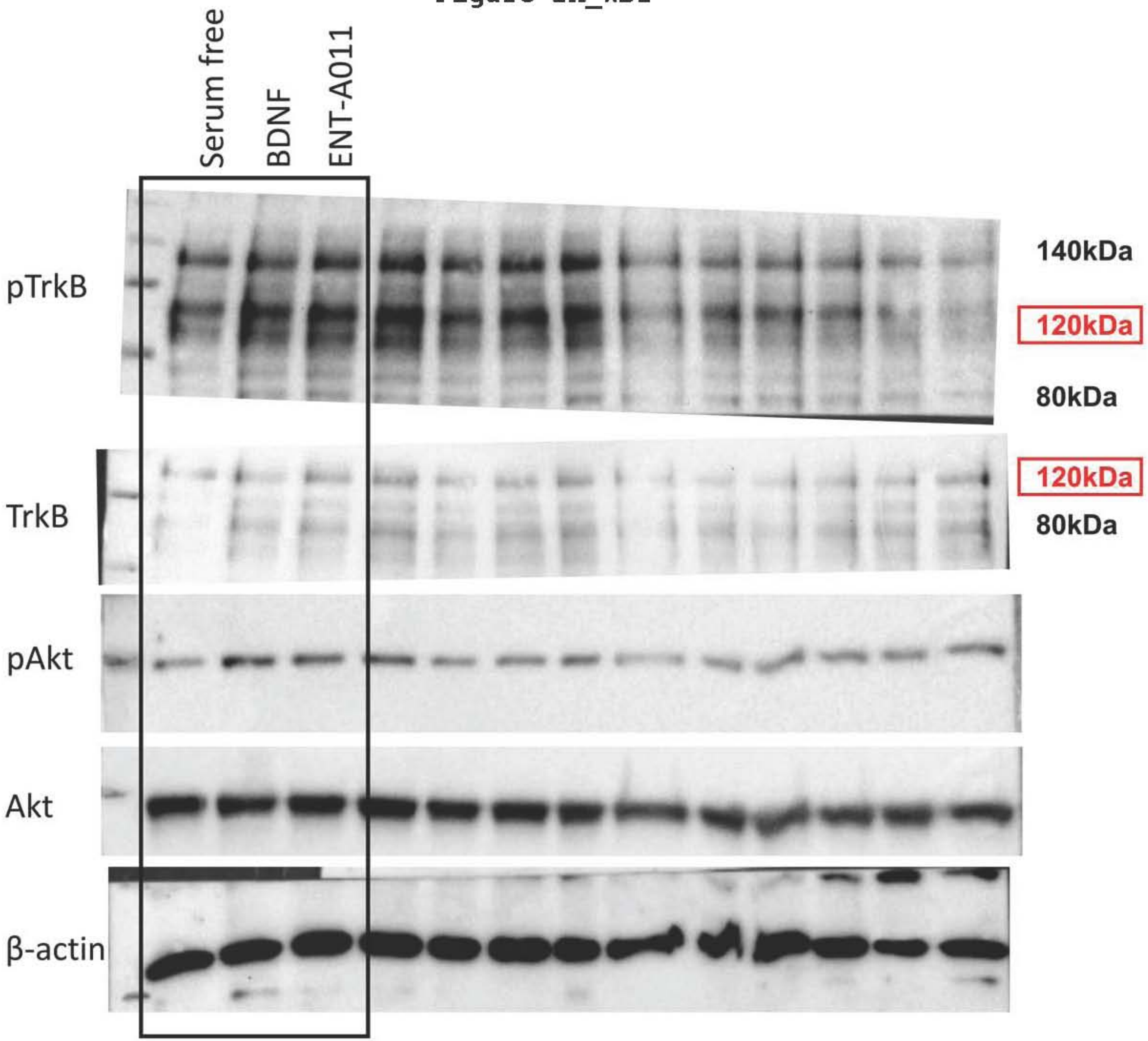

Figure 2A_WB2

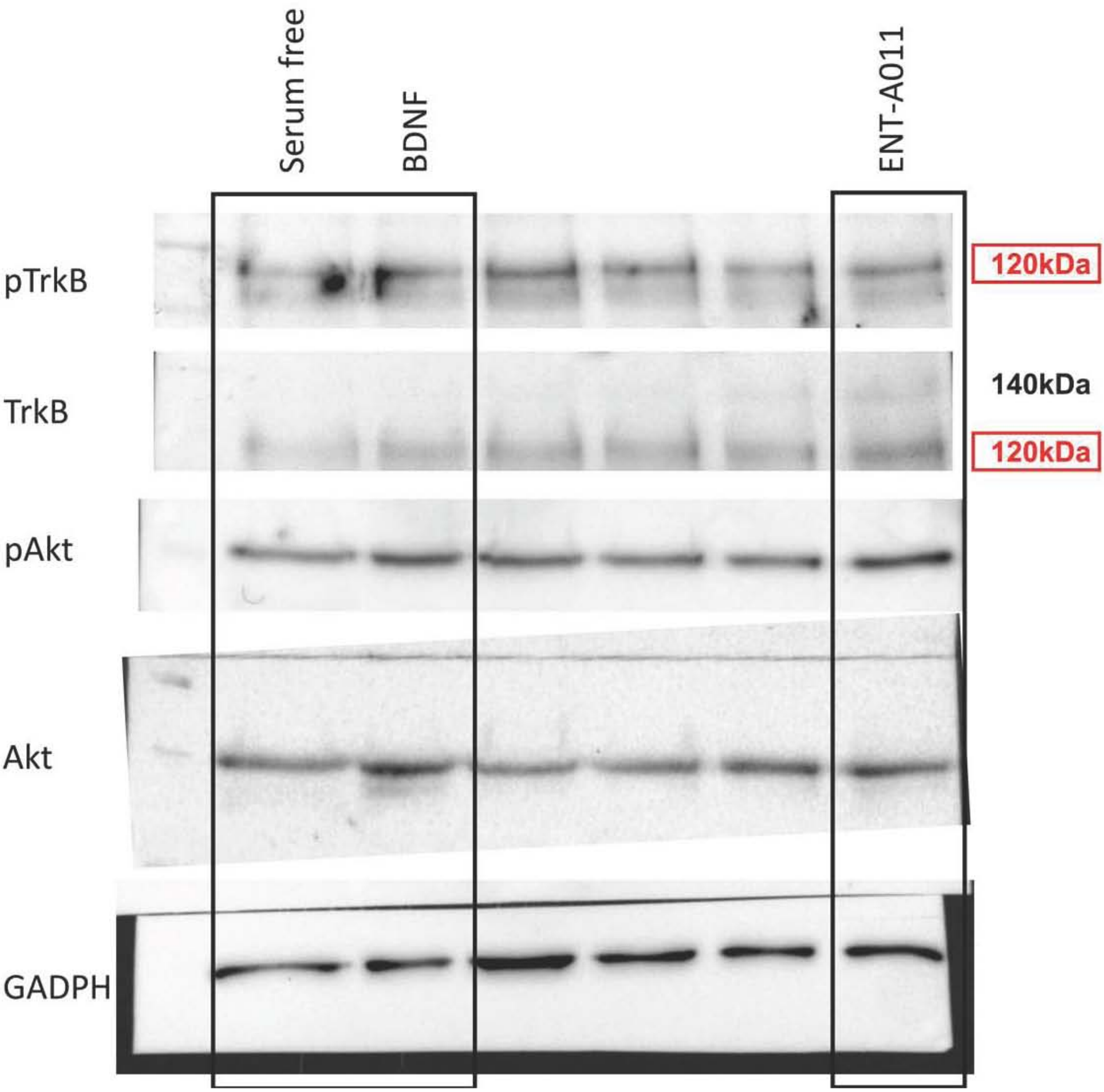

Figure 2A_WB3

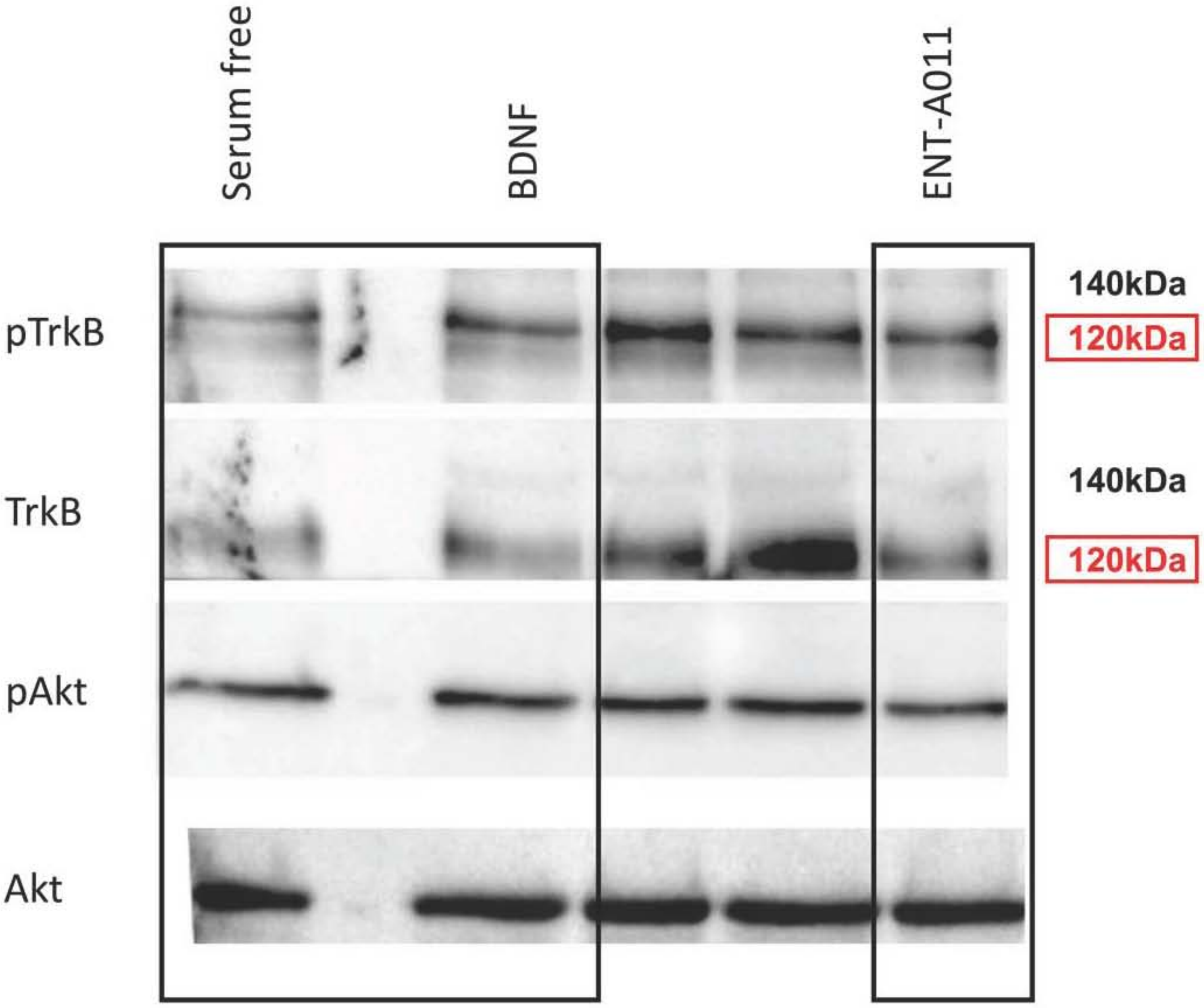

Figure 2A_WB4

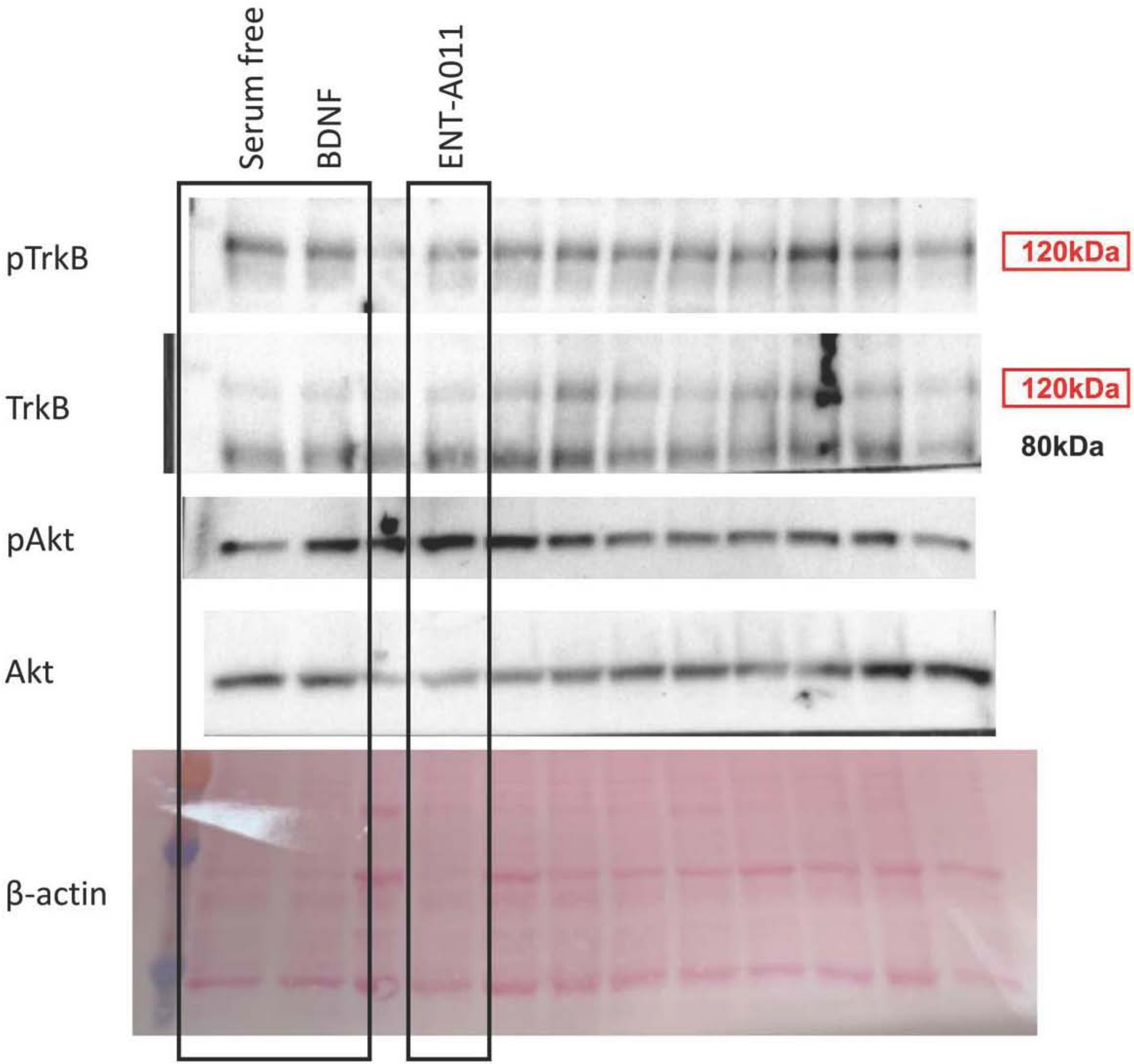

Figure 2A_WB5

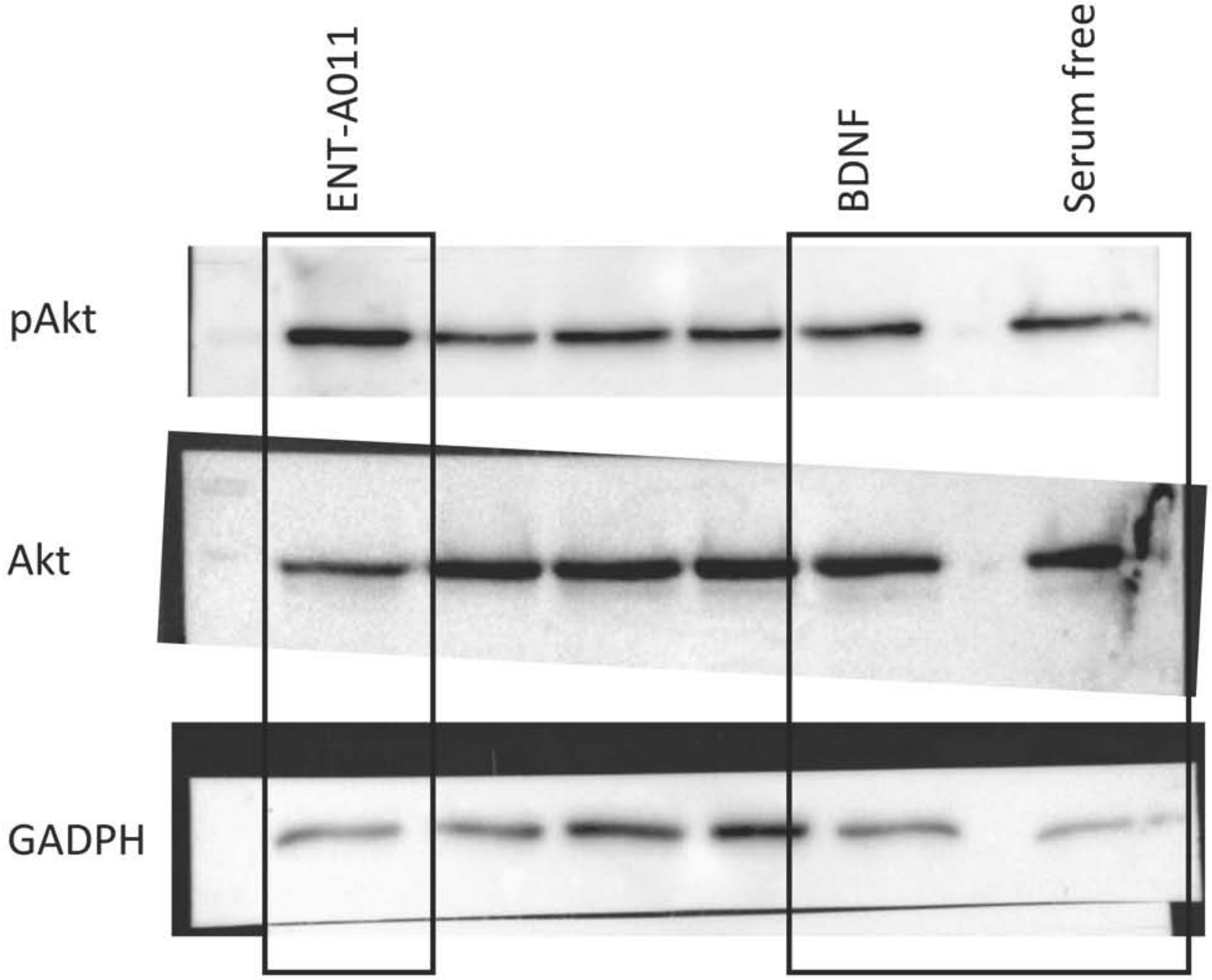

Supplement: Supplementary file 4 — Additional file 4. [file 13287_2024_3818_MOESM4_ESM.pdf]
