# Supplementary material for: Comprehensive characterization of the neurogenic and neuroprotective action of a novel TrkB agonist using mouse and human stem cell models of Alzheimer’s disease
Source: Stem Cell Res Ther. 2024 Jul 6;15:200. doi: 10.1186/s13287-024-03818-w (PMC11227723; doi:10.1186/s13287-024-03818-w)
Supplement: Supplementary file 5 — Additional file 5. [file 13287_2024_3818_MOESM5_ESM.pdf]

Comprehensive characterization of the neurogenic and neuroprotective action of a novel TrkB agonist using mouse and human stem cell models

SUPPLEMENTARY DATA

Supplementary Methods

Molecular modelling

The crystal structure of the complex of the TrkB immunoglobulin-like domain 5 (D5) homodimer with the mature neurotrophin-4/5 (NT-4/5) homodimer (PDB ID: 1HCF)[1] was used for modeling studies. The first three residues of the TrkB-D5 are cloning artefacts and thus they were mutated to the wild-type sequence. The structure of NT-4/5 has the following missing residues: Asn 145 – Gly 150 and Gly 207 – Ala 210 in chain A and Gly 81 – Glu 84, Asn 145, Arg 209 and Ala 210 in chain B (residue numbers correspond to the UniProt sequence of human NT-4/5, UniProt code: P34130). The missing residues were built with the AutoModel class of MODELLER v.9.23[2]. The linker corresponding to residues Asn 145 – Gly 150 was built using the structure of human NT-4/5 from PDB ID: 1B98[3] as a template. The final model was prepared with the Protein Preparation Wizard[4,5] of Schrödinger Suite v.2020. Specifically, bond orders were assigned, hydrogens were added, disulfide bonds were created, protonation states were assigned at pH 7 with PROPKA,[6] and optimization of the H-bond network and restrained energy minimization using the OPLS3e force field[7] with heavy atoms restricted to a maximum root mean squared deviation (RMSD) from the initial structure of 0.3 Å were performed. For the identification of possible binding sites, the SiteMap tool[8–10] of the Schrödinger Suite was used. From the predicted binding sites, the corresponding sites 1a and 1b, previously described[11] as the possible binding sites of the TrkA agonist BNN27, were used for docking studies. Compound ENT-A011 was built in Maestro[12] and prepared with the LigPrep tool[13] of

the Schrödinger Suite. The compound was docked to the two symmetric sites 1b of the TrkB - NT-4/5 complex using the standard precision (SP) protocol of the Glide tool[14–17] of the Schrödinger Suite. Grids were generated with Glide to describe the physicochemical properties of the protein pockets. The hydroxyl groups of the serine, threonine and tyrosine residues and the thiol groups of cysteine residues located within the grid box that describes the binding sites were allowed to rotate during the docking calculation in order to capture all possibilities for making hydrogen bonds with the ligands. For site 1b, the Glide SP scores for the two compounds were about -3.5 kcal/mol, indicating modest binding affinity. For site 1a, which is narrower than site 1b, ligand poses with favorable scores could not be obtained with rigid-body docking and therefore, the Induced Fit Docking (IFD) tool[18–21] of the Schrödinger Suite was used. This utilizes Glide for docking and Prime[22–24] for protein remodeling, and resulted in docking poses with scores of about -10.5 kcal/mol. In order to do a direct comparison of the docking scores in the two sites, IFD calculations of ENT-A011 in site 1b were also performed giving a docking score of -4.8 kcal/mol. The low scores for both SP and IFD protocols in site 1b are likely due to the higher solvent accessibility of this site compared to site 1a, which is less accessible and more buried at the interface of the receptor with the neurotrophin. Graphical analysis of the docking poses was carried out with VMD v.1.9.3[25].

TrkB Docking

To gain a structural understanding of the interaction of ENT-A011 with the TrkB receptor, we probed the binding mode of ENT-A011 to TrkB with molecular docking studies. The results showed that ENT-A011 can interact with TrkB similarly to the way the previously identified TrkA agonists BNN27 and ENT-A013 interact with TrkA. Specifically, ENT-A011 can bind in the two pockets (Figure S6) that exist at the interface of the extracellular domain of the TrkB receptor with the neurotrophin and can thus bridge the interactions between neurotrophin and receptor as a possible mode of action. Further information about the computational results can be found in the supplementary data.

Chemistry

Reactions were run in flame-dried glassware under an atmosphere of argon or nitrogen. All solvents were dried and/or purified according to standard procedures prior to use. Melting points were determined with an Electrothermal Digital Melting Point Apparatus, Cole-Parlmer ET0001/Version 1.0, and were uncorrected. Optical rotations were measured with a P3000 series polarimeter (Krüss Optronic, Hamburg, Germany). NMR spectra were recorded on Varian spectrometers (Varian, Palo Alto, CA, USA). The specific rotation $[a]_D^{25}$ was calculated according to the formula $[a]_D^{25} = \frac{a}{c \cdot l}$. The concentration of the sample is expressed in g/mL. ^{1}H NMR spectra were recorded at 300 MHz or 600 MHz, ^{13}C NMR spectra were recorded at 75 MHz or 150 MHz and were internally referenced to residual solvent peaks. Chemical shifts are reported in δ units, parts per million (ppm) and coupling constants (*J*) are given in Hz. Low-resolution mass spectra were recorded on a LC-MSn Fleet mass spectrometer (Thermo Scientific,Waltham, MA, USA) using MeOH as solvent. High-resolution mass spectra (HRMS) were recorded on UPLC-MSn Orbitrap Velos mass spectrometer (Thermo Scientific). Flash column chromatography (FCC) was performed on silica gel 60 (230–400 mesh, Merck, Darmstadt, Germany) and thin-layer chromatography (TLC) on pre-coated glass plates 60 F254 (0.2 mm, Merck). Spots were visualized with UV light at 254 nm and phosphomolybdic acid stain (PMA, 10% in absolute ethanol). The purity of ENT-A011 and ENT-A012 was determined by high-performance liquid chromatography (HPLC) using Nucelosil 100-5 C18 HD column, 5μm (4.6 x 250 mm), eluting with H_2O, 0.1% HCOOH – MeOH, 0.1% HCOOH gradient, flow rate 1 mL/min, UV detection at λ = 237 nm. Gradient information: 0.0 – 5.0 min ramped from 5% H_2O, 0.1% HCOOH - 95% MeOH, 0.1% HCOOH to 100% MeOH, 0.1% HCOOH; 5.0 – 10.0 min held at 100% MeOH, 0.1% HCOOH; 10.0 – 25.0 min returned to 5% H_2O, 0.1% HCOOH - 95% MeOH, 0.1% HCOOH.

ENT-A011: t_R = 6.32 min, purity = 99.09%. ENT-A012: t_R = 6.32 min, purity = 98.47%. (Supplementary Data, Figures S3 and S4).

Chemical Synthesis

The synthesis of ENT-A011 and ENT-A012 is reported in Scheme 1 (Supplementary Data) and involves seven high-yielding steps starting from DHEA. Thus, Horner-Wadsworth-Emmons reaction of DHEA with triethylphosphonoacetate using sodium

ethoxide (EtONa) as base gave the (*E*)-*α,β*-unsaturated ester 1 in 96% yield [26,27], which was, in turn, reacted with tert-butyldimethylsilyl chloride (TBSCl) to afford the tert-butyldimethylsilyl-protected alcohol 2 in 93% yield. Selective reduction of the ester group in 2 using diisobutylaluminum hydride (DIBAL-H) gave the allylic alcohol 3 in quantitative yield, which was subjected to a Simmons–Smith cyclopropanation reaction in the presence of diiodomethane and diethylzinc to yield the (17*S*,20*S*)-cyclopropyl derivative 4 in 60% yield after purification[28,29]. Compound 4 was subsequently oxidized with Dess–Martin periodinane (DMP) in dichloromethane (DCM) to afford the corresponding aldehyde 5 in 84% yield. Horner-Wadsworth-Emmons reaction of aldehyde 5 with diethyl(cyanomethyl)phosphonate in the presence of NaH afforded the *α,β*-unsaturated nitrile 6, as a mixture of *E,Z* geometrical isomers 6-*E* and 6-*Z* in 60:40 ratio, respectively [30]. Compounds 6-*E* and 6-*Z* were easily separated by flash column chromatography (FCC) and were deprotected separately using HF·Pyridine complex in dry CH_2Cl_2 to yield the final compounds ENT-A011 and ENT-A012, respectively, in quantitative yield. The geometry of the double bond in compounds ENT-A011 and ENT-A012 was confirmed by the corresponding coupling constants of the two vinylic protons of the unsaturated cyano functionality. In particular, in ENT-A011 they resonate at 5.31 ppm (d, *J* = 16.0 Hz, 1H) and 6.30 ppm (dd, *J* = 16.0, 10.2 Hz, 1H) and the coupling constant is equal to 16.0 Hz indicating the *trans* stereochemistry of the double bond (Supplementary Data, Figure S1). In addition, the *cis* stereochemistry in ENT-A012 is confirmed by the coupling constant (J) of the two vinylic protons being equal to 10.8 Hz [5.19 ppm (d, *J* = 10.8 Hz, 1H) and 6.03 ppm (t, *J* = 10.8 Hz, 1H)] (Supplementary Data, Figure S2).

Synthesis of ENT-A011 and ENT-A012 Details

Synthesis of (E)-(3β-Hydroxy-5-androsten-17-ylidene) ethyl ester (1)

To a solution of DHEA (2.0 g, 6.94 mmol) and triethyl phosphonoacetate (15.46 mL, 77.9 mmol) in anhydrous tetrahydrofuran (THF)/absolute ethanol 1:1 (21.4/21.4 mL) was added dropwise at 25 °C a solution of sodium ethoxide (EtONa) in absolute ethanol (prepared from 1.59 g Na in 30 mL ethanol). The resulting mixture was refluxed overnight. Subsequently, the reaction was cooled to 0 °C and was carefully quenched with water and acidified using 10% aq. HCl until completion of formation of

a precipitate. The solid was filtered under reduced pressure, washed with water (30 mL × 3 times) and petroleum ether 40–60 °C (30 mL × 3 times), and air-dried. The solid was dissolved in CH_2Cl_2 and the solution was dried over anhydrous Na_2SO_4 and filtered. The filtrate was then evaporated under reduced pressure to afford compound **1** (2.39 g, 96% yield) as a pure white crystalline solid. The compound was used in the next step without further purification. Mp: 178–180 °C; $[\alpha]_D^{25}$= −78° (c = 0.006 g/mL, $CHCl_3$); R_f: 0.5 (petroleum ether 40–60 °C/acetone 80:20); ^{1}H NMR (600 MHz, $CDCl_3$): δ 0.84 (s, 3H), 1.03 (s, 3H), 1.28 (t, J = 7.1 Hz, 3H, OCH_2CH_3), 1.31–2.34 (m, 18H), 2.78–2.90 (m, 2H), 3.48–3.57 (m, 1H, 3α-H), 4.15 (q, J = 7.1 Hz, 2H, OCH_2CH_3), 5.36 (d, J = 5.0 Hz, 1H, 6-H), 5.55 (bs, 1H, 20-H); ^{13}C NMR (150 MHz, $CDCl_3$): δ 14.5, 18.4, 19.6, 21.1, 24.6, 30.6, 31.7, 31.8, 35.3, 36.8, 37.4, 42.4, 46.2, 50.4, 54.0, 59.7, 71.7, 71.8, 108.8, 121.5, 140.9, 167.6, 176.3; APCI-HRMS: m/z calculated for $[M+H]^+$ $C_{23}H_{35}O_3$ 359.2581, found 359.2579.

Synthesis of (E)-[3β-(t-butyldimethylsilyloxy)-5-androsten-17-ylidene] ethyl ester (2)

To a solution of compound 1 (2.25 g, 6.28 mmol) in anhydrous THF (20 mL) imidazole (1.33 g, 19.5 mmol) and iodine (4.76 g, 37.5 mmol) were added at 0 °C and the mixture was stirred at 0 °C for 30 min. Subsequently, *tert*-butyldimethylsilyl chloride (1.05 g, 6.67 mmol) was added and the resulting mixture was stirred at 25 °C overnight. After completion of the reaction, the solvent was evaporated in vacuo and the residue was diluted and extracted using ethyl acetate (EtOAc). The organic layer was washed using sat. aq. $Na_2S_2O_4$, brine and dried over anhydrous Na_2SO_4. The solvent was removed *in vacuo* and the residue was purified by FCC (elution solvent: petroleum ether 40–60 °C/ethyl acetate 95:5) to afford compound 2 as a white solid (2.36 g, 93% yield). Mp: 100–102 °C; $[\alpha]_D^{25}$= −50° (c= 0.0056 g/mL, $CHCl_3$); R_f: 0.86 (petroleum ether 40–60 °C/EtOAc 80:20); ^{1}H NMR (600 MHz, $CDCl_3$): δ 0.05 (s, 6H, $Si(CH_3)_2$), 0.83 (s, 3H), 0.89 (s, 9H, $C(CH_3)_3$), 1.02 (s, 3H), 1.28 (t, J = 7.1 Hz, 3H, OCH_2CH_3), 1.32–2.31 (m, 17H), 2.79–2.89 (m, 2H) 3.42–3.54 (m, 1H, 3α-H), 4.15 (q, J = 7.1 Hz, 2H, OCH_2CH_3), 5.33 (d, J = 5.3 Hz, 1H, 6-H), 5.54 (t, J = 2.3 Hz, 1H, 20-H); ^{13}C NMR (75 MHz, $CDCl_3$): δ -4.3, 14.5, 18.4, 19.6, 21.1, 24.6, 26.1, 30.6, 31.7, 31.8, 32.2, 35.4, 36.8, 37.5, 42.9, 46.2, 50.5, 54.0, 59.7, 72.7, 108.8, 120.9, 141.8, 167.6, 176.4; APCI-HRMS: m/z calculated for $C_{29}H_{49}O_3Si$ $[M+H]^+$ 473.3445, found 473.3443.

Synthesis of (E)-3β-(t-butyldimethylsilyloxy)-pregna-5,17(20)-dien-21-ol (3)

To a solution of compound **2** (2.20 g, 4.65 mmol) in anhydrous THF (94 mL) was added dropwise at −78 °C diisobutylaluminum hydride (DIBAL-H) [(1.0 M in hexane), 18.7 mL, 18.7 mmol]. The reaction mixture was stirred at −78 °C for 2.5 h and at 25 °C for an additional 1 h. After completion of the reaction, saturated aqueous NH_4Cl (40 mL) was added at 0 °C and the solvent was evaporated in vacuo. EtOAc (30 mL) was added to the residue and the organic layer was washed using 10% aq. HCl (20 mL) and brine (40 mL), dried over anhydrous Na_2SO_4 and filtered. The filtrate was then evaporated under reduced pressure to afford compound 3 as a white pure crystalline solid (2.0 g, quantitative yield). Compound **3** was used in the next step without further purification. Mp: 129–131 °C; $[\alpha]_D^{25}$= −39° (c = 0.0054 g/mL, $CHCl_3$); R_f: 0.46, (petroleum ether 40–60 °C/EtOAc 80:20); ^{1}H NMR (600 MHz, $CDCl_3$): δ 0.06 (s, 6H, $Si(CH_3)_2$), 0.78 (s, 3H), 0.89 (s, 9H, $C(CH_3)_3$), 1.02 (s, 3H), 1.19–2.43 (m, 18H), 3.43–3.53 (m, 1H, 3α-H), 4.09 (dd, J = 12.2, 6.7 Hz, 1H, 21-H), 4.15 (dd, J = 12.2, 6.7 Hz, 1H, 21-H) 5.25 (t, J = 6.7 Hz, 1H, 20-H), 5.32 (d, J = 5.1 Hz, 1H, 6-H); ^{13}C NMR (75 MHz, $CDCl_3$): δ −4.4, 14.3, 18.4, 18.7, 19.6, 21.1, 21.2, 24.5, 26.1, 26.3, 31.8, 31.9, 32.2, 35.8, 36.9, 37.5, 42.9, 44.0, 50.7, 72.7, 115.7, 121.0, 141.8, 155.9; APCI-HRMS: m/z calculated for $C_{21}H_{31}O$ [(M-TBS-H_2O)+H]$^+$ 299.2369, found 299.2367.

Synthesis of (17S,20S)-3β-(t-butyldimethylsilyloxy)-17,20-methan-5-pregnene-21-ol (4)

To a solution of compound **3** (1.8 g, 4.17 mmol) in anhydrous toluene (33.6 mL) was added at 25 °C diiodomethane (CH_2I_2) (1.8 mL, 21.6 mmol). The reaction mixture was then cooled to −78 °C and a solution of diethylzinc (0.9 M in hexane, 24 mL, 21.6 mmol) was added. The reaction mixture was then stirred at 25 °C for 2 h. After completion of the reaction (monitored by ^{1}H NMR), the reaction mixture was quenched using 10% aqueous HCl at 0 °C until pH 5.5, followed by extraction using EtOAc (30 mL × 3 times). The organic layer was washed with brine, dried over anhydrous Na_2SO_4 and the solvent was removed *in vacuo.* The residue was purified by FCC (petroleum ether 40–60 °C/EtOAc 90/10 → 85/15) to obtain compound **4** (1.1 g, 60% yield). Mp: 154–157° C; $[\alpha]_D^{25}$= −50° (c= 0.0036 g/mL, $CHCl_3$); R_f: 0.46 (petroleum ether 40–60 °C/EtOAc 80:20); ^{1}H NMR (600 MHz, $CDCl_3$): δ 0.06 (s, 6H, $Si(CH_3)_2$), 0.78 (s, 3H), 0.89 (s, 9H, $C(CH_3)_3$), 0.90–0.97 (m, 1H) 1.01 (s, 3H), 1.03–2.32 (m, 20H), 3.45–3.54

(m, 2H), 3.58–3.66 (m, 1H, 22-H), 5.33 (d, J = 5.3 Hz, 1H, 6-H); ^{13}C NMR (75 MHz, $CDCl_3$): δ −4.4, 16.3, 17.1, 18.4, 19.6, 19.8, 20.6, 25.2, 26.1, 29.1, 32.1, 32.2, 32.7, 33.3, 36.3, 36.8, 37.5, 41.1, 42.9, 50.6, 54.9, 65.3, 72.7, 121.1, 141.7; APCI-HRMS: m/z calculated for $C_{28}H_{47}OSi$ [(M-H_2O)+H]$^+$ 427.3391, found 427.3386.

Synthesis of (17S,20S)-3β-(t-butyldimethylsilyloxy)-17,20-methan-5-pregnene-21-carbaldehyde (5)

To a solution of compound 4 (0.85 g, 1.91 mmol) in dry DCM (100 mL) was added at 0 °C Dess-Martin periodinane (1.62 g, 3.82 mmol) and the reaction mixture was stirred at room temperature for 1.5 hours. After completion of the reaction (checked by TLC), a mixture of saturated aqueous $NaHCO_3$ and 10% aq. $Na_2S_2O_3 \cdot 5H_2O$ (1:1) was added and the reaction mixture was stirred for 30 min. The reaction mixture was extracted with Et_2O (30 mL x 3 times) and the combined organic layers washed with saturated aqueous $NaHCO_3$ and brine, dried over Na_2SO_4 and the solvent was removed *in vacuo*. The residue was purified by FCC (petroleum ether 40-60 °C/EtOAc 98:2→96:4) to afford compound 5 (0.72 g, yield 85%) as a white crystalline solid. Mp: 114–116 °C; $[\alpha]_D^{25}$= −21° (c= 0.0056 g/mL, $CHCl_3$); R_f: 0.78 (petroleum ether 40–60 °C/EtOAc 80:20); ^{1}H NMR (600 MHz, $CDCl_3$): δ 0.05 (s, 6H, $Si(CH_3)_2$), 0.80 (s, 3H), 0.88 (s, 9H, $C(CH_3)_3$), 0.92–0.99 (m, 1H), 1.00 (s, 3H), 1.04–2.30 (m, 21H), 3.42–3.52 (m, 1H, 3*α*-H), 5.33 (d, J= 5.1 Hz, 1H, 6-H), 9.08 (d, J = 6.2 Hz, 1H, CHO); ^{13}C NMR (75 MHz, $CDCl_3$): δ -4.4, 17.1, 18.4, 19.6, 20.5, 21.1, 25.3, 26.0, 29.6, 31.0, 32.0, 32.1, 32.6, 33.1, 36.8, 37.5, 42.3, 42.9, 43.8, 50.4, 53.9, 72.6, 120.9, 141.7, 202.0; APCI-HRMS: m/z calculated for $C_{28}H_{47}O_2Si$ [M+H]$^+$ 443.3340, found 443.3339.

Synthesis of 3-((2'R,3S,17S)-3-((t-butyldimethylsilyl) oxy)-5-androstene-17,1'-cyclopropan]-2'-yl) acrylonitrile (6)

To a suspension of NaH 60% in mineral oil (30 mg, 0.70 mmol) in dry THF was added at 0 °C diethyl(cyanomethyl)phosphonate (0.1 mL, 0.64 mmol) and the reaction mixture was stirred at room temperature for 30 minutes. Subsequently, a solution of compound **5** (70 mg, 0.16 mmol) in dry THF (0.1 M) was added at 0°C and the reaction mixture was stirred at the same temperature for 30 minutes. After completion of the reaction, the reaction mixture was quenched with saturated aqueous NH_4Cl and extracted with EtOAc. The organic layer was washed with brine, dried over Na_2SO_4 and the solvent was removed *in vacuo*. The residue was purified by FCC (petroleum

ether 40-60 °C/EtOAc 99:1→98:2) to afford compound **6-*Z*** (23 mg) and **6-*E***, (36 mg) in 84% overall yield as white crystalline solids.

(Z)-3-((2'R,3S,17S)-3-((t-butyldimethylsilyl)oxy)-5-androstene-17,1'-cyclopropan]-2'-yl) acrylonitrile (6-Z)

Mp: 130-132 °C; $[\alpha]_D^{25}$= -117.6° (c= 0.0034 g/mL, $CHCl_3$); R_f: 0.67, petroleum ether 40-60 °C/EtOAc 90:10; ^1H NMR (600 MHz, $CDCl_3$): *δ* 0.06 (s, 6H, Si(CH_3)$_2$), 0.54 (t, *J* = 5.0 Hz, 1H), 0.81 (s, 3H), 0.89 (m, 9H), 1.01 (s, 3H), 1.12-2.31(m, 20H), 3.49-3.53 (m, 1H, 3*α*-H) 5.32 (d, *J* = 4.9 Hz, 1H, 6-H), 5.19 (d, *J* = 10.8 Hz, 1H), 6.03 (t, *J* = 10.8 Hz, 1H); ^{13}C NMR (150 MHz, $CDCl_3$): *δ* -4.4, 16.7, 18.4, 19.6, 20.5, 22.2, 23.3, 25.2, 26.1, 29.9, 32.1, 32.2, 32.6, 33.7, 36.9, 37.6, 42.1, 42.3, 42.9, 50.5, 54.5, 72.7, 95.9, 117.2, 120.9, 141.8, 158.0; APCI-HRMS: m/z calculated for $C_{30}H_{48}NOSi$ $[M+H]^+$ 466.3500, found 466.3496.

(E)-3-((2'R,3S,17S)-3-((t-butyldimethylsilyl)oxy)-5-androstene-17,1'-cyclopropan]-2'-yl) acrylonitrile (6-E)

Mp: 193-196 °C; $[\alpha]_D^{25}$= + 40° (c= 0.0015 g/mL, $CHCl_3$); R_f: 0.64, petroleum ether 40-60 °C/EtOAc 90:10; ^1H NMR (600 MHz, $CDCl_3$): *δ* 0.06 (s, 6H, Si(CH_3)$_2$), 0.53 (t, *J* = 4.9 Hz, 1H), 0.75 (s, 3H), 0.89 (m, 9H), 1.00 (s, 3H), 1.12-2.28 (m, 20H), 3.49-3.53 (m, 1H, 3*α*-H) 5.30 (*J* = 3.0 Hz, 1H, 6-H), 5.31 (d, *J* = 16.0 Hz, 1H), 6.30 (dd, *J* = 16.0, 10.2 Hz); ^{13}C NMR (150 MHz, $CDCl_3$): *δ* -4.4, 16.7, 18.4, 19.6, 20.5, 22.9, 23.1, 25.2, 26.1, 29.9, 32, 32.2, 32.6, 33.3, 36.8, 37.5, 42.2, 42.3, 42.9, 50.4, 54.6, 72.6, 96.2, 118.3, 121, 141.7, 159; APCI-HRMS: m/z calculated for $C_{30}H_{48}NOSi$ $[M+H]^+$ 466.3500, found 466.3497.

Synthesis of (E)-3-((2'R,3S,17S)-3-(hydroxy)-5-androstene-17,1'-cyclopropan]-2'-yl) acrylonitrile (ENT-A011)

To a solution of compound **6-*E*** (34 mg, 0.07 mmol) in anhydrous DCM (2.3 mL) HF·pyridine (0.1 mL) was added at 0 °C and the reaction mixture was stirred at room temperature for 2 hours. The reaction was quenched with water at 0 °C, and the resulting mixture was extracted with DCM. The organic layer was washed with brine, dried over Na_2SO_4 and the solvent was removed *in vacuo*. The residue was purified by FCC (Hexane/EtOAc 90:10→80:20) to afford compound **ENT-A011** (25 mg, yield

quantitative) as a white crystalline solid. Mp: 180-183 °C; $[\alpha]_D^{25}$= +28° (c= 0.0029 g/mL, $CHCl_3$); R_f: 0.17, Hexane/EtOAc 80:20; ^1H NMR (600 MHz, $CDCl_3$): *δ* 0.53 (t, *J* = 4.9 Hz, 1H), 0.76 (s, 3H), 0.83-0.98 (m, 2H), 1.01 (s, 3H), 1.12-2.28 (m, 19H), 3.49-3.53 (m, 1H, 3*α*-H), 5.31 (d, *J* = 16.0 Hz, 1H), 5.36 (m, 1H, 6-H), 6.30 (dd, *J* = 16.0, 10.2 Hz, 1H); ^{13}C NMR (75 MHz, $CDCl_3$): *δ* 16.7, 19.5, 20.6, 22.9, 23.1, 25.1, 29.8, 31.7, 32.0, 32.5, 33.3, 36.7, 37.4, 42.1, 42.2, 42.3, 50.3, 54.5, 71.8, 96.2, 118.3, 121.6, 140.9, 159.1; APCI-HRMS: m/z calculated for $C_{24}H_{34}NO$ $[M+H]^+$ 352.2635, found 352.2628. HPLC: t_R = 6.32 min, λ = 237 nm, purity = 99.09%.

Synthesis of (Z)-3-((2'R,3S,17S)-3-(hydroxy)-5-androstene-17,1'-cyclopropan]-2'-yl)acrylonitrile (ENT-A012)

To a solution of compound **6-*Z*** (22 mg, 0.04 mmol) in anhydrous DCM (1.5 mL) HF·pyridine (70 μL) was added at 0 °C and the reaction mixture was stirred at 25 °C for 2 hours. The reaction was quenched with water at 0 °C and the resulting mixture was extracted with DCM. The organic layer was washed with brine, dried over Na_2SO_4 and the solvent was removed in vacuo. The residue was purified by FCC (Hexane/EtOAc 90:10→80:20) to afford compound **ENT-A012** (13 mg, yield quantitative) as a white crystalline solid. Mp: 197-203 °C; $[\alpha]_D^{25}$= -157° (c= 0.0049 g/mL, $CHCl_3$); R_f: 0.05, Hexane/EtOAc 90:10; ^1H NMR (600 MHz, $CDCl_3$): 0.54 (t, *J* = 5.0 Hz, 1H), 0.82 (s, 3H), 0.85-0.98 (m, 2H), 1.02 (s, 3H), 1.12-2.28 (m, 19H), 3.49-3.53 (m, 1H, 3α-H) 5.36 (d, *J* = 5.1 Hz, 1H, 6-H), 5.19 (d, *J* = 10.8 Hz, 1H), 6.03 (t, *J* = 10.8 Hz, 1H); ^{13}C NMR (75 MHz, $CDCl_3$): δ 16.8, 19.6, 20.5, 22.3, 23.3, 25.2, 29.8, 31.7, 32.0, 32.6, 33.2, 36.7, 37.4, 42.1 42.2, 42.3, 50.4, 54.4, 71.9, 95.9, 117.3, 121.5, 141.0, 158.1; APCI-HRMS: m/z calculated for $C_{24}H_{34}NO$ $[M+H]^+$ 352.2635 found 352.2632. HPLC: t_R = 6.32 min, λ = 237 nm, purity = 98.47%.

Metabolic stability

Incubation conditions ensured linear metabolite formation with respect to reaction time and protein concentration (pooled human liver microsome concentration was set at 0.5mg/mL). To determine the oxidative (CYP-mediated) metabolic stability profile, 1mM NADPH served as a cofactor. ENT-A011 was tested at 1μM. Triplicate reactions took place at 37 °C in the presence of negative and positive controls (low vs. rapid clearance). Reactions were terminated after 60 minutes and readouts were recorded

by Lionheart FX (BioTek) to determine the residual (%) of time zero (ENT-A011 depletion, Figure S7).

Isozyme-specific CYP450-metabolism

CYP1A2, CYP2A6, CYP2B6, CYP2C9, CYP2C19, CYP2D6, and CYP3A4 human CYP450 isoenzymes were expressed in Baculosomes®, purchased from Thermo Fisher Scientific (Waltham, MA, USA). All reagents were handled and prepared according to the manufacturer's protocol. ENT-A011 was tested at 1μM. On the basis of the kinetic model for each CYP450 isoform in question, CYP450-enzymatic activity was determined in the presence of ENT-A011. Triplicate reactions took place at 20°C, following their initiation via the conversion of NADP+ (10mM in 100mM potassium phosphate, pH 8.0) into NADPH by the regeneration system present (glucose-6-phosphate at 333mM and glucose-6-phosphate-dehydrogenase at 30 U/mL in 100mM potassium phosphate, pH 8.0). Next, the fluorescent substrate was added and immediately (< 2 minutes), signal monitoring over time took place at suitable excitation and emission wavelengths by Lionheart FX (BioTek). CYP450 inhibition (%) was determined based on the reaction rates (fluorescence intensity changes per unit time). In total, n=60 measurements per minute were acquired (t=60 minutes).

% Inhibition= (1- X/A) x 100% where X is the rate observed in the presence of test compound and A is the rate observed in the presence of negative (solvent, DMSO) control (Figure S7).

Supplementary Results

Computational Study of ENT-A011 TrkB Docking

Computational docking studies were performed to investigate the mechanism of action of the compound ENT-A011. The extracellular domain of TrkA has been previously reported to be a drug target[31–33], while prior Saturation Transfer Difference Nuclear Magnetic Resonance (STD-NMR) experiments and molecular dynamics simulations have indicated that an analogue of ENT-A011, BNN27, binds at the interface of TrkA-D5 with NGF, thus bridging the heterodimer.[34] Specifically, two binding sites, site 1a and site 1b, found at the interfaces of the two proteins were proposed to be the most

probable binding sites for BNN27.[34] Since there is high structural similarity between the TrkA and TrkB receptors, and ENT-A011 is an analogue of BNN27, the corresponding binding sites 1a and 1b of TrkB were identified in the present work and used for docking (Figure S6). The binding poses of the compound in the two sites show complementarity to the binding pockets. For site 1b, the docking pose has the 3β-hydroxyl group pointing towards the solvent region and the C17 steroid substituent facing towards the interface of TrkB-D5 and NT-4/5, thus providing a possible explanation for the selectivity of the substituent. The binding pose in site 1a is less solvent-exposed and preliminary molecular dynamics simulations showed that the compound bound in this pocket dissociates less readily than from site 1b (data not shown). Overall, the docking studies suggest two plausible interaction modes of ENT-A011 with TrkB that could lead to enhanced receptor activation.

ENT-A011 shows weak-to-moderate CYP inhibition

Human liver cytochrome P450 (CYP450) enzymes are crucial for xenobiotic biodegradation, metabolism, and toxicity as well as xenobiotic-host and/or xenobiotic-xenobiotic interactions. Upon linear velocity conditions *in vitro*, the depletion rate of ENT-A011 may be extrapolated to a. *in vivo* hepatic clearance, b. extraction ratio, and c. the effect of hepatic first-pass metabolism to total oral bioavailability. Biodegradation, metabolic, and toxicity liabilities can be identified early on and thus, inform structure-activity relationships (SAR).

For this, after the administration of ENT-A011 at 1 μM, the activity of CYP1A2, CYP2A6, CYP2B6, CYP2C9, CYP2C19, CYP2D6, and CYP3A4 (seven major human CYP450s) was assessed to determine a. the oxidative (CYP-mediated) metabolic stability profile in question and b. the enzyme metabolizing isoforms responsible (the test system consists of recombinant human CYP450 and CYP450 reductase; cytochrome b_5 may also be present).

Herein, no concentration-dependent effects are anticipated, as our model depicts direct interactions between ENT-A011 and CYP450 isoenzymes. CYP450 substrates are known to change enzyme conformation, disrupt enzyme structure and/or function and/or block the enzyme active site, altering enzyme[35,36]. First, the catalytic activity of CYP1A2, CYP2A6, CYP2B6, CYP2C9, CYP2C19, CYP2D6, and CYP3A4 was determined after the administration of ENT-A011 at 1 μM. Catalytic activity was

assessed on the basis of the relative fluorescence of the enzymatic reaction product (Figure S6_A). ENT-A011 did not show strong effects on the enzyme (catalytic) activity of the seven major CYP450-isozymes tested herein.

Next, CYP450 (%) inhibition was determined. For the CYP450 isoenzymes tested, no product inhibition or mechanism-based inactivation was obtained. No solubility issues were reported for ENT-A011 (new chemical entities with poor solubility may result in artificially low CYP450 inhibition and thus, potential drug-drug interaction toxicities may escape our attention). CYP450 enzyme inhibition may result in unexpectedly high exposure of co-administered xenobiotics and hence, increase the risk for adverse effects. As depicted in Figure S6_B, ENT-A011 exhibits weak inhibition for CYP2D6, while it is a moderate inhibitor for CYP2B6, CYP2C9, and CYP2C19. For all CYP450 isoforms tested, we also assessed CYP450 (%) metabolic activity, expressed as residual % of time zero. Our findings are depicted in Figure S6_C, according to which ENT-A011 was overall metabolically stable (from t=0 to t=60 minutes).

Herein, we performed an isozyme-specific CYP450-study to delineate ENT-A011 interactions with each of the seven major CYP450s that account for xenobiotic biodegradation, metabolism, and toxicity as well as xenobiotic-xenobiotic and/or xenobiotic-host interactions.

ENT-A011 acts as a weak-to-moderate CYP450 enzyme inhibitor, yet showing no biodegradation, liver metabolism or safety issues.

ENT-A011 exhibits very slow depletion in human liver microsomes

The susceptibility of a test-compound to biotransformation is defined as metabolic stability. For the ranking of test compounds, several approaches can be followed including parent structure loss during metabolic reactions or their intrinsic clearance (CL_{int}) and *in vitro* half-life ($t_{1/2}$) values[37,38]. Herein, we chose the former.

Parent structure loss is classified as very slow (<5 %), slow (5-19 %), moderate (20-50 %), fast (50-80 %) or very fast (>80 %). Such categories have been defined according to set criteria, namely, high metabolism ($t_{1/2}$ value of <30 min), moderate metabolism (30 min < $t_{1/2}$ value of < 60 min) and low metabolism ($t_{1/2}$ value of >60min).

ENT-A011 is very slowly depleted showing 93% residual of time zero at t=60 minutes. Thus, ENT-A011 may correspond to low or medium intrinsic clearance classification bands.

For humans, a low intrinsic clearance classification band is defined by an CL_{int} <8.6 µL/min/mg protein, whereas a high intrinsic clearance classification band is defined by an CL_{int} >47.0 µL/min/mg protein. Low clearance test-compounds are characterized by enhanced exposure, prolonged half-life and reduced doses, predicted as suitable for once-daily dosing.

BDNF and ENT-A011 treatment effects on expression of target genes related to neuronal function

Various enriched shared terms show that both treatments lead to a positive regulation of neurotransmitters and their release. This includes "chemical synaptic transmission" and "signal release from synapse", with relevant target genes such as Bassoon (*BSN*), Complexin-2 (*CPX-2)*), Rab3-interacting molecule 2 (*RIMS2*) and Synaptotagmin-7 (*SYT7*) upregulated by both molecules, and additional related genes upregulated by BDNF such as Cholinergic receptor nicotinic alpha 4 (*CHRNA4*), Cholinergic receptor nicotinic beta 4 (*CHRNB4*) and Synapsin-1 (*SYN1*) or by ENT-A011 such as Glutamate decarboxylase 1 (*GAD1*), Glutamate receptor ionotropic AMPA 2 (*GRIA2*) and Metabotropic glutamate receptor 4 (*GRM4*). *BSN* is involved in the organization of the presynaptic active zone and regulation of neurotransmitter release[39], *CPLX2* plays a role in synaptic vesicle exocytosis and neurotransmitter release by modulating SNARE complex assembly[40] and *RIMS2* regulates neurotransmitter release by interacting with Rab3 and modulating synaptic vesicle trafficking and docking[41]. What is more, *CHRNA4* and *CHRNB4* are structural components of acetylcholine receptors[42], while *GAD1*, *GRIA2* and *GRM4* are involved in glutamate signalling[43–45]. Additionally, some of these genes (e.g. *SYT7* and *RIMS2*) are specifically involved in calcium regulated neurotransmitter release ("calcium ion-regulated exocytosis of neurotransmitter" enriched for BDNF), with more examples of BDNF upregulated targets such as Heat shock 70kDa protein 5 (*HSPA5*) and Synaptotagmin-5 (*SYT5*) linked to "cellular response to calcium ion". From these examples, *SYT7* functions as a calcium sensor for neurotransmitter release and regulates synaptic vesicle fusion

with the plasma membrane[46]. Glutamate receptor ionotropic NMDA 3A (*GRIN3A*) (along other targets mentioned) is an additional BDNF target that is also related to “regulation of neurotransmitter levels”. ENT-A011 induced targets include more genes participating in "regulation of ion transmembrane transport / transporter activity", such as Ankyrin-3 (*ANK3*), Potassium voltage-gated channel subfamily D member 2 (*KCND2*), Copper-transporting ATPase 1 (*ATP7A*), Voltage-dependent L-type calcium channel subunit beta-3 (*CACNB3*) and Reelin (*RELN*), with the latter also involved in "neurotransmitter-gated ion channel clustering", along with other interesting targets such as SLIT and NTRK-like family member 3 (*SLITRK3*) and Shisa homolog 7 (*SHISA7*). Interesting among these targets involved in ion channel regulation, *ANK3* functions in anchoring ion channels and transporters to the cytoskeleton[47], *KCND2* is a component of potassium channels[48] and *CACNB3* is part of calcium channels[49] and *ATP7A* is involved in copper transport across membranes[50].

Markers of iPSC-derived cortical neuronal progenitor identity

Immunostaining for selected markers (Sox2, Tbr2) to confirm differentiation quality is provided in Figure S13. Moreover, the expression of relevant markers is confirmed in the RNAseq data, including multipotency markers that are expected to be expressed in progenitors such as SOX2, PAX6, EOMES (Tbr2), FOXG1 and Nestin, as well as markers of neural differentiation such as MAP2, TUBB3 (Tuj1) and cortical markers TBR1, CUX1 and POU3F2 (Brn2). At the same time, iPSC markers POU5F1 (Oct4) and Nanog, or astrocytic marker GFAP are not expressed at detectable levels.

Supplementary Figures

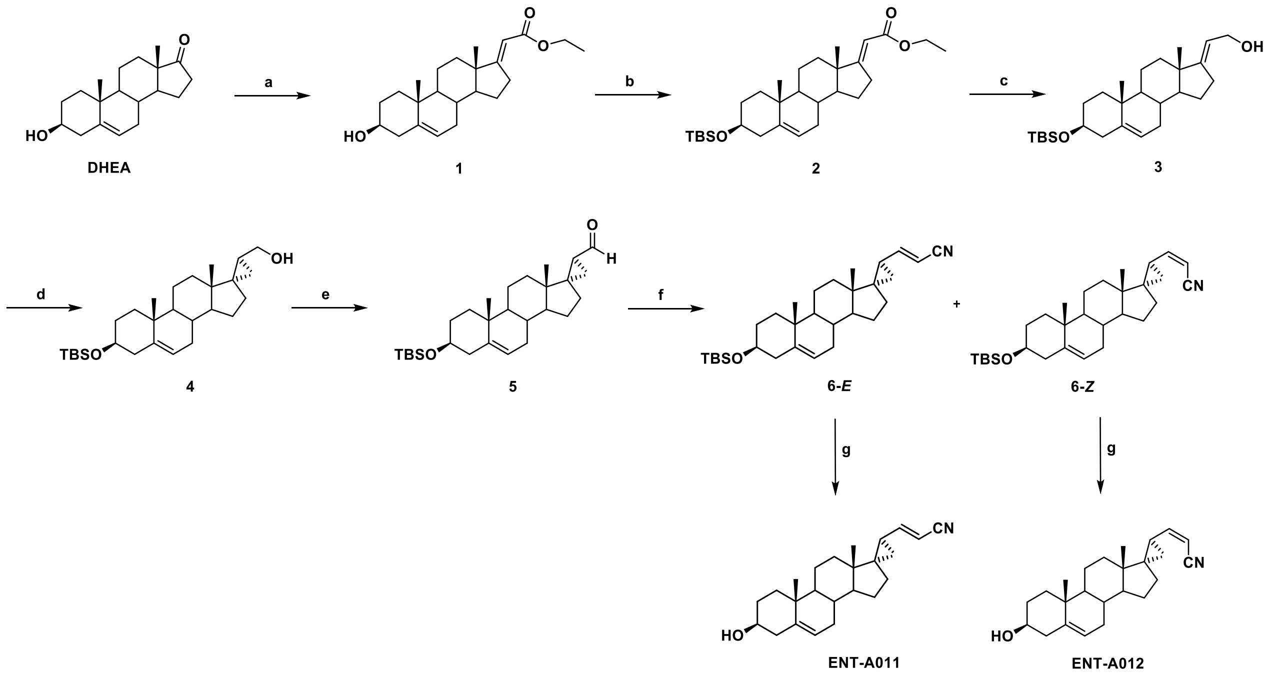

Scheme 1. Synthesis of ENT-A011 and ENT-A012. Reagents and conditions: (a) $(CH_3CH_2O)_2P(O)CH_2C(O)OCH_2CH_3$, EtONa, THF/EtOH 1:1, reflux, overnight; (b) TBSCl, Imidazole, I_2, THF, 0 °C to 25 °C, overnight; (c) DIBAL-H, CH_2Cl_2, −78 °C, 2.5 h; (d) CH_2I_2, Et_2Zn, −78 °C to 25 °C, 1 h; (e) DMP,CH_2Cl_2, 0 °C to 25 °C, 1.5 h; (f) $(CH_3CH_2O)_2P(O)CH_2CN$, NaH, THF, 0 °C to 25 °C, 0.5 h; (g) HF·Pyridine, CH_2Cl_2, 0 °C to 25 °C, 1 h.

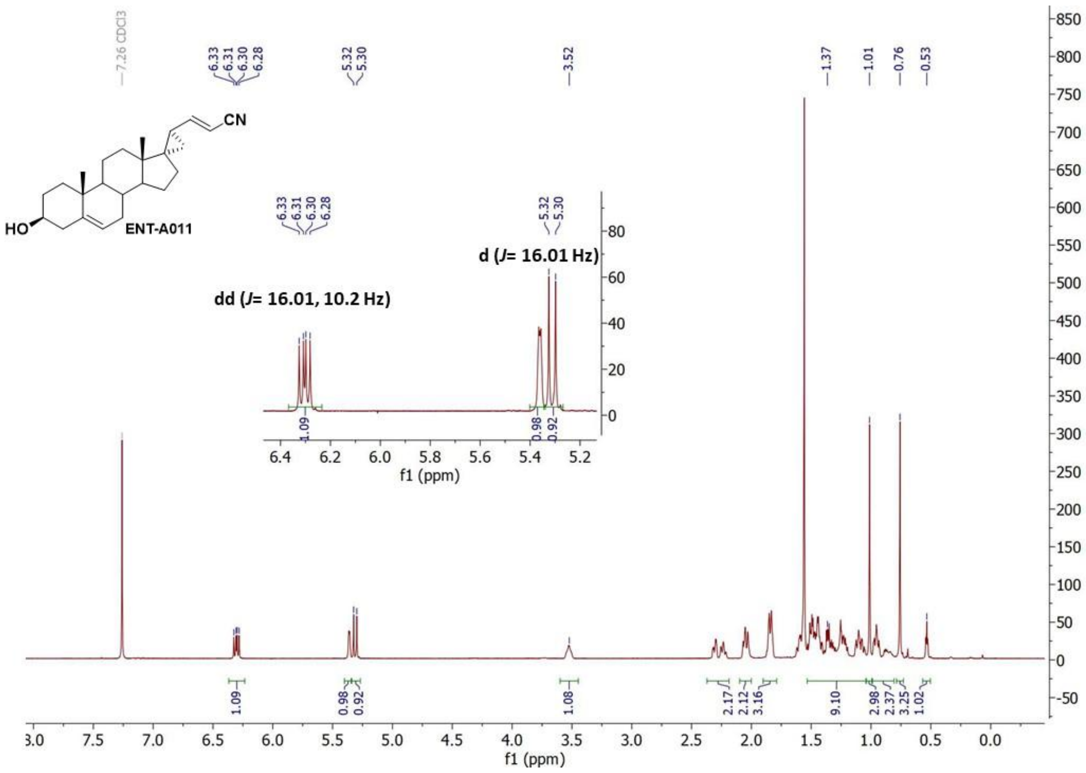

Figure S1. **^{1}H-NMR spectrum of ENT-A011 in $CDCl_3$.**

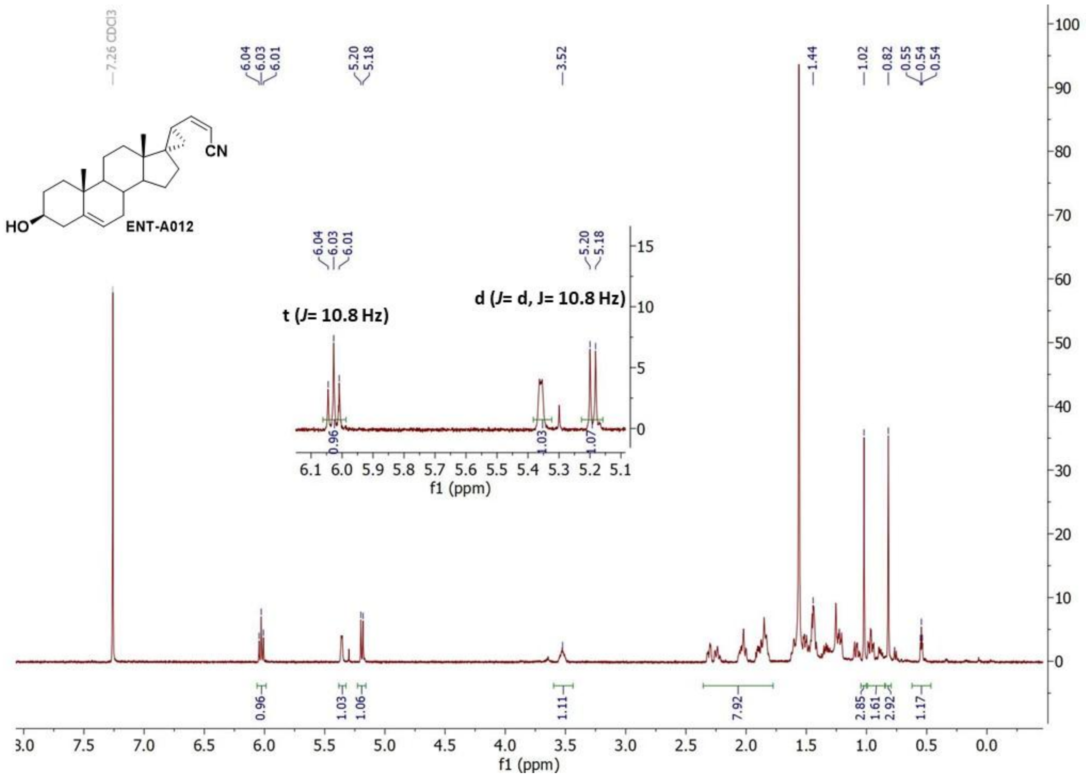

Figure S2. **^{1}H-NMR spectrum of ENT-A012 in $CDCl_3$.**

Area % Report

Data File:
C:\ChromQuest\Enterprise\Projects\Default\Method\TC\Alessia\ENT-A011_MeOH-H2O-01%HCOOH_25min-237nm.dat
Method:
C:\ChromQuest\Enterprise\Projects\Default\Method\TC\Alessia\Gradient_MeOH_H2O_0.1%HCOOH_25min_237nm_95-5to100_2.met
Acquired: 11/1/2021 2:33:08 PM
Printed: 11/1/2021 3:01:00 PM

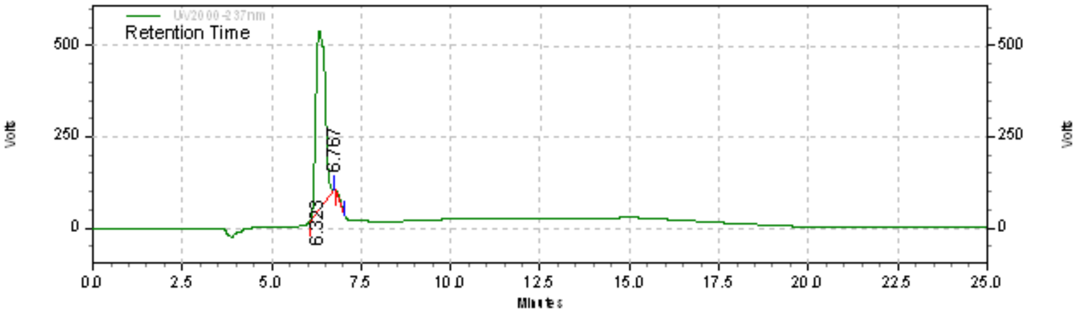

UV2000-237nm Results (System (11/1/2021 3:00:57 PM) (Reprocessed))

Retention Time	Area	Area %	Height	Height %
6.323	8678720	99.09	489972	100.00
6.767	79379	0.91	0	0.00
Totals	8758099	100.00	489972	100.00

Figure S3. HPLC chromatogram of compound ENT-A011. The purity of ENT-A011 was determined by high-performance liquid chromatography (HPLC) using Nucelosil 100-5 C18 HD column, 5µm (4.6 x 250 mm), flow rate 1 mL/min, eluting with H_2O, 0.1% HCOOH – MeOH, 0.1% HCOOH gradient employing UV detection at 237 nm. t_R = 6.32 min, purity = 99.09%.

Area % Report

Data File:
C:\ChromQuest\Enterprise\Projects\Default\Method\TC\Alessia\ENT-A012_MeOH-H2O-01%HCOOH_25min-237nm.dat
Method:
C:\ChromQuest\Enterprise\Projects\Default\Method\TC\Alessia\Gradient_MeOH_H2O_0.1%HCOOH_30min.met

Acquired: 11/1/2021 3:07:18 PM
Printed: 11/1/2021 3:42:50 PM

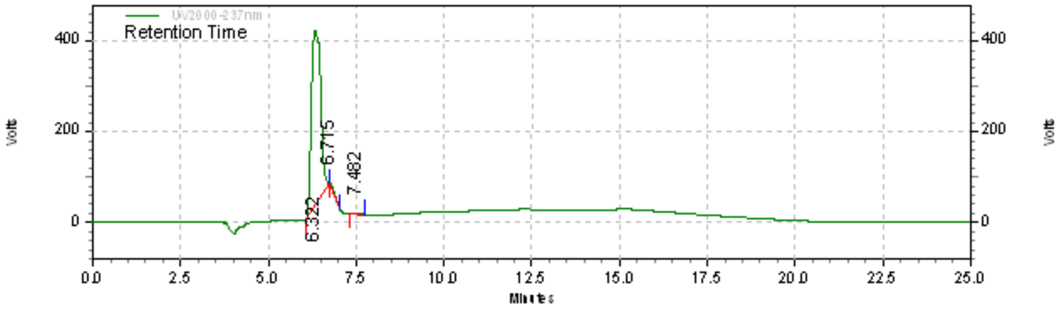

UV2000-237nm Results (System (11/1/2021 3:42:43 PM) (Reprocessed))

Retention Time	Area	Area %	Height	Height %
6.322	7009356	98.47	387982	99.62
6.715	84181	1.18	0	0.00
7.482	24607	0.35	1483	0.38
Totals	7118144	100.00	389465	100.00

Figure S4. **HPLC chromatogram of compound ENT-A012.** The purity of ENT-A012 was determined by high-performance liquid chromatography (HPLC) using Nucelosil 100-5 C18 HD column, 5μm (4.6 x 250 mm), flow rate 1 mL/min, eluting with H_2O, 0.1% HCOOH – MeOH, 0.1% HCOOH gradient employing UV detection at 237 nm. t_R = 6.32 min, purity = 98.47%.

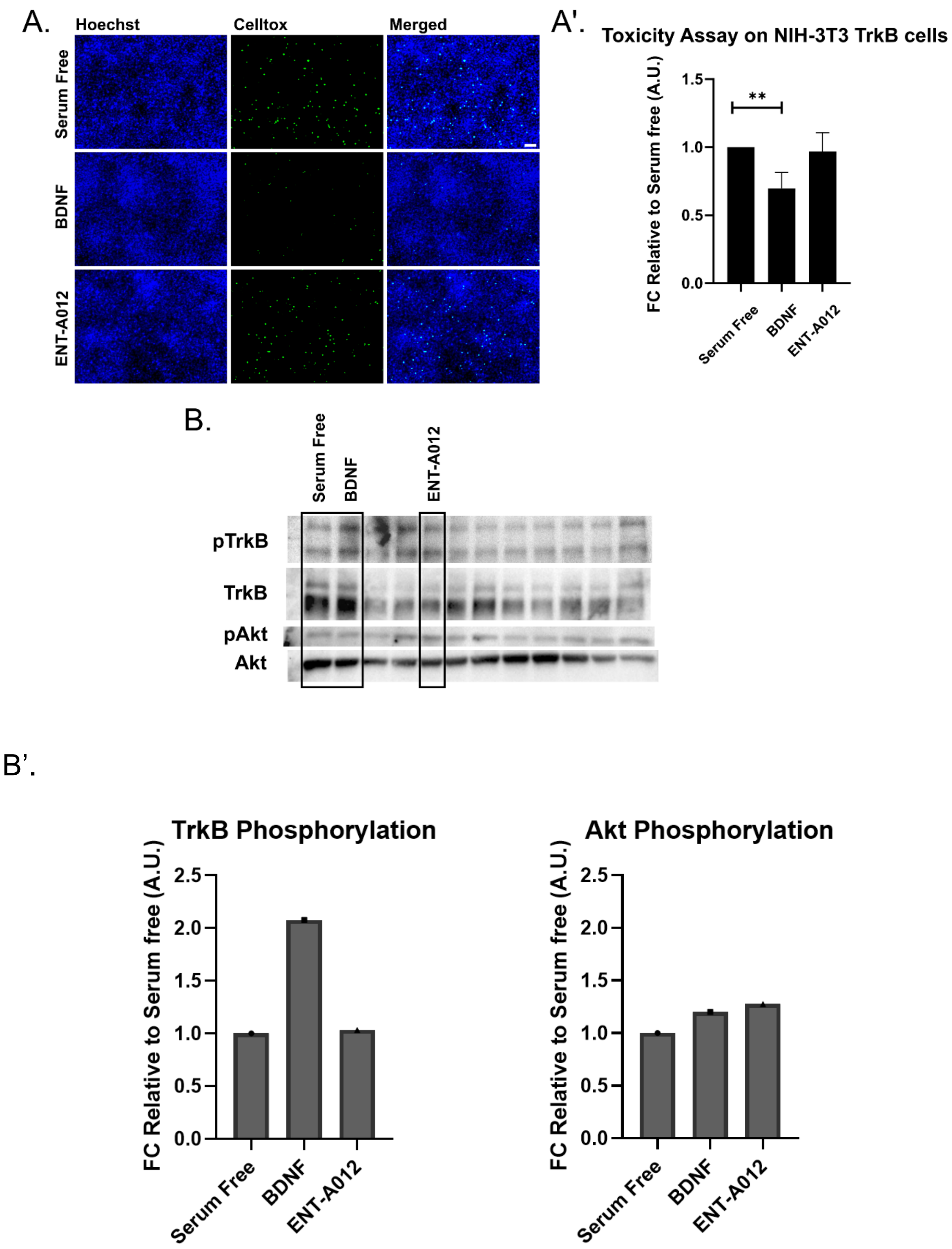
A.
Hoechst
Celltox
Merged
Serum Free
BDNF
ENT-A012
A'.
Toxicity Assay on NIH-3T3 TrkB cells
FC Relative to Serum free (A.U.)
1.5
1.0
0.5
0.0
**
Serum Free
BDNF
ENT-A012
B.
Serum Free
BDNF
ENT-A012
pTrkB
TrkB
pAkt
Akt
B'.
TrkB Phosphorylation
FC Relative to Serum free (A.U.)
2.5
2.0
1.5
1.0
0.5
0.0
Serum Free
BDNF
ENT-A012
Akt Phosphorylation
FC Relative to Serum free (A.U.)
2.5
2.0
1.5
1.0
0.5
0.0
Serum Free
BDNF
ENT-A012

Figure S5. **ENT-A012 does not reduce cell death caused by serum deprivation in NIH-3T3 TrkB cells**. Representative images (A) and quantification of Toxicity Assay (A') in NIH-3T3 TrkB cells after treatment with BDNF or compound ENT-A012 for 24h in serum free conditions. N=13, error bars represent S.E.M., Student's t-test against Control; **<0.01. The compound ENT-A012 do not induce TrkB and its downstream target Akt phosphorylation after 20 minutes treatment in NIH-3T3 TrkB stable expressed cells, representative blot (B) and quantification of TrkB and Akt Phosphorylation (B'). Scalebar = 100µm

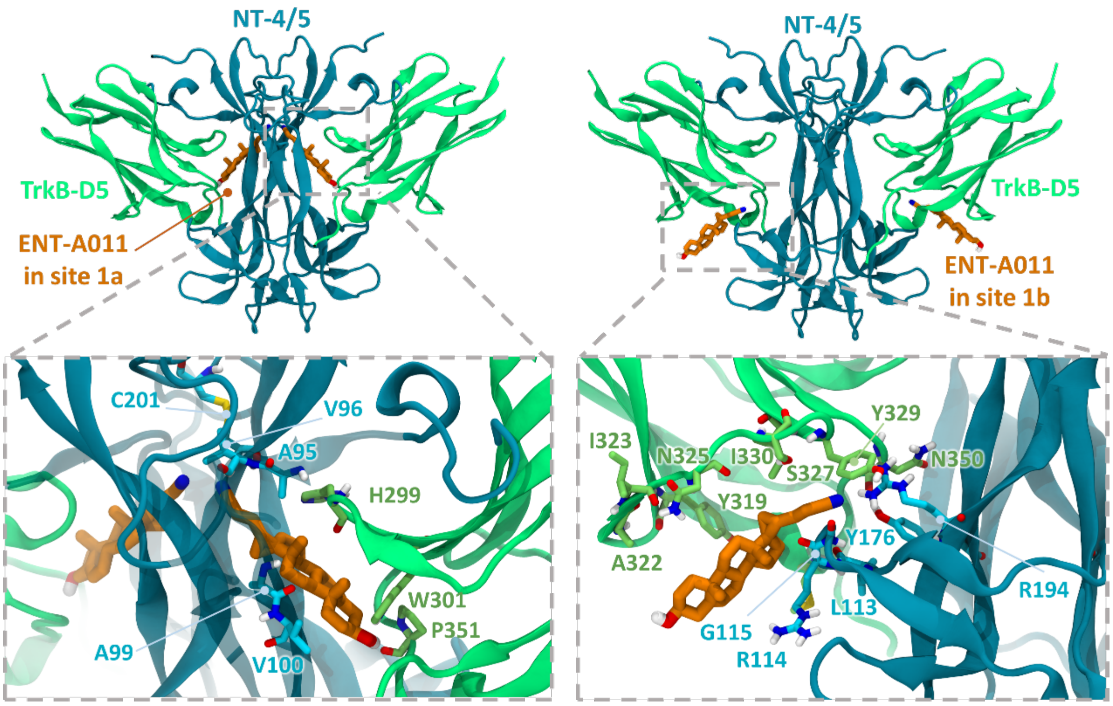

Figure S6: Docking poses of compound ENT-A011 in sites 1a and 1b at the two symmetry-related interfaces of the NT-4/5 -TrkB-D5 neurotrophin-receptor complex. The close-up views show residues lining the two sites. TrkB-D5 and NT-4/5 are shown, respectively, in green and blue cartoon representation with selected residues in stick representation colored by atom type. The ENT-A011 compound is shown in stick representation colored by atom type with orange carbons.

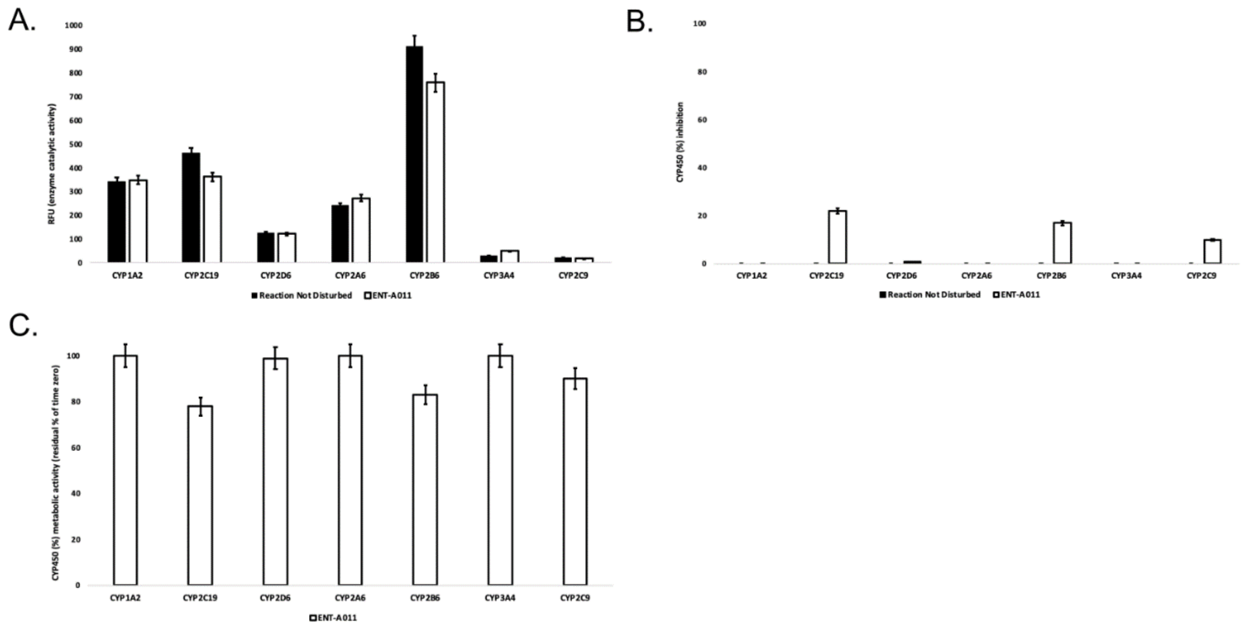

Figure S7. A. Enzyme (catalytic) activity of the CYP1A2, CYP2A6, CYP2B6, CYP2C9, CYP2C19, CYP2D6, and CYP33A4 isoenzymes, following ENT-A011 administration at 1 μM (t=60 minutes). RFU: relative fluorescence units. Reaction Not Disturbed: reaction without ENT-A011. B. CYP450 (%) inhibition per isozyme tested after the administration of ENT-A011 at 1 μM (t=60 minutes). C. CYP450 (%) metabolic activity of CYP1A2, CYP2A6, CYP2B6, CYP2C9, CYP2C19, CYP2D6, and CYP33A4 isozymes, following ENT-A011 administration at 1 μM (t=60 minutes).

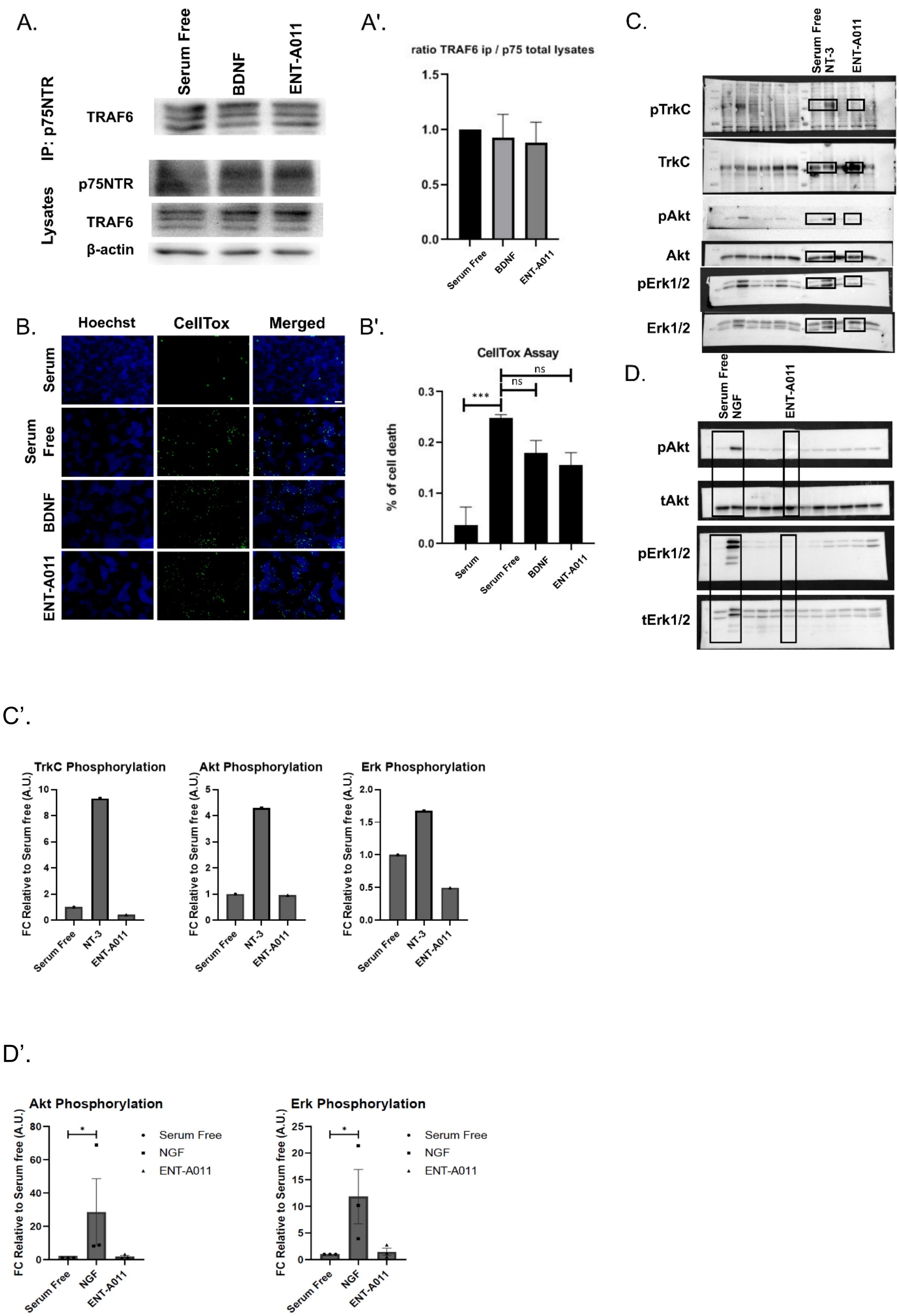
A.
IP: p75NTR
Lysates
Serum Free
BDNF
ENT-A011
TRAF6
p75NTR
TRAF6
β-actin
A'.
ratio TRAF6 ip / p75 total lysates
Serum Free
BDNF
ENT-A011
C.
Serum Free
NT-3
ENT-A011
pTrkC
TrkC
pAkt
Akt
pErk1/2
Erk1/2
B.
Hoechst
CellTox
Merged
Serum
Serum Free
BDNF
ENT-A011
B'.
CellTox Assay
% of cell death
***
ns
ns
Serum
Serum Free
BDNF
ENT-A011
D.
Serum Free
NGF
ENT-A011
pAkt
tAkt
pErk1/2
tErk1/2
C'.
TrkC Phosphorylation
Akt Phosphorylation
Erk Phosphorylation
FC Relative to Serum free (A.U.)
Serum Free
NT-3
ENT-A011
D'.
Akt Phosphorylation
Erk Phosphorylation
FC Relative to Serum free (A.U.)
*
Serum Free
NGF
ENT-A011

Figure S8. **ENT-A011 does not activate the p75, TrkA and TrkC pathways.** A. HEK293T cells were co-transfected with the plasmid cDNAs of p75NTR and TRAF6. Transfectants were exposed for 20 min to BDNF (500ng/ml) & the tested compound ENT-A011 (1μM) and lysates were immunoprecipitated with p75NTR-specific antibodies and then immunoblotted with antibodies against TRAF6. Total lysates were analysed for p75NTR, TRAF6 and actin expression by immunobloting. A'. Quantification shows that ENT-A011 does not activate p75NTR *(one way ANOVA, no significance, Mean±SEM of triplicate measurements)*. B. HEK cells transfected with p75NTR were starved from serum and treated with ENT-A011 (1μM) or BDNF (500ng/ml) for 24hrs and subsequently subjected to CellTox assay. B'. Quantification shows that there is no significant difference between ENT-A011 treated group and negative control, Serum free. C. NIH-3T3 TrkC stable transfected cells were treated with Neurotrophin-3 (NT-3) or ENT-A011 for 20min and the lysates were immunoblotted with antibodies against pTrkC, TrkC, pAkt, Akt, pErk1/2, Erk1/2. Quantification of TrkC, Akt and Erk Phosphorylation (C') western blots. The compound ENT-A011 did not phosphorylate TrkC neither activate the downstream pathway D. PC12 cells were treated with NGF or ENT-A011 for 20 min, lysates were immunoblotted with pAkt, Akt, pErk1/2, Erk1/2. D'. Quantification of Akt and Erk Phosphorylation western blots. The TrkA signaling was not activated by the compound ENT-A011 in contrast to NGF. Scalebar = 100μm

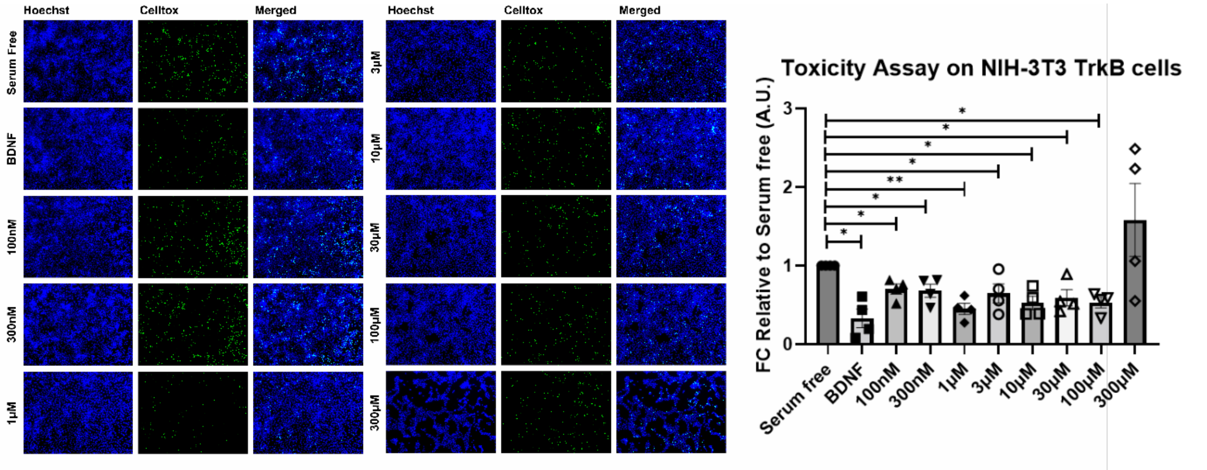

Figure S9. **Dose response toxicity assay on NIH-3T3 TrkB cells**. Left: Representative fluorescence microscopy images of Celltox cytotoxicity assay on NIH-3T3 TrkB cells treated with BDNF or increasing concentrations of ENT-A011 (100nM, 300nM, 1μM, 3μM, 10μM, 30μM, 100μM, 300μM). Right: Quantification represents Celltox positive cells normalized against Hoechst positive cells. N=4, error bars represent S.E.M., Student's t-test against Control; *<0.05, **<0.01.

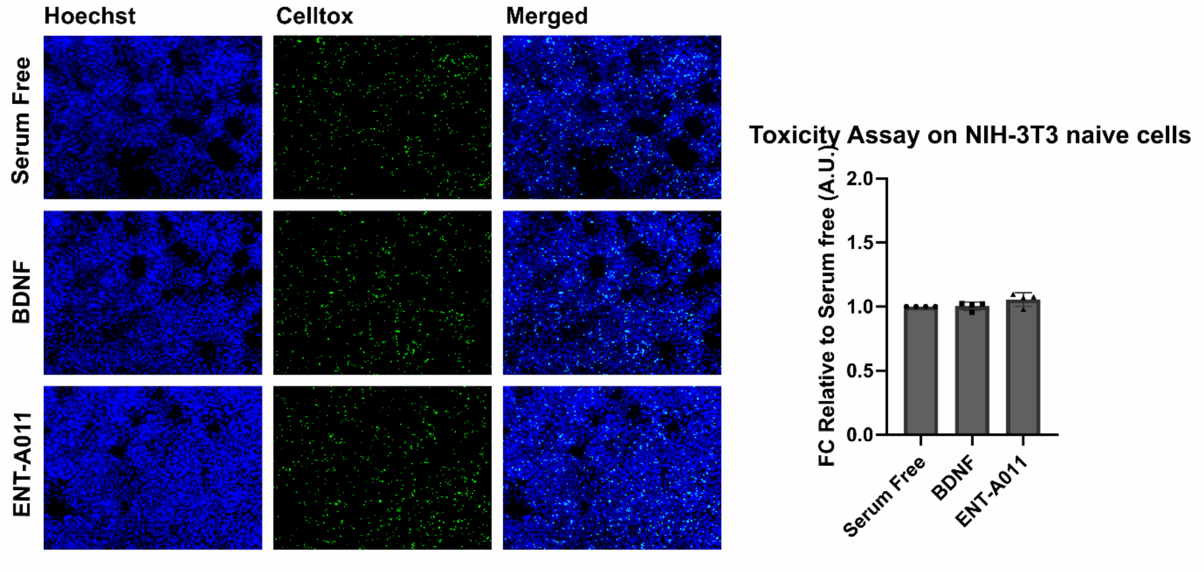

Figure S10. **Toxicity assay on naïve NIH-3T3 cells**. Left: Representative fluorescence microscopy images of Celltox cytotoxicity assay on naïve NIH-3T3 cells treated with BDNF or ENT-A011 (1μM). Right: Quantification represents Celltox positive cells normalized against Hoechst positive cells. N=4, error bars represent S.E.M.

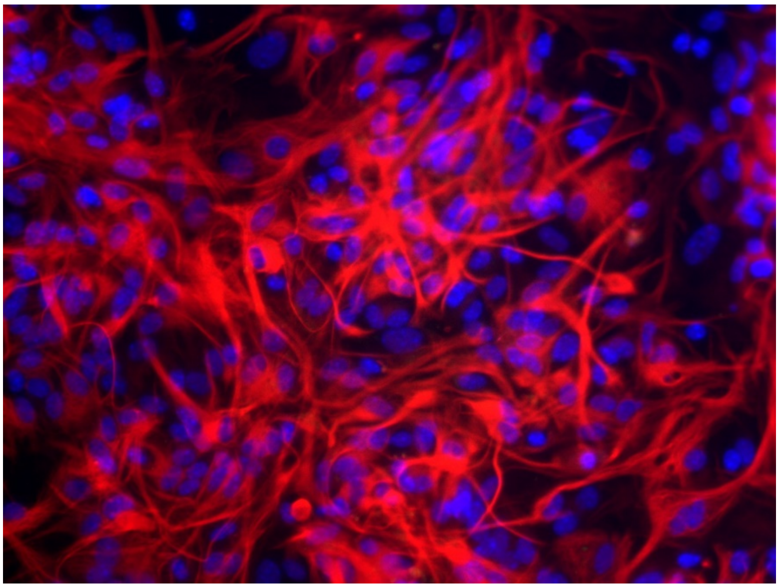

Figure S11. **Immunostaining of astrocytes cultures used in the current study against GFAP (red) and DAPI staining (blue)**.

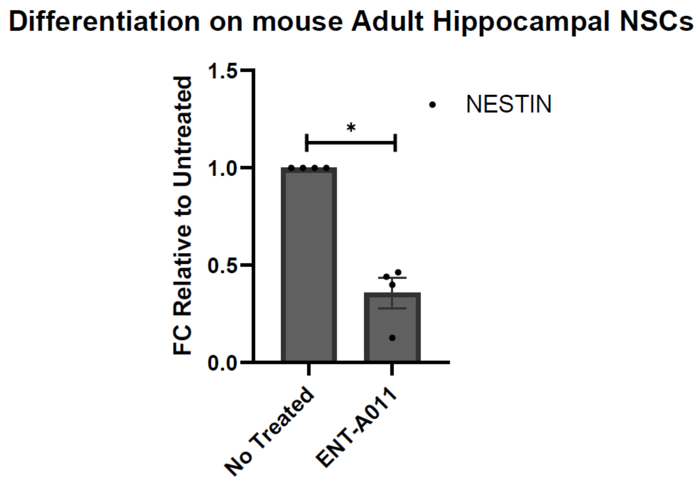

Figure S12 Fold change in expression of nestin (N=4), assessed by RT-qPCR in ENT-A011 treated adult hippocampal NSCs relative to untreated control cells. Student's t-test against Control; *<0.05

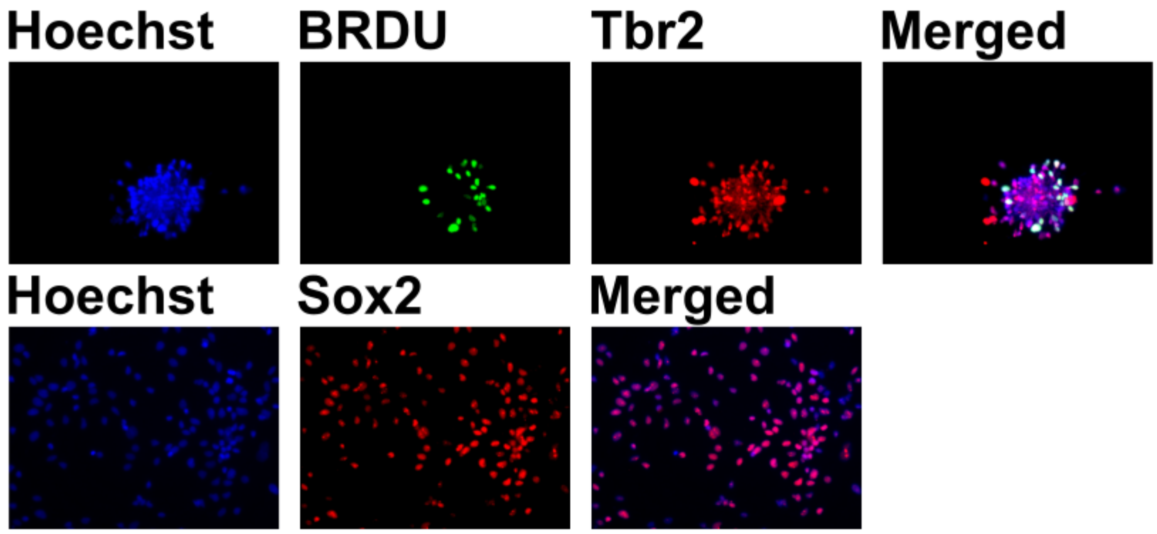

Figure S13 Immunostaining for markers Tbr2 (top) and Sox2 (bottom) in cortical NPCs differentiated from human iPSCs.

Supplementary Tables

Supplementary Table 1. Primer sequences used for Quantitative RT-PCR

Gene	Primer
mActin-F	GGAGATTACTGCTCTGGCTC
mActin-R	GGACTCATCGTACTCCTGCT
MAP2-F	CGCAAAACCACAGCAGCAAG
MAP2-R	GGGAGGATGGAGGAAGGTCT
Tubb3-F	CAGATAGGGGCCAAGTTCTGG
Tubb3-R	GTCCCCTACATAGTTGCCGC
GFAP-F	AGAAAGGTTGAATCGCTGGA
GFAP-R	CGGCGATAGTCGTTAGCTTC

Supplementary Table 2. Counts (Htseq-count) for *BDNF* and *NTRK2* (TrkB) genes. Both genes were not found to be differentially regulated by EdgeR.

	BDNF	NTRK2
ENTA011_AVERAGE	2.333	116.666
BDNF_AVERAGE	3	86.666
untreated_AVERAGE	3	107
ENTA011_rep1	2	100
ENTA011_rep2	1	111
ENTA011_rep3	4	139
BDNF_rep1	5	102
BDNF_rep2	1	101
BDNF_rep3	3	57
untreated_rep1	2	102
untreated_rep2	2	151
untreated_rep3	5	68

Additional Files

Additional file 1 EdgeR results from RNA-seq of ENT-A011 and BDNF treated iPSC derived NPCs

Additional file 2 Metascape Ontology Enrichment on upregulated targets of ENT-A011 and BDNF in iPSC derived NPCs RNA-seq

Additional file 3 Metascape Ontology Enrichment of downregulated targets of ENT-A011 and BDNF in iPSC derived NPCs RNA-seq

Additional file 4 Western blots used for quantification of TrkB phosphorylation in NIH-3T3 cells and astrocytes.

Additional References

1. Banfield MJ, Naylor RL, Robertson AGS, Allen SJ, Dawbarn D, Brady RL. Specificity in Trk Receptor:Neurotrophin Interactions. *Structure*. 2001;9(12):1191-1199. doi:10.1016/S0969-2126(01)00681-5
2. Sali A, Blundell TL. Comparative protein modelling by satisfaction of spatial restraints. *J Mol Biol*. 1993;234(3):779-815. doi:10.1006/jmbi.1993.1626
3. Robinson RC, Choe S, Radziejewski C, et al. The structures of the neurotrophin 4 homodimer and the brain-derived neurotrophic factor/neurotrophin 4 heterodimer reveal a common Trk-binding site. *Protein Science*. 2008;8(12):2589-2597. doi:10.1110/ps.8.12.2589
4. Madhavi Sastry G, Adzhigirey M, Day T, Annabhimoju R, Sherman W. Protein and ligand preparation: parameters, protocols, and influence on virtual screening enrichments. *J Comput Aided Mol Des*. 2013;27(3):221-234. doi:10.1007/s10822-013-9644-8
5. Schrödinger Release 2020-2: Protein Preparation Wizard; Epik, Schrödinger, LLC, New York, NY, 2020; Impact, Schrödinger, LLC, New York, NY; Prime, Schrödinger, LLC, New York, NY, 2020.
6. Olsson MHM, Søndergaard CR, Rostkowski M, Jensen JH. PROPKA3: Consistent Treatment of Internal and Surface Residues in Empirical p K a Predictions. *J Chem Theory Comput*. 2011;7(2):525-537. doi:10.1021/ct100578z
7. Harder E, Damm W, Maple J, et al. OPLS3: A Force Field Providing Broad Coverage of Drug-like Small Molecules and Proteins. *J Chem Theory Comput*. 2016;12(1):281-296. doi:10.1021/acs.jctc.5b00864
8. Schrödinger Release 2020-2: SiteMap, Schrödinger, LLC, New York, NY, 2020.
9. Halgren TA. Identifying and Characterizing Binding Sites and Assessing Druggability. *J Chem Inf Model*. 2009;49(2):377-389. doi:10.1021/ci800324m
10. Halgren T. New Method for Fast and Accurate Binding-site Identification and Analysis. *Chem Biol Drug Des*. 2007;69(2):146-148. doi:10.1111/j.1747-0285.2007.00483.x

11. Pediaditakis I, Efstathopoulos P, Prousis KC, et al. Selective and differential interactions of BNN27, a novel C17-spiroepoxy steroid derivative, with TrkA receptors, regulating neuronal survival and differentiation. *Neuropharmacology*. 2016;111(January 2018):266-282. doi:10.1016/j.neuropharm.2016.09.007
12. Schrödinger Release 2020-2: Maestro, Schrödinger, LLC, New York, NY, 2020.
13. Schrödinger Release 2020-2: LigPrep, Schrödinger, LLC, New York, NY, 2020.
14. Friesner RA, Banks JL, Murphy RB, et al. Glide: A New Approach for Rapid, Accurate Docking and Scoring. 1. Method and Assessment of Docking Accuracy. *J Med Chem*. 2004;47(7):1739-1749. doi:10.1021/jm0306430
15. Halgren TA, Murphy RB, Friesner RA, et al. Glide: A New Approach for Rapid, Accurate Docking and Scoring. 2. Enrichment Factors in Database Screening. *J Med Chem*. 2004;47(7):1750-1759. doi:10.1021/jm030644s
16. Friesner RA, Murphy RB, Repasky MP, et al. Extra Precision Glide: Docking and Scoring Incorporating a Model of Hydrophobic Enclosure for Protein−Ligand Complexes. *J Med Chem*. 2006;49(21):6177-6196. doi:10.1021/jm051256o
17. Schrödinger Release 2020-2: Glide, Schrödinger, LLC, New York, NY, 2020.
18. Farid R, Day T, Friesner RA, Pearlstein RA. New insights about HERG blockade obtained from protein modeling, potential energy mapping, and docking studies. *Bioorg Med Chem*. 2006;14(9):3160-3173. doi:10.1016/j.bmc.2005.12.032
19. Sherman W, Day T, Jacobson MP, Friesner RA, Farid R. Novel Procedure for Modeling Ligand/Receptor Induced Fit Effects. *J Med Chem*. 2006;49(2):534-553. doi:10.1021/jm050540c
20. Sherman W, Beard HS, Farid R. Use of an Induced Fit Receptor Structure in Virtual Screening. *Chemical Biology <html_ent glyph="@amp;" ascii="&"/> Drug Design*. 2006;67(1):83-84. doi:10.1111/j.1747-0285.2005.00327.x
21. Schrödinger Release 2020-2: Induced Fit Docking protocol; Glide, Schrödinger, LLC, New York, NY, 2020; Prime, Schrödinger, LLC, New York, NY, 2020.
22. Schrödinger Release 2020-2: Prime, Schrödinger, LLC, New York, NY, 2020.
23. Jacobson MP, Friesner RA, Xiang Z, Honig B. On the Role of the Crystal Environment in Determining Protein Side-chain Conformations. *J Mol Biol*. 2002;320(3):597-608. doi:10.1016/S0022-2836(02)00470-9
24. Jacobson MP, Pincus DL, Rapp CS, et al. A hierarchical approach to all-atom protein loop prediction. *Proteins: Structure, Function, and Bioinformatics*. 2004;55(2):351-367. doi:10.1002/prot.10613
25. Humphrey W, Dalke A, Schulten K. VMD: Visual molecular dynamics. *J Mol Graph*. 1996;14(1):33-38. doi:10.1016/0263-7855(96)00018-5
26. Toshiro Ibuka, Tooru Taga, Tetsuro Shingu, Masako Saito SN and YY. New Stereoselective Synthesis of 20S and 20J2 Steroidal Side Chains. Remarkable Stereoselectivity Differences between Saturated and ,/8-Unsaturated Steroidal Esters. *Journal of Organic Chemistry*. 1988;53(17):3947-3952.
27. Wicha J, Bal K, Piekut S. Synthesis of Pregn-17/20/-en-21-oic Acid Derivatives. The Wittig-Horner Reaction on Steroidal 17-Ketones. *Synth Commun*. 1977;7(3):215-222. doi:10.1080/00397917708050738

28. Pellissier H. Recent developments in asymmetric cyclopropanation. *Tetrahedron*. 2008;64(30-31):7041-7095. doi:10.1016/j.tet.2008.04.079
29. Kim HY, Salvi L, Carroll PJ, Walsh PJ. Highly Enantio- and Diastereoselective One-Pot Methods for the Synthesis of Halocyclopropyl Alcohols. *J Am Chem Soc*. 2009;131(3):954-962. doi:10.1021/ja806989n
30. De Boeck B, Herbert NMA, Harrington-Frost NM, Pattenden G. Vinylcyclopropylacyl and polyeneacyl radicals. Intramolecular ketene alkyl radical additions in ring synthesis. *Org Biomol Chem*. 2005;3(2):328. doi:10.1039/b413816n
31. Jang SW, Liu X, Chan CB, et al. Amitriptyline is a TrkA and TrkB Receptor Agonist that Promotes TrkA/TrkB Heterodimerization and Has Potent Neurotrophic Activity. *Chem Biol*. 2009;16(6):644-656. doi:10.1016/j.chembiol.2009.05.010
32. Shoemark DK, Williams C, Fahey MS, et al. Design and Nuclear Magnetic Resonance (NMR) Structure Determination of the Second Extracellular Immunoglobulin Tyrosine Kinase A (TrkAIg2) Domain Construct for Binding Site Elucidation in Drug Discovery. *J Med Chem*. 2015;58(2):767-777. doi:10.1021/jm501307e
33. Scarpi D, Cirelli D, Matrone C, et al. Low molecular weight, non-peptidic agonists of TrkA receptor with NGF-mimetic activity. *Cell Death Dis*. 2012;3(7):e339-e339. doi:10.1038/cddis.2012.80
34. Pediaditakis I, Efstathopoulos P, Prousis KC, et al. Selective and differential interactions of BNN27, a novel C17-spiroepoxy steroid derivative, with TrkA receptors, regulating neuronal survival and differentiation. *Neuropharmacology*. 2016;111:266-282. doi:10.1016/j.neuropharm.2016.09.007
35. Pelkonen O, Turpeinen M. In vitro–in vivo extrapolation of hepatic clearance: Biological tools, scaling factors, model assumptions and correct concentrations. *Xenobiotica*. 2007;37(10-11):1066-1089. doi:10.1080/00498250701620726
36. Zanger UM, Schwab M. Cytochrome P450 enzymes in drug metabolism: Regulation of gene expression, enzyme activities, and impact of genetic variation. *Pharmacol Ther*. 2013;138(1):103-141. doi:10.1016/j.pharmthera.2012.12.007
37. Barter Z, Bayliss M, Beaune P, et al. Scaling Factors for the Extrapolation of In Vivo Metabolic Drug Clearance From In Vitro Data: Reaching a Consensus on Values of Human Micro-somal Protein and Hepatocellularity Per Gram of Liver. *Curr Drug Metab*. 2007;8(1):33-45. doi:10.2174/138920007779315053
38. Masimirembwa CM, Bredberg U, Andersson TB. Metabolic Stability for Drug Discovery and Development. *Clin Pharmacokinet*. 2003;42(6):515-528. doi:10.2165/00003088-200342060-00002
39. Wang X, Hu B, Zieba A, et al. A Protein Interaction Node at the Neurotransmitter Release Site: Domains of Aczonin/Piccolo, Bassoon, CAST, and Rim Converge on the N-Terminal Domain of Munc13-1. *The Journal of Neuroscience*. 2009;29(40):12584-12596. doi:10.1523/JNEUROSCI.1255-09.2009

40. Reim K, Mansour M, Varoqueaux F, et al. Complexins Regulate a Late Step in Ca2+-Dependent Neurotransmitter Release. *Cell*. 2001;104(1):71-81. doi:10.1016/S0092-8674(01)00192-1
41. Wang Y, Liu X, Biederer T, Südhof TC. A family of RIM-binding proteins regulated by alternative splicing: Implications for the genesis of synaptic active zones. *Proceedings of the National Academy of Sciences*. 2002;99(22):14464-14469. doi:10.1073/pnas.182532999
42. Gotti C, Clementi F. Neuronal nicotinic receptors: from structure to pathology. *Prog Neurobiol*. 2004;74(6):363-396. doi:10.1016/j.pneurobio.2004.09.006
43. Kaufman DL, Houser CR, Tobin AJ. Two Forms of the γ-Aminobutyric Acid Synthetic Enzyme Glutamate Decarboxylase Have Distinct Intraneuronal Distributions and Cofactor Interactions. *J Neurochem*. 1991;56(2):720-723. doi:10.1111/j.1471-4159.1991.tb08211.x
44. Schoepp DD, Jane DE, Monn JA. Pharmacological agents acting at subtypes of metabotropic glutamate receptors. *Neuropharmacology*. 1999;38(10):1431-1476. doi:10.1016/S0028-3908(99)00092-1
45. Paoletti P, Bellone C, Zhou Q. NMDA receptor subunit diversity: impact on receptor properties, synaptic plasticity and disease. *Nat Rev Neurosci*. 2013;14(6):383-400. doi:10.1038/nrn3504
46. Bacaj T, Wu D, Yang X, et al. Synaptotagmin-1 and Synaptotagmin-7 Trigger Synchronous and Asynchronous Phases of Neurotransmitter Release. *Neuron*. 2013;80(4):947-959. doi:10.1016/j.neuron.2013.10.026
47. Jenkins SM, Bennett V. Ankyrin-G coordinates assembly of the spectrin-based membrane skeleton, voltage-gated sodium channels, and L1 CAMs at Purkinje neuron initial segments. *J Cell Biol*. 2001;155(5):739-746. doi:10.1083/jcb.200109026
48. Malin SA, Nerbonne JM. Molecular Heterogeneity of the Voltage-Gated Fast Transient Outward K^+ Current, I_{Af}, in Mammalian Neurons. *The Journal of Neuroscience*. 2001;21(20):8004-8014. doi:10.1523/JNEUROSCI.21-20-08004.2001
49. Dolphin AC. Calcium channel auxiliary α2δ and β subunits: trafficking and one step beyond. *Nat Rev Neurosci*. 2012;13(8):542-555. doi:10.1038/nrn3311
50. Lutsenko S, Barnes NL, Bartee MY, Dmitriev OY. Function and Regulation of Human Copper-Transporting ATPases. *Physiol Rev*. 2007;87(3):1011-1046. doi:10.1152/physrev.00004.2006
